# Neuromodulatory effects of transcranial magnetic stimulation on language performance in healthy participants: Systematic review and meta-analysis

**DOI:** 10.3389/fnhum.2022.1027446

**Published:** 2022-12-05

**Authors:** Xingfang Qu, Zichao Wang, Yao Cheng, Qingwei Xue, Zimu Li, Lu Li, Liping Feng, Gesa Hartwigsen, Luyao Chen

**Affiliations:** ^1^Max Planck Partner Group, School of International Chinese Language Education, Beijing Normal University, Beijing, China; ^2^Lise Meitner Research Group Cognition and Plasticity, Max Planck Institute for Human Cognitive and Brain Sciences, Leipzig, Germany

**Keywords:** language, healthy participants, TMS, neuromodulatory effect, meta-analysis, non-invasive brain stimulation

## Abstract

**Background:**

The causal relationships between neural substrates and human language have been investigated by transcranial magnetic stimulation (TMS). However, the robustness of TMS neuromodulatory effects is still largely unspecified. This study aims to systematically examine the efficacy of TMS on healthy participants’ language performance.

**Methods:**

For this meta-analysis, we searched *PubMed*, *Web of Science*, *PsycINFO*, *Scopus*, and *Google Scholar* from database inception until October 15, 2022 for eligible TMS studies on language comprehension and production in healthy adults published in English. The quality of the included studies was assessed with the Cochrane risk of bias tool. Potential publication biases were assessed by funnel plots and the Egger Test. We conducted overall as well as moderator meta-analyses. Effect sizes were estimated using Hedges’*g* (*g*) and entered into a three-level random effects model.

**Results:**

Thirty-seven studies (797 participants) with 77 effect sizes were included. The three-level random effects model revealed significant overall TMS effects on language performance in healthy participants (RT: *g* = 0.16, 95% CI: 0.04–0.29; ACC: *g* = 0.14, 95% CI: 0.04–0.24). Further moderator analyses indicated that (a) for *language tasks*, TMS induced significant neuromodulatory effects on semantic and phonological tasks, but didn’t show significance for syntactic tasks; (b) for *cortical targets*, TMS effects were not significant in left frontal, temporal or parietal regions, but were marginally significant in the inferior frontal gyrus in a finer-scale analysis; (c) for *stimulation parameters*, stimulation sites extracted from previous studies, rTMS, and intensities calibrated to the individual resting motor threshold are more prone to induce robust TMS effects. As for stimulation frequencies and timing, both high and low frequencies, online and offline stimulation elicited significant effects; (d) for *experimental designs*, studies adopting sham TMS or no TMS as the control condition and within-subject design obtained more significant effects.

**Discussion:**

Overall, the results show that TMS may robustly modulate healthy adults’ language performance and scrutinize the brain-and-language relation in a profound fashion. However, due to limited sample size and constraints in the current meta-analysis approach, analyses at a more comprehensive level were not conducted and results need to be confirmed by future studies.

**Systematic review registration:**

[https://www.crd.york.ac.uk/PROSPERO/display_record.php?RecordID=366481], identifier [CRD42022366481].

## Introduction

Human language performance, including both comprehension and production abilities, is proposed to be a milestone of human evolution, distinct from any other animals (e.g., Friederici et al., 2017; Tattersall, 2017; Fishbein et al., 2020; Torday, 2021). Numerous studies have intensively investigated the neurobiology of human language performance through neuroimaging techniques such as electroencephalography (EEG), functional magnetic resonance imaging (fMRI), functional near-infrared spectroscopy (fNIRS), and magnetoencephalography (MEG) (e.g., Soltanlou et al., 2018; Tian et al., 2020; Wang et al., 2020; see also Friederici et al., 2017 for a systematic review). Nevertheless, these correlative neuroimaging approaches seem to be insufficient to interpret causal relationships between neural correlates and language functions (Devlin and Watkins, 2007; Sandrini et al., 2011; Hartwigsen, 2015). Clinical studies on post-stroke aphasia have evidenced such causality *via* mapping specific deficits after circumscribed lesions to language areas (Fridriksson et al., 2015; Wilson and Hula, 2019). However, patients’ language performance might be confounded by the long-term reorganization of brain networks, and broad lesions also brings difficulties to precise localization of language regions (Krieger-Redwood et al., 2013; Klaus et al., 2020). To overcome these problems, transcranial magnetic stimulation (TMS) was introduced as a focal method for cognitive neuroscience to probe the causal neural mechanisms for language in a non-invasive fashion (Flöel, 2012; Papeo et al., 2013; Hartwigsen, 2015; Uddén et al., 2017; Kennedy-Higgins et al., 2020; Kuhnke et al., 2020). In brief, the current in the TMS coil induces a perpendicular magnetic field which penetrates the scalp without attenuation to reach the stimulated brain region, and this magnetic field in turn induces a short-lived current that leads to stimulation of the neurons within the target region, resulting in either a facilitation or an inhibition effect as reflected by behavioral performance changes (Klomjai et al., 2015; Pitcher et al., 2020). Notably, these effects are collectively referred to as “neuromodulatory effects” in the current study.

Initially introduced for the stimulation of human motor cortex [Barker et al., 1985, as cited in Hartwigsen (2015)], TMS has been widely applied in clinical studies on language performance in recent years, including the identification of language lateralization as well as the localization of language-related brain regions before surgical resection in tumor patients (Han et al., 2021; Nettekoven et al., 2021), and the rehabilitation or treatment of language impairments such as post-stroke aphasia (Murdoch and Barwood, 2013). To guide such clinical applications and provide insight into the functional relevance of the language network, TMS studies in the healthy population are essential. The impact of TMS studies in healthy volunteers is twofold: On the one hand, causal mapping of structure-function relationships *via* TMS might deepen our understanding of the neurobiological mechanisms underlying normal language performance (Devlin and Watkins, 2007; Flöel, 2012); on the other hand, healthy participants with smaller variance might serve as an ideal model for the assessment of comparatively stable brain plasticity and compensatory effects (Hartwigsen et al., 2013; Jung and Lambon Ralph, 2016).

However, TMS effects are prone to be moderated by various factors such as tasks, target brain regions, brain state before and during stimulation, and stimulation parameters (Vallar and Bolognini, 2011; Hartwigsen, 2015; Hartwigsen and Silvanto, 2022), rendering the between-study heterogeneity inflated. For instance, with regard to specific language functions, TMS could reduce the accuracy (ACC) and prolong the reaction time (RT) for semantic tasks like picture naming and synonym judgment tasks (Whitney et al., 2012; Krieger-Redwood and Jefferies, 2014; Papeo et al., 2015; Klaus et al., 2020), whereas certain studies did not find any modulatory effects (Hartwigsen et al., 2010, 2016), or instead discovered reversed effects (Bonnì et al., 2015; Piai et al., 2020). This was also the case for syntactic and phonological tasks, in which the results were still disputed (Sakai et al., 2002; Uddén et al., 2008, 2017; Hartwigsen et al., 2010, 2016; Sliwinska et al., 2012; Acheson and Hagoort, 2013; Klaus and Hartwigsen, 2019; Deschamps et al., 2020; Ishkhanyan et al., 2020). Moreover, TMS effects also varied for different cortical regions (Devlin et al., 2003; Gough et al., 2005; Bonnì et al., 2015; Klaus and Hartwigsen, 2019). Since language areas are widely distributed across the frontal, temporal and parietal lobes in the left hemisphere, and within each lobe, a functional gradient for phonological, syntactic and semantic processes has been assumed (Xiang et al., 2010; Hagoort, 2013; Klaus and Hartwigsen, 2019), it becomes necessary to evaluate the efficacy of TMS on these three relatively large language-related lobes as well as more specific brain regions such as the inferior frontal gyrus (IFG), the superior temporal gyrus (STG), and the middle temporal gyrus (MTG). Therefore, the present study conducted moderator analyses on language tasks (of different language functions, including semantic, syntactic, and phonological tasks) as well as different cortical targets (i.e., brain regions of interest, including frontal, temporal and parietal lobes and IFG, STG and MTG).

In terms of TMS methodology, different TMS protocols were reported to be critical for influencing TMS effects (Klomjai et al., 2015; Sollmann et al., 2015; Sondergaard et al., 2021; Hartwigsen and Silvanto, 2022). Such parameters mainly involve (but are not limited to):

(a) Methods of localization (i.e., to choose an appropriate stimulation site for a target region through referring to previous studies or conducting localization by the current study *per se*) (Sparing et al., 2008; Sack et al., 2009; Flöel, 2012; Papeo et al., 2015; Jost et al., 2020);
(b) Stimulation types [differentiated according to the frequency and duration of magnetic pulses. Please note that in this study, TMS serves as a broad technical term for different specific stimulation types, containing: (i) repetitive TMS, consisting of trains of regularly repeated pulses of very short duration (milliseconds), including navigated rTMS, a combination of MR-based neuro-navigation systems and rTMS; (ii) theta burst stimulation (TBS), also known as patterned rTMS, characterized by the application of patterned bursts with short intervals of no stimulation, including intermittent TBS (iTBS) and continuous TBS (cTBS)] (Franzmeier et al., 2012; Schuhmann et al., 2012; Hartwigsen et al., 2013; Murdoch and Barwood, 2013; Jung and Lambon Ralph, 2016; León Ruiz et al., 2018; Deschamps et al., 2020);
(c) Timing (online and offline stimulation) (Hartwigsen, 2015; Sliwinska et al., 2017);
(d) Frequencies [high (>1 Hz) and low (≤1 Hz) frequency] (Bailey et al., 2001; Fitzgerald et al., 2006; Murdoch and Barwood, 2013; Hartwigsen, 2015; Beynel et al., 2019);
(e) Intensities [usually calibrated according to the resting motor threshold (RMT) or active motor threshold (AMT)] (Bailey et al., 2001; Sparing et al., 2001; Siebner et al., 2009).

Studies with various settings regarding these parameters have yielded mixed and unstable neuromodulatory effects on language performance. For example, studies investigating phonological processing found that high or low frequencies could lead to either enhancement or impairment of task performance (Andoh et al., 2006; Romero et al., 2006; Klaus and Hartwigsen, 2019). Similarly, stimulation intensities above or below the RMT might modulate the semantic response speed, but might also leave it unaffected (Hartwigsen et al., 2010; Krieger-Redwood and Jefferies, 2014; Bonnì et al., 2015). Therefore, for each parameter, the TMS effect was evaluated to identify optimal protocols for future TMS studies on language processing in healthy volunteers.

The specific experimental design is another potential factor contributing to the inconsistency among the related TMS studies’ findings. For example, regarding the control conditions, effective (active) stimulation of control sites, that is, unrelated brain regions (such as the vertex and other brain regions considered as irrelevant to the language functions under investigation), was claimed to be better than merely using sham stimulation (e.g., changing the angle of the stimulation coil with the target region un-stimulated) (Hartwigsen, 2015; Beynel et al., 2019). Nevertheless, the possibility that effective stimulation on the control sites could also interfere with task performance cannot be completely ruled out (Sandrini et al., 2011). Some studies, for instance, found that TMS perturbation of the occipital control sites affected semantic processing (Pobric et al., 2010; Papeo et al., 2015). There are also studies utilizing the comparison between different experimental tasks or language processing phases as the control condition to observe TMS effects (referred to as “others” in the current study), of which the effectiveness still awaits confirmation. As for the group designs, relative to between-subject designs, within-subject designs may reduce the individual variance for different conditions, but might suffer from repetition effects (i.e., improved task performance through repetitive presentation of certain materials), carry-over effects (i.e., effects carried over from one experimental treatment to another), or practice effects (i.e., improved task performance simply due to practice), and have to be implemented with a sufficiently long inter-session interval (usually about 1 week) for respective conditions (Hartwigsen, 2015). Therefore, it is still uncertain which control conditions or designs could elicit more robust TMS effects on language performance.

The latest search shows there have been only three meta-analyses addressing TMS effects on healthy participants’ language performance (Klaus and Schutter, 2018; Beynel et al., 2019; Johnson, 2021), but none of them took TMS or language comprehension as well as production abilities as their primary focus. Therefore, this study aims to systematically evaluate the efficacy of neuromodulatory TMS effects on language performance in healthy participants by meta-analyzing (a) the overall TMS effects on healthy participants’ language task performance, and (b) zooming in on the main moderators of TMS effects in previous TMS language performance-related studies — language tasks, cortical targets, stimulation parameters, and experimental designs. We expect the findings of this meta-analysis to (a) enrich the results found by previous TMS meta-analyses on aphasics (Ren et al., 2014; Bucur and Papagno, 2019; Hong et al., 2021; Zhang et al., 2021), (b) clarify the efficacy of TMS effects on healthy participants’ language performance, including both comprehension and production abilities (cf. Klaus and Schutter, 2018; Beynel et al., 2019), and (c) inform future TMS studies in the neurolinguistic field with respect to optimized designs and parameters.

## Materials and methods

This study followed the PRISMA (The Preferred Reporting Items for Systematic reviews and Meta-Analyses) guidelines (Page et al., 2021) and has been registered in the international prospective register of systematic reviews (PROSPERO) under the registration number: CRD42022366481.

### Study search and selection

We conducted a literature search in *PubMed, Web of Science, PsycINFO, Scopus*, and *Google Scholar* from the inception of the databases to October 15th, 2022 to identify eligible studies by querying all TMS studies (including original studies, reviews, and meta-analyses) on language comprehension and production in healthy participants, published in English. The search terms were any combination of (“Language, Syntax, Grammar, Semantics, Meaning, Phonetics, OR Phonology”) AND (“TMS” OR “transcranial magnetic stimulation”). The reference lists of previous reviews and meta-analyses (Vigneau et al., 2006; Flöel, 2012; Ren et al., 2014; Otal et al., 2015; Klaus and Schutter, 2018; Beynel et al., 2019; Bucur and Papagno, 2019; Hartwigsen and Saur, 2019; Hong et al., 2021; Zhang et al., 2021) were also screened in case any related studies were overlooked. The eligible study selection criteria are listed as following:

(a) Participants were healthy adults (aged between 18 and 60 years old) (Gingras et al., 2021), right-handed, and for each experiment, the sample size was ≥5 (participants). Since children’s and teenagers’ brains are still developing, and aging brains (>60 years old) may confront both structural and functional decrease, only healthy adults within the specified age range were deemed an ideal and comparatively steady target population for this study. Therefore, studies reporting juvenile or aging participants’ data were excluded (Taylor and Burke, 2002; Wierenga et al., 2014; Jernigan et al., 2016).
(b) Transcranial magnetic stimulation was applied to the cerebral cortex of the participants. Given that the relationship between the cerebellum and language functions is still largely unclear, studies applying TMS over the cerebellum were tentatively excluded (e.g., Argyropoulos and Muggleton, 2013; de Smet et al., 2013). Moreover, several navigated TMS (nTMS) studies mapping language functions (esp., word production *via* picture-naming) were also excluded because there was no explicit baseline. Also, since such nTMS studies aimed to map all possible brain regions involved in language processing (i.e., brain-language mapping), they focused on the relative rather than the absolute task sensitivity differences among the brain regions (e.g., Krieg et al., 2016; Tussis et al., 2017).
(c) Research contents included language comprehension and/or production tasks. Note that certain studies using language materials to investigate general cognitive abilities such as memory, attention, and reasoning (e.g., Innocenti et al., 2013; Möttönen et al., 2014), and studies focusing on sign languages (e.g., Banaszkiewicz et al., 2021), low-level orthographic processing (e.g., Lavidor and Walsh, 2003; Pattamadilok et al., 2010) as well as extended meaning comprehension and discourse reading engaging advanced language processing (e.g., Pobric et al., 2008; Tomasino et al., 2008; Cacciari et al., 2011; Gough et al., 2013) were excluded.
(d) Means and standard deviations of reaction time (RT) and/or accuracy (ACC) were reported or obtained upon authors’ requests.
(e) The trials were controlled and randomized.
(f) Studies were formally published in international peer-reviewed English journals and were officially approved by medical ethical committees or review boards.

**FIGURE 1:**
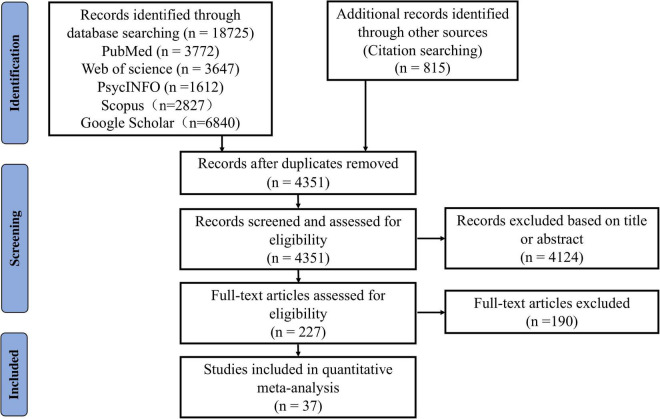
The Preferred Reporting Items for Systematic reviews and Meta-Analyses (PRISMA) flow diagram of literature search and selection for the meta-analysis.

Each study underwent three screening steps for final inclusion: (1) Removal of duplicates (this was done by XQ, ZW, YC, QX, ZL, and LL); (2) Screening of titles and abstracts (XQ and ZW first independently evaluated all the studies and then conducted a check together. Studies met with disagreement were entered into the third step); (3) Full-text review (by XQ and ZW). Disagreement was resolved through group discussions among all authors. In addition, we also calculated Cohen’s kappa coefficient to assess inter-rater reliability (*k* = 0.803, *p* < 0.001), obtaining strong consistency (McHugh, 2012). The procedures of the study search and selection are depicted in Figure 1.

### Data extraction

Thirty-seven eligible studies were identified. The means and standard deviations of RT and ACC, and the sample sizes were extracted. For each study, the following information was collected: literature information (authors and publication year), participant characteristics (sample size, gender, age, and native language), tasks, cortical targets, TMS protocols (methods of localization, stimulation types, timing, frequencies, and intensities), and study designs (control conditions and group designs). Table 1 provides an overview of the included studies.

**TABLE 1:** Overview of the studies included in the meta-analysis.

References	Sample size	Age	First language	Tasks	Cortical targets	TMS protocols (methods of localization, types, timing, frequencies, intensities)	Control conditions	Group designs
Sakai et al., 2002	6	22–49	Japanese	Syntactic decision task Semantic decision task	Left IFG, Left MFG	Current study, event-related TMS, online, 55–98% AMT	Sham TMS	Within-subject
Finocchiaro et al., 2008	11	22–38	Italian	Verb phrase production task Picture naming task	Left prefrontal cortex	Previous study, rTMS, offline, 1 Hz, 100% RMT	Sham TMS, Other brain regions	Mixed
Schuhmann et al., 2009	12	20–26	Dutch	Picture naming task	Broca’s area	Previous study, tpTMS, online, 40 Hz, 120% RMT	Sham TMS	Within-subject
Hartwigsen et al., 2010	35	Exp, 20–28 (1), 20–30 (2) Ctr, 21–27	German	Word judgments task	Left plFG, left alFG	Previous study, rTMS, online, 10 Hz, 90% AMT	Sham TMS	Between-subject
Andoh and Paus, 2011	10	21.8 (SD = 4.3)	English	Word recognition task	Left and right TMP	Previous study, rTMS, offline, 10 Hz, 45–85% RMT	No TMS	Within-subject
Willems et al., 2011	20	19–35	/	Lexical decision task	Left and right PC	Previous study, TBS, offline, 5 Hz, 80% MT	No TMS	Mixed
Franzmeier et al., 2012	59	21.7 (SD = 3.4)	English	Plausibility judgment task	Left and right MTL, Left and right STS	Current study, single pulse TMS, online, 90% MT	Vertex	Within-subject
Schuhmann et al., 2012	8	20–26	Dutch	Picture naming task	IFG, MTG, STG	Previous study, nTMS, online, 40 Hz, 120% RMT	No TMS	Within-subject
Whitney et al., 2012	16	22.25 (SD = 3.55)	English	Semantic judgment task	Left IFG, Left pMTG, left parietal lobe	Previous study, rTMS, offline, 1 Hz, 120% AMT	No TMS	Within-subject
	16	23.3 (SD = 4)	English	Phonological discrimination task	Left and right SMG	Previous study, rTMS, offline, 1 Hz, 110% RMT	Sham TMS	Within-subject
Hartwigsen et al., 2013	17	23.8 (SD = 2.2)	German	Pseudowords/words repetition task	Left IFG	Previous study, cTBS, offline, 50 Hz, 80% AMT	Sham TMS	Between-subject
Krieger-Redwood et al., 2013; Deschamps et al., 2014	14	21.8 (SD = 2.4)	English	Phoneme judgment task Semantic category judgment task Non-linguistic control task	Left PMC, left pSTG, Left occipital pole	Previous study, rTMS, offline, 1 Hz, 120% MT	Other brain regions	Within-subject
Krieger-Redwood and Jefferies, 2014	18	20.78 (SD = 2.37)	English	Cyclical naming task	Left IFG, pMTG	Previous study, rTMS, offline, 1 Hz, 120% MT	No TMS	Within-subject
Bonnì et al., 2015	18	24.9 (SD = 2.5)	Null	Semantic association task	Left and right ATL	Previous study, cTBS, offline, 50 Hz, 80% AMT	Vertex	Within-subject
Davey et al., 2015	15	23	English	Word-to-picture matching task	Left pMTG, left ANG	Previous study, rTMS, offline, 1 Hz, 120% RMT	Vertex No TMS others	Within-subject
Finocchiaro et al., 2015	30	Exp, 19–37 Ctr, 19–36	Italian	Word-picture matching task Sentence-picture matching task	Left parietal lobe	Current study, rTMS, online, 5 Hz, 90% RMT	No TMS	Mixed
Jackson et al., 2015	15	24.39 (SD = 5.98)	English	Synonym judgment task Number judgment task	Left ATL	Current study, rTMS, online, 100% MT	Others	Within-subject
Karabanov et al., 2015	26	Exp, 26.6 (SD = 6.3) (1) Exp, 30.6 (SD = 9.2) (2)	English	Auditory memory task	Left pSTG, left IFG	Previous study, rTMS, online, 10 Hz, 110% RMT	Other brain regions	Mixed
Passeri et al., 2015	18	19–27	Italian	Verbal category membership task	Wernicke’s Area and its right homolog	Previous study, rTMS, online, 10 Hz, 100% MT	Sham TMS	Within-subject
Papeo et al., 2015	14	19–35	Italian	Synonym judgment task	Left pMTG	Previous study, rTMS, offline, 1 Hz, 65% maximum stimulator output	No TMS, Other brain regions	Within-subject
Hartwigsen et al., 2016, Exp1	17	23–30	German	Word syllable categorization task Word semantic categorization task	Left ANG, left SMG, Left aIFG, left pIFG	Previous study, cTBS, offline, 50 Hz, 80% RMT Previous study, rTMS, online, 10 Hz, 90% RMT	Sham TMS	Within-subject
Hartwigsen et al., 2016, Exp2	17	20–30	German	Word syllable categorization task Word semantic categorization task	Left ANG, left SMG, Left aIFG, Left pIFG	Previous study, cTBS, offline, 50 Hz, 80% RMT Previous study, rTMS, online, 10 Hz, 90% RMT	Others	Within-subject
Uddén et al., 2017	13	24 (SD = 3)	Dutch	Grammaticality classification task	Left IFG	Previous study, rTMS, offline, 1 Hz, 110% RMT	Vertex	Within-subject
Woollams et al., 2017	18	22 (SD = 3) 21.89 (SD = 3.3)	English	Word-reading aloud task	Left ATL	Previous study, rTMS, offline, 1 Hz, 120% MT	No TMS	Mixed
Klaus and Schutter, 2018	24	20–34	German	Semantic production task Phonological production task	Left IFG, Left pIFG	Previous study, rTMS, online, 10 Hz, 38.0%(*M*) maximum stimulator output	Vertex	Within-subject
Zhang et al., 2018	21	23.7 (SD = 1.35)	Chinese	Picture naming task	Broca’s area	Previous study, tpTMS, online, 40 Hz, 100% MT	Sham TMS	Within-subject
Klaus and Hartwigsen, 2019	16	23	Dutch	Context-driven picture naming task	Left MTG	Previous study, cTBS, offline, 50 Hz, 80% RMT	Sham TMS	Within-subject
Deschamps et al., 2020	18	25.2 (SD = 3.91)	French	Delayed auditory discrimination task	Left pIFG, Left aSMG	Current study, single pulse TMS, online, 110% RMT	No TMS	Within-subject
Ishkhanyan et al., 2020	19	18–34	Danish	Adjective-noun production task	Left aIFG, Left pIFG	Previous study, rTMS, online, 10 Hz, 110% RMT	Sham TMS	Within-subject
Jost et al., 2020	22	22.4 (SD = 2.2)	French	Picture naming task Word translation task Flanker task	Left DLPFC	Current study, cTBS, offline, 30 Hz, 80% RMT	Sham TMS	Within-subject
Piai et al., 2020	24	21–35	German	Picture-word interference task	Left pSTG	Previous study, rTMS, online, 10 Hz, 90% RMT	Vertex	Within-subject
Finocchiaro et al., 2021	16	19–35	Italian	Argument judgment task	Left PPS	Previous study, double pulse TMS, online, 100% MT	Sham TMS	Within-subject
Ohlerth et al., 2021	18	20–53	German	Object naming task Action naming task	Whole brain (46 sites)	Previous study, nTMS, online, 5 Hz, 110% RMT	No TMS	Within-subject
Sliwinska et al., 2021	20	19–25	English	Novel vocabulary training task Novel vocabulary matching task	Left and right parietal, Left and right precentral	Previous study, cTBS, offline, 30 Hz, 45% MT	Other brain regions	Within-subject
Smalle et al., 2022	36	Exp, 25.3 (SD = 4.8) Ctr, 23.4 (SD = 5.0)	English	Auditory forced-choice recognition task	Left DLPFC	Current study, cTBS, offline, 30 Hz, 80% AMT	Vertex	Between-subject
van der Burght et al., 2022	30	19–37	German	Sentence completion task	Left pIFG, left aIFG	Previous study, rTMS, online, 10 Hz, 90% RMT	Vertex	Within-subject
Vitale et al., 2022	64	Exp, 23.5 (SD = 4.0) Ctr, 20.6 (SD = 2.7)	Spanish	Sentence semantic judgment task	Right IFG	Previous study, rTMS, online, 1 Hz, 90% RMT	Sham TMS	Between-subject
Ward et al., 2022	20	18–44	English	Picture naming task	Left PMv, Left IPS	Previous study, rTMS, online, 1 Hz, 71% to 100% RMT	Sham TMS, Other brain regions	Within-subject

IFG, inferior frontal gyrus; MFG, middle frontal gyrus; PC, premotor cortex; TMP, posterior temporal area of Wernicke; MTL, medial temporal lobe; STS, superior temporal sulcus; MTG, middle temporal gyrus; STG, superior temporal gyrus; PMC, primary motor cortex; SMG, supramarginal gyrus; ATL, anterior temporal lobes; ANG, angular gyrus; DLPFC, dorsolateral prefrontal cortex; PPS, posterior parietal sulcus; PMv, ventral premotor cortex; IPS, intra-parietal sulcus; rTMS, repetitive transcranial magnetic stimulation; tpTMS, three pulses transcranial magnetic stimulation; nTMS, navigated transcranial magnetic stimulation; cTBS, continuous theta-burst stimulation; MT, motor threshold; RMT, resting motor threshold; AMT, active motor threshold. Sample size refers to the number of subjects. “Current study” indicates that the stimulation sites were determined by the study itself. By that analogy, “previous study” means that the coordinates were extracted from previous literature. The “other brain region” in the control conditions refers to the right homologous or the irrelevant brain regions as the control sites. The “others” refers to comparisons between different experimental tasks or language processing phases.

XQ, ZW, YC, QX, ZL, and LL independently extracted data from each study. XQ and ZW further checked the extracted data together. Missing data pertinent to the current study were obtained and authorized by e-mailing the authors. The extracted data were recorded in excel spreadsheets. Data extraction and management were conducted manually.

### Data analyses

We used mean and standard deviation to calculate Hedges’*g* (abbreviated as “*g*” hereafter) to estimate effect size, which provides a less biased estimate of the true effect than Cohens’*d*, especially for studies with small sample sizes (Hedges and Olkin, 1985). In line with Klaus and Schutter (2018), this meta-analysis mainly focused on the absolute effect sizes, that is, the magnitude of the neuromodulatory TMS effects regardless of the effect directions (i.e., improvement of behavioral performance as facilitation, and disruption of behavioral performance as inhibition). We performed data aggregation to avoid multiple similar data points from a single experiment entering the analysis, ensuring that each experiment provided only one measured value (Borenstein et al., 2009). Nevertheless, if several conditions were tested for more elaborate comparisons of the experimental variables within one experiment, the effect sizes would be calculated separately for each comparison (see also Klaus and Schutter, 2018). For example, if a study compared the conditions within various brain regions, for each brain region, the effect sizes were aggregated as one and included in the meta-analysis.

Since the included studies encompassed within-subject designs, it was difficult to assume statistical independence in the meta-analysis, and the issue of effect size dependency should be considered (Hunter and Schmidt, 2004). In previous studies, there were five commonly used methods to deal with correlated effect size: (a) Ignore dependencies; (b) Average dependent effect sizes across studies; (c) Extract only one effect size from each study; (d) Introduce the correlation coefficient *r*. However, these methods are suggested to be confronted with problems such as the exaggeration of relevant significance tests (Cheung, 2014), the lowering of the statistical power of meta-analysis and the precision of parameter estimation (Van den Noortgate et al., 2013) and too conservative estimation of coefficient value (Hedges and Pigott, 2001; Cheung, 2014). The fifth method (e) adopts modeling, such as the frequently used multilevel random effects model. Compared to the aforementioned four methods, using multilevel random effects model to estimate the effect sizes in meta-analysis is more accurate, effective and flexible. The model can incorporate multiple predictors to account for heterogeneity between studies or add additional random effects to address the various dependencies of effect sizes within and between studies (Fernández-Castilla et al., 2020). Therefore, to solve the problem of effect size dependency especially for within-subject designs, a three-level random effects model was adopted for the meta-analysis in this study (see Supplementary material for more details regarding the five methods and related rationales).

We also conducted the likelihood ratio test (LRT) to explore whether the three-level random effects model was more suitable for this meta-analysis compared to the traditional two-level random effects model. There was a significant difference between the traditional random effects model and the three-level random effects model in ACC (LRT = 9.99, *p* = 0.007), and a trend toward significance in RT (LRT = 2.80, *p* = 0.09). This indicated that compared with the traditional random effects model, the three-level model provided a better model fit.

Study heterogeneity was estimated by Cochran’s *Q* and *p*-value (Huedo-Medina et al., 2006), and all effect sizes were entered into the three-level random effects model. We also conducted sensitivity analysis to verify the robustness of the results.

According to the Cochrane guideline (Higgins et al., 2019), we used the Cochrane risk of bias tool (RoB 2.0, Sterne et al., 2019) to evaluate the quality of the included studies in the following aspects: (a) randomization process; (b) deviations from intended interventions; (c) missing outcome data; (d) measurement of the outcome; (e) selection of the reported result. Two researchers assessed the quality of each study. Studies met with disagreement were evaluated through pairing with a third party for group assessment.

Moreover, to account for the fact that the overall effect size might be overestimated because of publication bias (Rothstein and Bushman, 2012), we tested for a potential publication bias by constructing a “funnel plot”. If there was no bias, the graph would present an inverted funnel shape, and the distribution of the points (i.e., the included studies) would be roughly symmetrical. In case of a publication bias, the funnel would be skewed (Van den Bussche et al., 2009). However, concerns about the subjectivity of funnel plots have been raised. To ensure reliability, we further quantified publication bias by the Egger Test (Egger et al., 1997).

**FIGURE 2:**
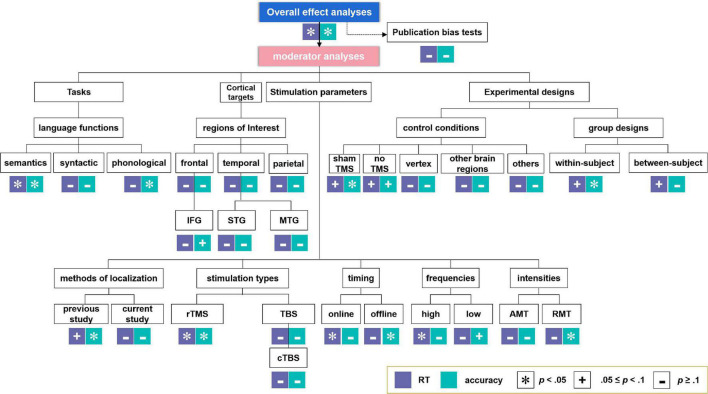
Analysis-structure and results for overall effect analyses and moderator analyses. *Indicates significant effects. ^+^Indicates marginally significant effects. ^–^Indicates non-significant effects.

Moderator analyses were performed, since experimental parameters such as the language tasks (especially for different language functions, including semantic, syntactic, and phonological tasks), cortical targets [i.e., brain regions of interest, containing frontal, temporal, and parietal regions, as well as more specific regions including IFG, STG, and MTG. Please note that owing to the lack of adequate sample sizes, we did not perform meta-analysis on other specific regions such as the premotor cortex (PC), the anterior temporal lobe (ATL) and the angular gyrus (AG)], stimulation parameters of the TMS protocols [i.e., methods of brain region localization, stimulation types (further analysis on cTBS but not iTBS (only one study) was conducted considering the inadequate sample sizes), timing, frequencies, and intensities], and experimental designs (i.e., control conditions and group design types) are crucial for the examination of TMS effects on language performance in healthy participants. Effect sizes with an associated 95% confidence intervals (CI) were calculated when at least two studies were available for a particular estimate (see also Valentine et al., 2010). The analysis-structure of both the overall and the moderator analyses are summarized in Figure 2.

All effect size computations, summary analyses, sensitivity analysis, risk of bias tests and the publication bias tests were conducted by using the “metafor 3.0-2” (Viechtbauer, 2010) implemented in R (version 4.0.4, R Core Team, 2021). The results of all effect analyses of RT and ACC were graphically synthesized in forest plots.

## Results

### Overall transcranial magnetic stimulation effects

**FIGURE 3:**
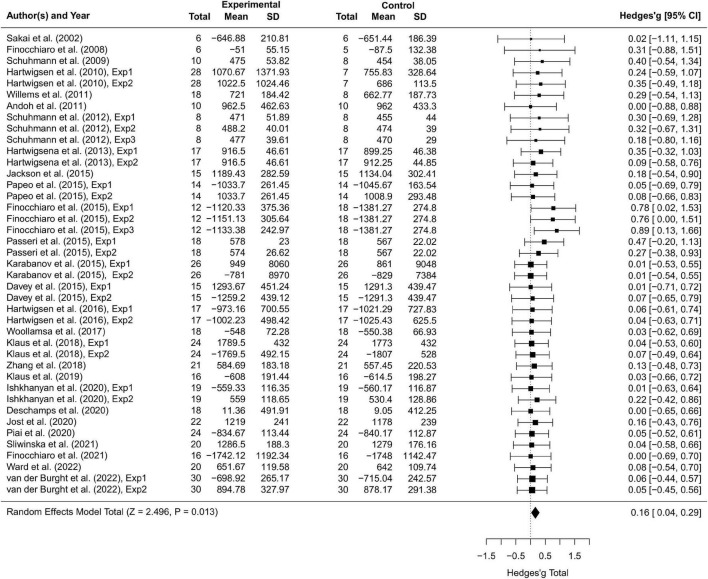
Forest plot of reaction times (RTs) of overall effect analysis. The discrepancy in sample size between the experimental group and the control group in some studies is due to the between-subject experimental design.

**FIGURE 4:**
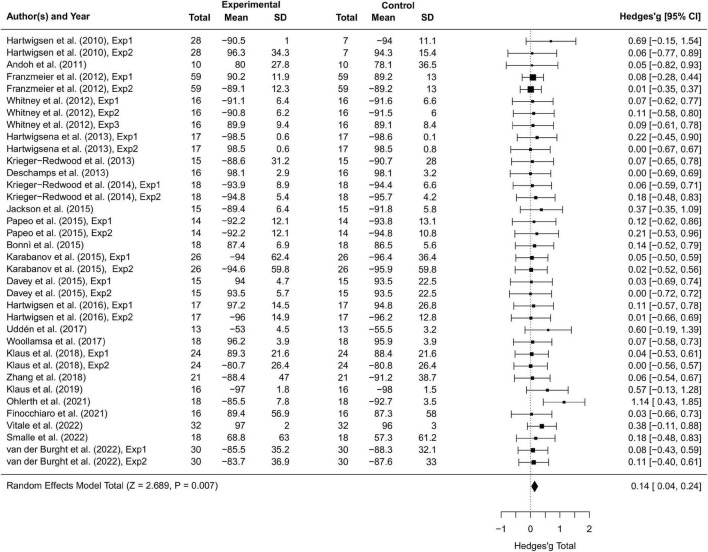
Forest plot of accuracy (ACC) of overall effect analysis.

Thirty-seven studies including 797 participants and 77 effect sizes were computed for the overall effect analysis. The results (see also Figures 3, 4) showed that for both RT and ACC, TMS could exert significant neuromodulatory effects on language performance in healthy participants (RT: *g* = 0.16, 95% CI: 0.04–0.29, *Z* = 2.496, *p* = 0.013; ACC: *g* = 0.14, 95% CI: 0.04–0.24, *Z* = 2.689, *p* = 0.007). The sensitivity analysis revealed that the results were still significant after removing the studies with the maximum and the minimum weight, which indicated the stability of RT and ACC in the overall analysis (see Table 2 for details).

**TABLE 2:** Reaction time (RT) and accuracy (ACC) results of sensitivity analyses.

	Method	Study	Model	*g*, CI	*P*_1_	*P*_2_
RT	Remove the maximum weighted study	van der Burght et al., 2022	Multilevel random effects model	0.17, 0.04–0.29	0.012	0.99
	Remove the minimum weighted study	Finocchiaro et al., 2008	Multilevel random effects model	0.16, 0.03–0.29	0.017	0.99
ACC	Remove the maximum weighted study	Franzmeier et al., 2012	Multilevel random effects model	0.15, 0.05–0.26	0.005	0.99
	Remove the minimum weighted study	Andoh and Paus, 2011	Multilevel random effects model	0.14, 0.04–0.25	0.007	0.99

*P*_1_ calculated the significance of effect size, and *P*_2_ calculated the significance of heterogeneity.

**FIGURE 5:**
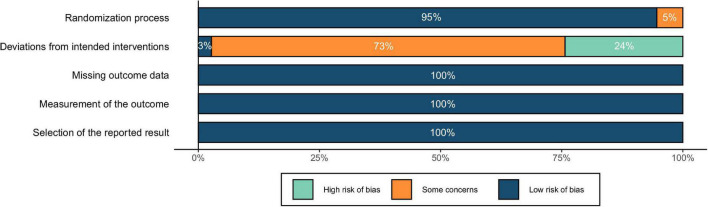
Assessment of risk of bias for included studies.

The risk of bias was depicted in Figure 5. The results showed a low risk of bias in terms of randomization process. As for the risk of deviations from intended interventions (i.e., effects of blindness), all studies were single blind except for one double-blind study. Regarding the blinding of participants, studies using “vertex,” “other brain regions,” and “others” as the control conditions simulated the auditory and somatosensory sensations caused by the active stimulation, resulting in a low risk of bias. With “sham TMS,” although the auditory sensations could be similar to that of active stimulation, the somatosensory sensations were different, which might lead to mild concerns of blindness. In comparison, the control condition of “no TMS” could be easily differentiated from the active stimulation by the participants, resulting in potential high risk of bias. Finally, for the risk of missing the outcome data, inaccurate measurement of the outcomes, and reporting selected results, no evidence indicating an unclear or high risk of bias was found in the included studies. To summarize, although there might be a few risks of blinding, the overall quality could still be acceptable, and no study was eliminated because of low quality.

**FIGURE 6:**
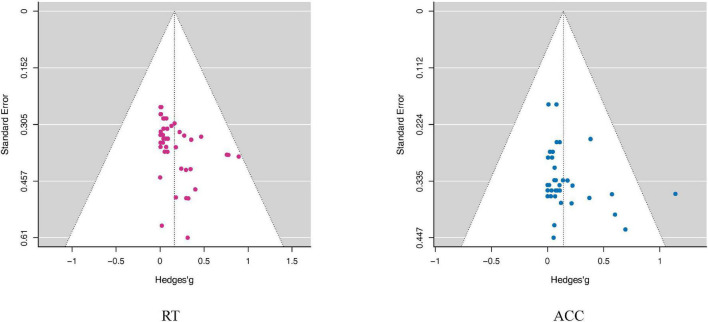
Funnel plots assess publication bias in the RT and ACC outcomes. The Funnel plots took the Mean difference as the abscissa and Standard Error as the ordinate. The small dots in the figure represent the included studies. In the funnel plot, the dotted line perpendicular to the horizontal axis represents the overall effect, and the dotted line on both sides represents the 95% confidence interval (CI). As shown in this figure, the distribution of all studies in the funnel plot was roughly symmetric, suggesting that there was no publication bias. Although the funnel plot can visually observe publication bias, it is subjective, and it can be seen that individual studies deviate from 95% confidence interval (CI). Therefore, we adopted Egger’s test to quantify the publication bias. Note that the point outside the white area in the ACC funnel plot does not represent an outlier, because the significance of the further quantitative Egger Test was not affected by including or excluding this study.

The publication bias tests showed no significant results in RT or ACC (RT: Egger: *p* = 0.69; ACC: Egger: *p* = 0.23), indicating that the overall effect sizes should not be enhanced by publication biases (see also Figure 6).

### Moderator analyses results

The moderator analyses results were summarized in Tables 3, 4 for RT and ACC, respectively, and displayed in Figure 2 (see Supplementary material for the forest plots, including moderator analyses and additional analyses).

**TABLE 3:** Reaction time (RT) results of overall effect analyses and moderator analyses.

Analysis type	Sub-category	*N*	*g*	*P*_1_	95% CI	*Q*	*P*_2_
Overall	Overall	27	0.16	0.013	0.04–0.29	13.829	0.99
Tasks (language functions)	Semantics	22	0.13	0.045	0.00–0.25	3.839	1.00
	Syntax	6	0.31	0.205	−0.17–0.78	7.742	0.46
	Phonological	12	0.12	0.139	−0.04–0.28	2.162	1.00
Regions of interest	Frontal	13	0.12	0.130	−0.04–0.28	2.208	1.00
	Temporal	9	0.12	0.296	−0.10–0.33	1.855	0.99
	Parietal	5	0.31	0.252	−0.22–0.84	7.690	0.26
	IFG	12	0.12	0.141	−0.04–0.28	2.086	0.99
	STG	3	0.05	0.793	−0.32–0.41	0.088	0.96
	MTG	3	0.10	0.628	−0.29–0.48	0.248	0.97
Methods of localization	Previous study	21	0.12	0.063	−0.01–0.24	4.486	1.00
	Current study	6	0.24	0.144	−0.08–0.56	8.631	0.37
Stimulation types	rTMS	20	0.18	0.022	0.03–0.33	12.701	0.99
	TBS	6	0.14	0.276	−0.11–0.39	0.772	0.99
	cTBS	5	0.13	0.356	−0.14–0.39	0.629	0.99
Timing	Online	16	0.19	0.032	0.02–0.36	12.353	0.98
	Offline	12	0.10	0.251	−0.07–0.28	1.259	1.00
Frequencies	High	19	0.21	0.019	0.03–0.38	12.728	0.99
	Low	5	0.08	0.566	−0.20–0.37	0.288	1.00
Intensities	AMT	2	0.25	0.188	−0.12–0.62	0.363	0.95
	RMT	12	0.20	0.108	−0.04–0.45	10.807	0.93
Control conditions	Sham TMS	12	0.17	0.069	−0.01–0.35	2.263	0.99
	No TMS	5	0.31	0.097	−0.06–0.67	6.491	0.59
	Vertex	5	0.11	0.283	−0.09–0.31	1.665	0.99
	Other brain regions	4	0.04	0.764	−0.25–0.53	0.240	0.99
	Others	5	0.15	0.306	−0.14–0.43	0.794	0.97
Group designs	Within subject	18	0.11	0.065	−0.01–0.24	3.509	1.00
	Between subject	7	0.27	0.060	−0.01–0.55	8.761	0.64

*P*_1_ calculated the significance of effect size, and *P*_2_ calculated the significance of heterogeneity.

**TABLE 4:** Accuracy (ACC) results of overall effect analyses and moderator analyses.

Analysis type	Sub-category	*N*	*g*	*P*_1_	95% CI	*Q*	*P*_2_
Overall	Overall	24	0.14	0.007	0.04–0.24	15.992	0.99
Tasks (language functions)	Semantics	20	0.14	0.012	0.03–0.25	14.231	1.00
	Syntax	3	0.18	0.334	−0.18–0.54	1.412	0.49
	Phonological	12	0.16	0.045	0.00–0.32	12.038	0.741
Regions of interest	Frontal	10	0.12	0.150	−0.04–0.29	3.925	0.99
	Temporal	6	0.08	0.519	−0.16–0.31	0.072	1.00
	Parietal	4	0.09	0.598	−0.25–0.44	0.050	0.99
	IFG	11	0.14	0.075	−0.01–0.30	4.905	0.99
	STG	2	0.06	0.791	−0.49–0.37	0.006	0.94
	MTG	3	0.26	0.163	−0.10–0.62	1.088	0.78
Methods of localization	Previous study	19	0.14	0.030	0.01–0.26	7.006	1.00
	Current study	5	0.27	0.131	−0.08–0.62	8.993	0.17
Stimulation types	rTMS	18	0.15	0.014	0.03–0.28	13.550	0.98
	TBS	5	0.20	0.162	−0.08–0.47	1.258	0.91
	cTBS	5	0.20	0.162	−0.08–0.47	1.258	0.91
Timing	Online	9	0.10	0.187	−0.05–0.24	8.956	0.78
	Offline	16	0.16	0.025	0.02–0.31	5.048	0.99
Frequencies	High	12	0.11	0.161	−0.04–0.26	14.480	0.63
	Low	10	0.16	0.067	−0.01–0.34	3.217	1.00
Intensities	AMT	5	0.16	0.192	−0.08–0.39	1.969	0.98
	RMT	8	0.27	0.019	0.04–0.49	10.989	0.36
Control conditions	Sham TMS	7	0.24	0.050	0.00–0.47	3.739	0.81
	No TMS	7	0.20	0.087	−0.03–0.42	7.686	0.57
	Vertex	7	0.12	0.289	−0.10–0.33	1.849	0.99
	Other brain regions	3	0.07	0.602	−0.19–0.33	0.192	1.00
	Others	4	0.22	0.176	−0.10–0.53	1.841	0.77
Group designs	Within subject	16	0.19	0.011	0.04–0.35	19.797	0.76
	Between subject	6	0.17	0.135	−0.05–0.40	2.973	0.89

*P*_1_ calculated the significance of effect size, and *P*_2_ calculated the significance of heterogeneity.

#### Language tasks

For tasks concerning different language functions, TMS induced significant neuromodulatory effects for semantic tasks on both RT and ACC (RT: *g* = 0.13, 95% CI: 0.00–0.25, *p* = 0.045; ACC: *g* = 0.14, 95% CI: 0.03–0.25, *p* = 0.012). However, TMS showed no significant influence on syntactic tasks. For phonological tasks, TMS significantly affected ACC (*g* = 0.16, 95% CI: 0.00–0.32, *p* = 0.045).

#### Cortical targets

Transcranial magnetic stimulation did not show significant effects in larger frontal, temporal, or parietal regions. Further analysis on specific brain regions indicated that TMS had marginal significant effects on the IFG in ACC (*g* = 0.14, 95% CI: −0.01–0.30, *p* = 0.075).

#### Parameters of the stimulation protocols

The moderator analysis on the methods of localization indicated that TMS on the coordinates extracted from previous studies could exert significant effects on ACC (*g* = 0.14, 95% CI: 0.01–0.26, *p* = 0.030) and presented a trend for significance in RT (*g* = 0.12, 95% CI: −0.01–0.24, *p* = 0.063), whereas TMS on the sites detected by the studies *per se* elicited non-significant effects.

As for the stimulation types, rTMS had robust neuromodulatory effects on both RT and ACC (RT: *g* = 0.18, 95% CI: 0.03–0.33, *p* = 0.022; ACC: *g* = 0.15, 95% CI: 0.03–0.28, *p* = 0.014). Neither TBS nor cTBS showed significant effects on RT or ACC.

Tests on the timing parameters revealed that online stimulation could induce significant neuromodulatory effects on RT (*g* = 0.19, 95% CI: 0.02–0.36, *p* = 0.032), whereas offline TMS manifested significance in ACC (*g* = 0.16, 95% CI: 0.02–0.31, *p* = 0.025).

Regarding the stimulation frequencies, the moderator analysis with absolute effect sizes supported the notion that high-frequency TMS would significantly influence RT (*g* = 0.21, 95% CI: 0.03–0.38, *p* = 0.019), while low-frequency TMS showed a trend toward significantly affecting ACC (*g* = 0.16, 95% CI: −0.01–0.34, *p* = 0.067). Given that different frequency types were proposed to be related to different effect directions (at least in the motor system), that is, high-frequency TMS tends to have a facilitatory effect, whereas low-frequency TMS tends to have an inhibitory effect (Sandrini et al., 2011; Hartwigsen, 2015; Beynel et al., 2019), we ran an additional analysis with the original effect sizes. Although no significant effect was found, the results indicated that high-frequency TMS tended to reduce ACC instead of facilitating it (see Supplementary Figure 50).

Finally, the moderator analysis of TMS intensities revealed that RMT rather than AMT induced significant effects on ACC (*g* = 0.27, 95% CI: 0.04–0.49, *p* = 0.019).

#### Experimental designs

The moderator analysis on control conditions revealed that sham TMS displayed significance in ACC and was marginally significant in RT, suggesting that compared to other control conditions, this condition could serve as a promising baseline for detecting TMS effects (RT: *g* = 0.17, 95% CI: −0.01–0.35, *p* = 0.069; ACC: *g* = 0.24, 95% CI: 0.00–0.47, *p* = 0.050). Also, the “no TMS” control condition showed a trend of significance in RT and ACC (RT: *g* = 0.31, 95% CI: −0.06–0.67, *p* = 0.097; ACC: *g* = 0.20, 95% CI: −0.03–0.42, *p* = 0.087). The other conditions did not show any significant effects.

When taking the individual variance into consideration, within-subject designs seemed to be optimal for identifying significant TMS effects on ACC and showed marginally significant effects on RT (RT: *g* = 0.11, 95% CI: −0.01–0.24, *p* = 0.065; ACC: *g* = 0.19, 95% CI: 0.04–0.35, *p* = 0.011). Between-subject designs only exhibited TMS effects on RT in an approaching-significance way (*g* = 0.27, 95% CI: −0.01–0.55, *p* = 0.060).

## Discussion

By meta-analyzing the currently available data, this study aimed at evaluating neuromodulatory TMS effects on language performance in healthy adult volunteers. The overall effect analyses revealed that TMS significantly affected language task performance (as reflected by changes in RT and ACC), which was in line with the findings of previous studies (e.g., Klaus and Schutter, 2018; Beynel et al., 2019).

Although TMS seems to be a promising non-invasive technique for investigating causal structure-function relationship in language domain, both the stability and reliability still await to be assessed (Walsh and Cowey, 2000). Therefore, our subsequent moderator analyses further specified the efficacy of TMS on language performance regarding moderators in four critical aspects—language tasks, cortical targets, stimulation parameters, and experimental designs.

### Language tasks

Transcranial magnetic stimulation significantly modulated task performance for both semantic and phonological tasks, contrasting its non-significant effects on syntactic tasks.

For semantic tasks, TMS effects were manifested both on RT and ACC. These robust modulatory effects might be attributed to two reasons. First, semantic processing recruits broadly distributed but highly interactive regions in the left hemisphere such as the inferior frontal, and posterior temporo-parietal cortices (Hartwigsen et al., 2010, 2016; Papeo et al., 2015; Passeri et al., 2015). TMS studies, utilizing “condition-and-perturb” or “perturb-and-measure” paradigms on semantic processing revealed that TMS effects on the stimulation site might spread to other regions that are structurally or functionally connected to the stimulated area (Hartwigsen et al., 2016; Vitale et al., 2021). This suggests that the broad semantic network might be affected as a whole when targeting a semantic key region, leading to a significant change in semantic task performance. Second, potential compensatory effects within larger networks seem to be strictly constrained by the experimental factors. As pinpointed by Klaus et al. (2020), the compensatory effects of the semantic network might only be observed when TMS intensity and executive control components of the task are relatively low. As a result, semantic task performance can not be maintained to the original level by compensatory effects.

Phonological processing also involves a distributed neural network, but previous work suggests that each region might make relatively independent and unique contributions to phonological processing (Hartwigsen et al., 2010), thus potentially reducing effective compensatory effects among regions and leading to significant effects on task performance. Another finding worth noting is that only ACC but not RT was affected by TMS. This could be due to the fact that phonological processing relies on a larger domain-general verbal working memory system (Deschamps et al., 2014, 2020), and previous studies have shown that TMS effects on working memory tasks tend to be manifested especially on ACC (Mottaghy et al., 2003; Nixon et al., 2004; Romero et al., 2006; Osaka et al., 2007; Acheson et al., 2011). As pointed out by Deschamps et al. (2014), most of the tasks adopted by studies investigating phonological processing actually recruited verbal working memory, such as the same/different judgment task (Deschamps et al., 2014), the delayed auditory discrimination task (Deschamps et al., 2020), and phonological decision tasks (Hartwigsen et al., 2016), all involving sub-vocal rehearsal and the maintenance of phonological information in working memory. Another potential explanation for the observed effects is that compared to the retrieval and decision phase, perturbation of the encoding phase tends to cause changes in ACC as opposed to RT (Karabanov et al., 2015). Most of the included phonological studies in our meta-analysis focused on the posterior inferior frontal gyrus (pIFG), supramarginal gyrus (SMG), and posterior superior temporal gyrus (pSTG), which have been proven to play a role in the rehearsal and encoding phase, but not in the retrieval or decision phase of phonological working memory tasks (Nixon et al., 2004; Kahn et al., 2005; Kirschen et al., 2006).

Compared to semantic and phonological studies, the number of syntactic studies was relatively small, hampering the possibility to obtain a stable significant result and a conclusive interpretation. However, some studies did show a significant TMS effect on syntactic tasks (Uddén et al., 2017; Ishkhanyan et al., 2020; van der Burght et al., 2022). It is also possible that the overall insignificant results are related to the degree to which sub-regions are differentiated. Taking Finocchiaro et al. (2015) as an example, this study probed the function of anterior, middle and posterior parietal sites in thematic role assignment and only found a significant TMS effect for the posterior site. Therefore, for more comprehensive and deeper understanding of TMS neuromodulatory effects on syntactic processing, future studies should adopt different syntactic tasks and focus on specialized sub-regions (such as IFG and pSTG).

### Cortical targets

With regard to stimulation sites, none of the three broad regions (including frontal, temporal, and parietal regions) showed significant TMS effects on RT or ACC. Further analyses at a finer-scale only revealed marginally significant TMS effects on ACC in IFG, but not in STG and MTG.

These results might be interpreted from four directions. First, the basic rationale of TMS studies is to explore, or to be more specific, to verify the potential causal relationship between cognitive functions and certain brain regions based on previous neuroimaging and clinical studies (Flöel, 2012; Papeo et al., 2013; Hartwigsen, 2015). Therefore, the results can be both verification as well as falsification. When analyzing the brain regions alone regardless of the effects of other moderators such as language functions, it seems equally possible to obtain significant as well as non-significant effect. For example, the findings of Bonnì et al. (2015) contradicted the existing clinical studies, showing that TMS on left ATL had no behavioral effects on written word processing. Krieger-Redwood et al. (2013) found that, despite positive evidence from neuroimaging results, TMS over the primary motor cortex (PMC) did not disrupt the mapping of speech sounds onto semantic categories. As a result, future meta-analyses should investigate interactions and try to separate the relationships between different moderators.

Second, it has become increasingly evident that some key language-related brain regions such as IFG and STG can be divided into finer anatomical structures specialized for different functions (Whitney et al., 2012; Klaus and Hartwigsen, 2019; Deschamps et al., 2020; Piai et al., 2020). This brought new challenges for studies to localize the precise stimulation site for the target brain regions underlying certain functions, along with the already existing barriers regarding the limited spatial resolution of TMS (between 0.5 and 1 cm, Sliwinska et al., 2015) and the variance in precision of different methods of localization, leading to the failure to capture significant modulatory effects of TMS. Moreover, it has also been demonstrated that these sub-regions are quite sensitive to task difficulty. For example, in Whitney et al. (2012), stimulation to the anterior inferior frontal gyrus (aIFG) only affected semantic tasks with higher executive control demands, while leaving more automatic tasks unaffected. Future TMS studies are therefore recommended to differentiate not only between specific task types for each language function (i.e., semantic, syntactic and phonological) but also between different degrees of task difficulty within each task.

Third, unlike long-term effects of recovery in patients with brain lesions (Walsh and Cowey, 2000; Krieger-Redwood et al., 2013), the “virtual lesion” caused by TMS may be compensated by rapid functional reorganization within the distributed neural network for language in a rather short time (Hartwigsen et al., 2013, 2016; Klaus and Hartwigsen, 2019), making the transient TMS effects harder to detect. Combined with the finer division of regions and corresponding functions, the implication for future studies is to aim for the network instead of single nodes and target key/hub nodes within the networks for different functions.

Finally, for the marginally significant TMS effects on IFG, we reason that this may reflect the relatively large number of studies investigating this region. Since IFG is the language hub where the classic language region, Broca’s region resides, studies probing semantic, syntactic, or phonological processing could all take IFG into account and have indeed proven its involvement in these three language functions (Krieger-Redwood and Jefferies, 2014; Zhang et al., 2018; Ishkhanyan et al., 2020). On the other hand, the modulatory effects of TMS on STG and MTG are still rather unstable owing to the lack of adequate studies and need to be confirmed by future meta-analysis including more studies.

### Stimulation parameters

The present results support the coordinates of focal stimulation sites (brain regions) extracted from previous studies over the localization determined by the researchers themselves. Taking a closer look at the specific approaches for targeting, we found that studies determining stimulation sites by themselves mostly utilized coarse-grained targeting such as scalp measurement in reference to certain landmarks on the skull (e.g., the inion) (Finocchiaro et al., 2008; Jackson et al., 2015) or standard electrode cap from the EEG 10-20 system (Franzmeier et al., 2012; Jost et al., 2020). These approaches neither account for inter-individual differences in the anatomical structures beneath the scalp nor for the differences in the functional organization of the brain (Sandrini et al., 2011; Beynel et al., 2019). Some studies also combined anatomical magnetic resonance imaging (MRI) scans with the use of frameless stereotaxic neuro-navigation systems to realize more accurate “online” localization of the target site (Zhang et al., 2018; Deschamps et al., 2020), but still, this approach lacks the precision regarding inter-individual differences in structure-to-function relationships. By contrast, relying on anatomical coordinates from previous fMRI studies or meta-analyses with the same task paradigm or tasks probing similar language processing under investigation (Krieger-Redwood and Jefferies, 2014; Passeri et al., 2015) seems to be more promising. This function-guided approach has been proven to be the optimal localization approach with higher experimental power, especially when individual fMRI localizers within the same participants are used (Sack et al., 2009; Sandrini et al., 2011; Beynel et al., 2019).

When considering the stimulation types, we found that rTMS could exert significant neuromodulatory effects on language task performance. This may be because rTMS could prolong the stimulation time, thus accumulating and enhancing the effect sizes (Sandrini et al., 2011). As for TBS, this protocol may be more susceptible to inter-individual differences in focal neuronal states, neural compensation mechanisms, and the specific location within the structurally complex brain regions (Silvanto and Pascual-Leone, 2008; Thut and Pascual-Leone, 2010; Hamada et al., 2013; Vernet et al., 2013; Jost et al., 2020), and thus elicited variable modulatory effects in the present meta-analysis (e.g., Hartwigsen et al., 2013; Bonnì et al., 2015; Jung and Lambon Ralph, 2016).

Our moderator analysis on the timing parameter (i.e., when to apply TMS) demonstrated that online TMS could exert significant neuromodulatory effects especially on RT while offline TMS elicited significant effects on ACC. During online TMS, stimulation is administered immediately before or during the task to transiently disrupt the ongoing neural processing (sometimes referred to as “virtual lesion”). However, it has also been proposed that such online disruption, unlike an actual lesion that would terminate the ongoing process, may rather induce neuronal noise in the targeted area (Devlin and Watkins, 2007; Sandrini et al., 2011). Consequently, online TMS might mainly result in a quantitative change of response efficiency (i.e., as reflected in the response speed), with the quality of response (i.e., the accuracy rates) being spared. Furthermore, according to the “state-dependency” concept (Silvanto and Pascual-Leone, 2008; Miniussi et al., 2013), the induced noise may not be completely random but dependent on the brain state induced by the task, and could turn into part of the signal if it synchronizes with the ongoing neural activity. For example, some studies discovered that TMS given immediately prior to the task could pre-activate related neuron populations and facilitate picture naming speed (Töpper et al., 1998; Mottaghy et al., 1999). Collectively, these findings support the notion that the transient online TMS effect is more likely to affect response efficiency but may not be detrimental enough to disrupt response quality. On the contrary, offline TMS is given before a task, with the aftereffects typically lasting for up to 30 min after the stimulation (Hartwigsen, 2015; Beynel et al., 2019). The accumulated rTMS effects are not restricted to the stimulated sites but may spread to other connected brain regions within a network. Such long-lasting remote effects may modulate the whole network and disrupt or facilitate processing, leading to a perturbation or enhancement in task accuracy.

The current results confirmed that both high and low TMS frequencies could affect healthy participants’ language performance, with high frequencies exerting more stable effects. This accords with a series of studies focusing on the influence of specific stimulation parameters (including frequency) on TMS effects (Sparing et al., 2001; Sollmann et al., 2015, 2018; Nettekoven et al., 2021), which support the idea that higher frequencies may induce more reliable disruption of language functions. There are two possible explanations for this finding. The first explanation is related to potential side effects, especially discomfort or pain during stimulation. The distraction caused by physical discomfort (e.g., twitching and contractions of face muscles) or more severe side effects (e.g., dysarthria resulting from stimulation-induced contraction of cranial muscles, Sollmann et al., 2018) are non-specific TMS effects and are very likely to confound the interpretation of the results. Therefore, it has been proposed that higher frequencies correlated with lower pain levels and were therefore more optimal for obtaining reliable TMS effects (Nettekoven et al., 2021). Secondly, it is likely that using TMS frequencies matching with the natural frequency band of endogenous brain oscillations increases the probability of TMS pulses to interfere with cortical processing at the appropriate timing (Thut and Miniussi, 2009; Miniussi et al., 2013; Nettekoven et al., 2021). Indeed, evidence from MEG studies has associated language-related processing with brain oscillations in higher frequency bands, such as the beta (17–25 Hz) and the low gamma band (26–50 Hz) (Hirata et al., 2010; Hinkley et al., 2020; Youssofzadeh et al., 2020). It is also worth noting that different language brain regions may be sensitive to different stimulation frequencies. However, studies exploring optimal frequencies for distinct regions are still lacking, leaving room for further progress.

Our additional analysis examining the direction of TMS effects revealed no significant results. Yet, we did find that high-frequency rTMS was prone toward inhibition as manifested by the ACC decrease. This supports the notion that a simple transfer of the relationship between frequency type and effect direction (i.e., high frequency for facilitation, and low frequency for inhibition, see Bailey et al., 2001; Fitzgerald et al., 2006; Murdoch and Barwood, 2013) from the motor to the language system does not hold. Rather, this relationship may be influenced by multiple factors such as task types, stimulation intensities, and target brain regions (Vallar and Bolognini, 2011; Hartwigsen, 2015; Beynel et al., 2019). Nevertheless, no strong conclusion could be made given the non-significant results.

The moderator analysis also showed that compared to the intensities calibrated according to the AMT, RMT could exert more significant TMS effects on language performance. This result is not surprising and directly relates to higher stimulation intensities. RMT is typically defined as the lowest amount of stimulator output (intensity) necessary to produce a motor evoked potential (MEP) in the resting muscle exceeding 50 μV in at least 50% of the total trials. In contrast, AMT is assessed under voluntary pre-contraction of the target muscle, requiring MEP sizes of at least 150 μV (Sandrini et al., 2011). A a result, individual RMTs are usually considerably higher (approximately 15%) than AMTs and consequently leading to a higher TMS intensity, which was proposed to introduce more severe perturbations, rendering functional compensation more difficult (Klaus et al., 2020), and exert stronger remote, long-distance effects spreading across specialized networks (Hartwigsen, 2015). Also, researches (although rare) distinguishing between RMT and AMT (e.g., Wassermann, 2002) argued that experimental error and other unstable determinants of threshold may account for about 36% of the cross-subject variability at rest and about 50% during voluntary contraction, which suggests that compared to RMT, AMT is more vulnerable to factors such as coil placement, stimulation frequency and other unknown physiological sources of individual variability and is therefore a less stable reference to determine the stimulation intensity.

### Experimental designs

Regarding control conditions, sham TMS outperformed other solutions. It is noteworthy that TMS on presumably unrelated control sites might elicit unwanted effects due to their connections with target sites, thus potentially confounding experimental and control conditions (Franzmeier et al., 2012; Papeo et al., 2015; Klaus and Hartwigsen, 2019; Vitale et al., 2021). Therefore, researchers should be very cautious when selecting control sites. It should also be emphasized that the inclusion of sham TMS alone does not control for potential side effects of the stimulation such as muscle twitches and pain. Consequently, such conditions are not sufficient and studies without active control sites are more prone to false positives (Jost et al., 2020; Vitale et al., 2022). The optimal TMS study on language should include both an active control site and sham stimulation.

As for the group design types, within-subject designs were less affected by the individual variance which might submerge the TMS effects in between-subject designs (see also Passeri et al., 2015; Zhang et al., 2018). For example, Zhang et al. (2018) adopted a between-subject design and considered this a major limitation due to the large individual variance. Passeri et al. (2015) further emphasized that the neuromodulatory TMS effects are largely affected by the different degrees of language-related brain region lateralization of individuals. Therefore, the individual brain’s structural and functional variance might be critical for identifying TMS effects.

## Limitations and outlook

The present systematic meta-analysis provides first insights into TMS neuromodulatory effects on language performance in healthy adults, elucidating both overall as well as specific effects regarding the moderators of language tasks, cortical targets, stimulation parameters, and experimental designs, and therefore identifies conditions more prone to elicit robust TMS effects. However, it is premature to draw strong conclusions about a “perfect TMS study design or protocol” in neurolinguistics, as TMS effects are moderated by the various factors stated above.

Besides, due to the limitation of the sample sizes (i.e., the number of studies and participants per study), the classification of these factors at a finer-grained level seems to remain challenging. For instance, sample sizes of the specific task types for each language function (such as different tasks for semantic processing), and some specific brain regions within each lobe (e.g., the PC within the frontal lobe, the ATL within the temporal lobe and the AG within the parietal lobe) were comparatively small, making the TMS effects at a more specific level challenging to evaluate. Moreover, regarding the meta-analysis approach, the readers should be cautious that we mainly focused on the absolute effect sizes in the current meta-analyses, and that the findings concerning the effect directions were relatively limited, thus awaiting to be explored in future studies. We did not perform a multiple-factor analysis either, that is, analyzing the TMS effects when considering the influences from several factors simultaneously owing to the more demanding analysis technique. Future studies may address these issues in a more profound fashion, and complement our current results and assumptions with more evidence and specific designs.

## Data availability statement

The original contributions presented in this study are included in the article/Supplementary material, further inquiries can be directed to the corresponding author.

## Author contributions

XQ, ZW, and LC came up with the idea of this study and conducted the meta-analysis. XQ, ZW, YC, QX, ZL, and LL conducted the searching, screening, and coding of the included studies. XQ, ZW, LC, and GH completed the first draft of this manuscript, which was further revised by YC, QX, ZL, LL, and LF. All authors participated in the discussion of the results and prepared the final version of the manuscript for submission.
