# Supplementary material for: Neuromodulatory effects of transcranial magnetic stimulation on language performance in healthy participants: Systematic review and meta-analysis
Source: Front Hum Neurosci. 2022 Dec 5;16:1027446. doi: 10.3389/fnhum.2022.1027446 (PMC9760723; doi:10.3389/fnhum.2022.1027446)
Supplement: Supplementary file 1 [file Data_Sheet_1.pdf]

Supplementary Material

1 Methods of Dealing with Correlated Effect Size

In previous studies, there were five commonly used methods to deal with correlated effect size, as shown below:

(a) Ignore dependencies. Correlations are ignored and each data point is considered as independent when conducting data analysis. Due to the inadequate estimation of uncertainty, the associated standard errors (SEs) of parameter estimates may be underestimated. Therefore, the relevant significance tests might be exaggerated (Cheung , 2014).

(b) Average dependent effect sizes across studies. This method averages multiple correlated effect sizes into a single effect size, which is a method of aggregation. However, differences between effect sizes are artificially reduced and other informative differences will also disappear (Hedges and Pigott, 2004).

(c) Extract only one effect size for each study. Also known as elimination, this method selects one of several correlated effect sizes in each study, and the selected effect size can be regarded as independent. The statistical power of meta-analyses and the precision of parameter estimation would be affected owing to the exclusion of the non-selected effect sizes (Van den Noortgate et al., 2013).

(d) Introduce the correlation coefficient *r*. Researchers often use a fixed value, such as r = 0.75, r = 0.7 or r = 0.5 to correct the correlation of a within-subject design. Since most experiments do not report the correlation between conditions, the estimation of *r*-value tends to be conservative and somewhat arbitrary, especially when the correlation is high (Hedges and Pigott, 2001; Cheung, 2014).

(e) Adopt modeling, such as the frequently used multilevel random effects model. Using multilevel random effects model to estimate the effect sizes in meta-analyses is more accurate, effective and flexible. The model can incorporate multiple predictors to account for heterogeneity between studies or add additional random effects to address the various dependencies of effect sizes within and between studies (Fernández-Castilla et al., 2020).

Therefore, to solve the problem of effect size dependency especially for within-subject designs, a three-level random effects model was used for the meta-analysis in this study.

Raudenbush and Bryk (1985) proposed that a meta-analysis is a special case of multilevel analysis using aggregated data instead of raw data and differentiating between the intra- and inter-study models. We started with the traditional two-level random effects model and proceeded with a step-by-step derivation to the three-level random effects model adopted in this study. In multilevel notation, the intra-study model is as follows:

$$d_k = \gamma_k + r_k \qquad (1)$$

In this equation, d_k represents the reported effect size in study k, γ_k represents the overall effect size in study k, and r_k is the sampling error, which is estimated prior to the meta-analysis. The inter-study model simulates the variation in effect sizes among studies and is shown as follows:

$$\gamma_k = \delta_0 + u_k \qquad (2)$$

In this equation, δ_0 is the pooled effect size and u_k is the random residual error with zero expected value. It is usually assumed in the model that the study residuals follow a normal distribution of variance. Thus, the combined equation of this model is:

$$d_k = \delta_0 + u_k + r_k \qquad (3)$$

This is the traditional two-level random effects model commonly used in meta-analyses, which again corroborates the notion that random effects meta-analyses are actually multilevel models (Pastor and Lazowski, 2018).

In this study, a three-level random effects model was adopted with the following equation:

$$d_{jk} = \delta_{00} + V_k + U_{jk} + W_{ik} \qquad (4)$$

In this equation, k stands for individual study, j for experimental condition, and the condition j in study k is represented as j_k. djk represents the observed effect size of condition j in study k, and δ_{00} refers to the overall mean effect size across all conditions and studies. V_k is the random deviation between the effect size of study k and the overall effect size. U_{jk} is the deviation between the effect size of condition j in study k and the average effect size in study k; i refers to the category to which study k belongs (in this study, there are two categories, namely the within-subject and between-subject design. Note that the mixed design was collapsed into within-subject or between-subject design according to the type of data presented), W_{ik} refers to the deviation of study k of category i from the overall average effect size. Each error item is assumed to have a mean of zero, which satisfies an independent normal distribution.

References

Cheung M. W. (2014). Modeling dependent effect sizes with three-level meta-analyses: a structural equation modeling approach. *Psychol Methods*. 19(2), 211–229. doi: 10.1037/a0032968

Fernández-Castilla, B., Jamshidi, L., Declercq, L., Beretvas, S. N., Onghena, P., and Van den Noortgate, W. (2020). The application of meta-analytic (multi-level) models with multiple random effects: A systematic review. *Behav Res Methods.* 52(5), 2031–2052. doi: 10.3758/s13428-020-01373-9

Hedges, L. V., and Pigott, T. D. (2001). The power of statistical tests in meta-analysis. *Psychol Methods.* 6(3), 203–217. doi: 10.1037/1082-989X.6.3.203

Hedges, L. V., and Pigott, T. D. (2004). The power of statistical tests for moderators in meta-analysis. *Psychol Methods.* 9(4), 426–445. doi: 10.1037/1082-989X.9.4.426

Pastor, D. A., and Lazowski, R. A. (2018). On the Multilevel Nature of Meta-Analysis: A Tutorial, Comparison of Software Programs, and Discussion of Analytic Choices. *Multivariate Behav Res.* 53(1), 74–89. doi: 10.1080/00273171.2017.1365684

Raudenbush, S. W., and Bryk, A. S. (1985). Empirical Bayes meta-analysis. *J Educ Stat*, 10, 75–98.

Van den Noortgate, W., López-López, J. A., Marín-Martínez, F., and Sánchez-Meca, J. (2013). Three-level meta-analysis of dependent effect sizes. *Behav Res Methods.* 45(2), 576–594. doi: 10.3758/s13428-012-0261-6

2 Reaction Time (RT) Moderator-analyses

The Reaction Time (RT) results of all moderator-analyses were shown in Supplementary Figure S1-S30. See catalogue for page numbers. Note that additional analyses investigating effect directions were marked with "*".

Catalogue

1 Tasks.......... 4
2 Cortex.......... 7
3 Stimulation parameters.......... 13
4 Experimental design.......... 27

3 Accuracy (ACC) Moderator-analyses

The Accuracy (ACC) results of all moderator-analyses were shown in Supplementary Figure S31-S60. See catalogue for page numbers.

Catalogue

1 Tasks.......... 34
2 Cortex.......... 37
3 Stimulation parameters.......... 43
4 Experimental design.......... 57

Author(s) and Year	Experimental Total	Experimental Mean	Experimental SD	Control Total	Control Mean	Control SD	Hedges'g [95% CI]
Sakai et al. (2002)	6	−651.4	214.01	6	−657.1	192.35	0.02 [−1.11, 1.16]
Schuhmann et al. (2009)	10	475	53.82	8	454	38.05	0.40 [−0.54, 1.34]
Hartwigsen et al. (2010), Exp1	28	1070.67	1371.93	7	755.83	328.64	0.24 [−0.59, 1.07]
Hartwigsen et al. (2010), Exp2	28	1022.5	1024.46	7	686	113.5	0.35 [−0.49, 1.18]
Willems et al. (2011)	18	721	184.42	8	662.77	187.73	0.29 [−0.54, 1.13]
Andoh et al. (2011)	10	962.5	462.63	10	962	433.3	0.00 [−0.88, 0.88]
Schuhmann et al. (2012), Exp1	8	471	51.89	8	455	44	0.30 [−0.69, 1.28]
Schuhmann et al. (2012), Exp2	8	488.2	40.01	8	474	39	0.32 [−0.67, 1.31]
Schuhmann et al. (2012), Exp3	8	477	39.61	8	470	29	0.18 [−0.80, 1.16]
Hartwigsena et al. (2013), Exp1	17	916.5	46.61	17	899.25	46.38	0.35 [−0.32, 1.03]
Hartwigsena et al. (2013), Exp2	17	916.5	46.61	17	912.25	44.85	0.09 [−0.58, 0.76]
Jackson et al. (2015)	15	1189.43	282.59	15	1134.04	302.41	0.18 [−0.54, 0.90]
Papeo et al. (2015), Exp1	14	−1033.7	261.45	14	−1045.67	163.54	0.05 [−0.69, 0.79]
Papeo et al. (2015), Exp2	14	1033.7	261.45	14	1008.9	293.48	0.08 [−0.66, 0.83]
Passeri et al. (2015), Exp1	18	578	23	18	567	22.02	0.47 [−0.20, 1.13]
Passeri et al. (2015), Exp2	18	574	26.62	18	567	22.02	0.27 [−0.38, 0.93]
Davey et al. (2015), Exp1	15	1293.67	451.24	15	1291.3	439.47	0.01 [−0.71, 0.72]
Davey et al. (2015), Exp2	15	−1259.2	439.12	15	−1291.3	439.47	0.07 [−0.65, 0.79]
Hartwigsen et al. (2016), Exp1	17	−973.16	700.55	17	−1021.29	727.83	0.06 [−0.61, 0.74]
Hartwigsen et al. (2016), Exp2	17	−1002.23	498.42	17	−1025.43	625.5	0.04 [−0.63, 0.71]
Woollamsa et al. (2017)	18	−548	72.28	18	−550.38	66.93	0.03 [−0.62, 0.69]
Klaus et al. (2018), Exp1	24	1789.5	432	24	1773	432	0.04 [−0.53, 0.60]
Klaus et al. (2018), Exp2	24	−1769.5	492.15	24	−1807	528	0.07 [−0.49, 0.64]
Zhang et al. (2018)	21	584.69	183.18	21	557.45	220.53	0.13 [−0.48, 0.73]
Klaus et al. (2019)	16	−608	191.44	16	−614.5	198.27	0.03 [−0.66, 0.72]
Jost et al. (2020)	22	1219	241	22	1178	239	0.16 [−0.43, 0.76]
Piai et al. (2020)	24	−834.67	113.44	24	−840.17	112.87	0.05 [−0.52, 0.61]
Sliwinska et al. (2021)	20	1286.5	188.3	20	1279	176.16	0.04 [−0.58, 0.66]
Finocchiaro et al. (2021)	16	−1742.12	1192.34	16	−1748	1142.47	0.00 [−0.69, 0.70]
Ward et al. (2022)	20	651.67	119.58	20	642	109.74	0.08 [−0.54, 0.70]
van der Burght et al. (2022), Exp2	30	894.78	327.97	30	878.17	291.38	0.05 [−0.45, 0.56]
Random Effect Model Total (Z = 2.002, P = 0.045)							0.13 [0.00, 0.25]

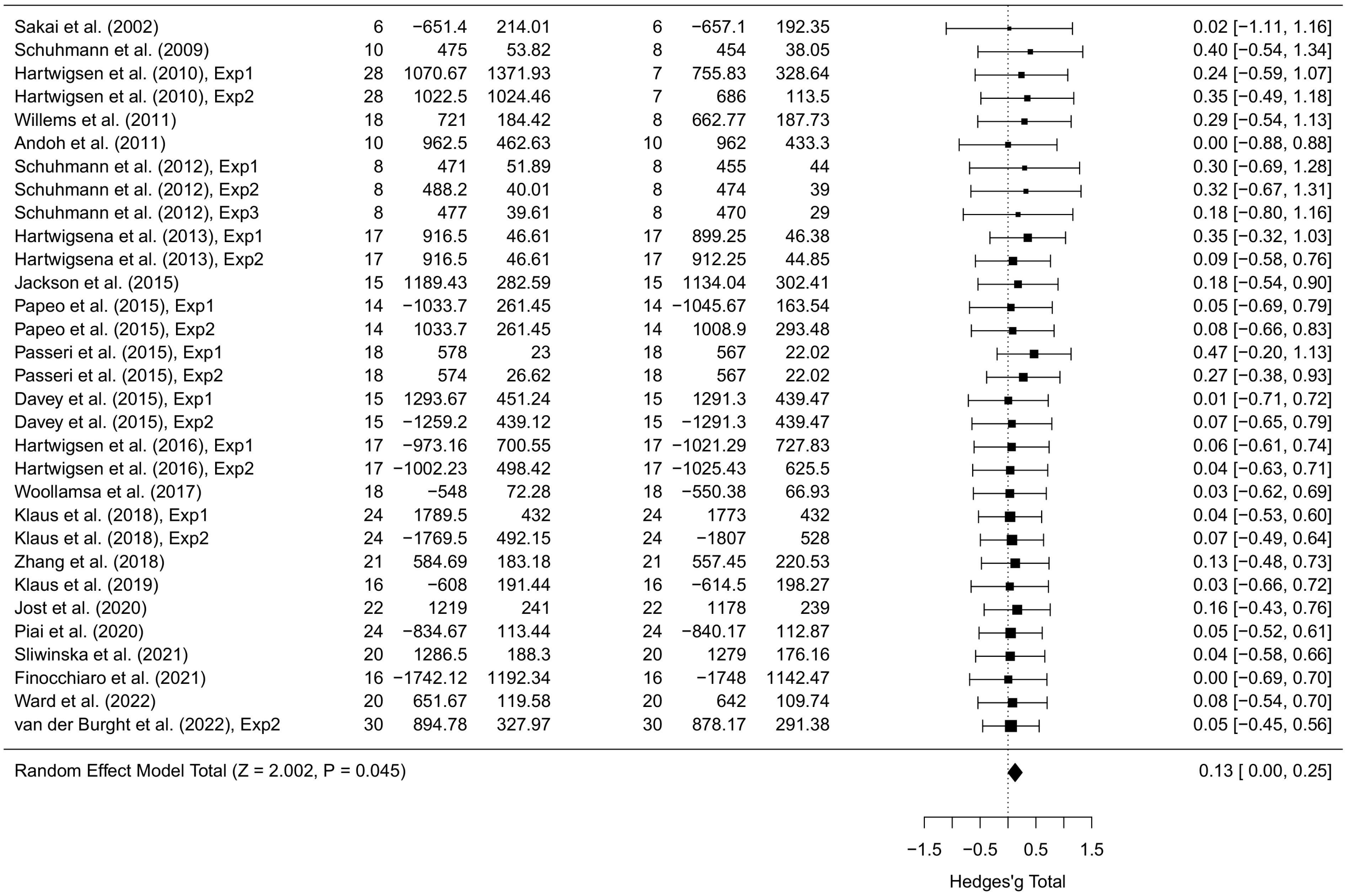

Supplementary Figure S1. Forest plot of RT effect moderator-analysis of semantic tasks

Author(s) and Year	Experimental Total	Experimental Mean	Experimental SD	Control Total	Control Mean	Control SD	Hedges'g [95% CI]
Sakai et al. (2002)	6	−634.62	200.01	6	−641.5	177.05	0.03 [−1.10, 1.16]
Finocchiaro et al. (2008)	6	−51	55.15	5	−87.5	132.38	0.31 [−0.88, 1.51]
Finocchiaro et al. (2015), Exp1	12	−1120.33	375.36	18	−1381.27	274.8	0.78 [0.02, 1.53]
Finocchiaro et al. (2015), Exp2	12	−1151.13	305.64	18	−1381.27	274.8	0.76 [0.00, 1.51]
Finocchiaro et al. (2015), Exp3	12	−1133.38	242.97	18	−1381.27	274.8	0.89 [0.13, 1.66]
Ishkhanyan et al. (2020), Exp1	19	−558	115.05	19	−562	108.59	0.03 [−0.60, 0.67]
Ishkhanyan et al. (2020), Exp2	19	585	115.05	19	559	123.13	0.21 [−0.43, 0.85]
Finocchiaro et al. (2021)	16	−1742.12	1192.34	16	−1748	1142.47	0.00 [−0.69, 0.70]
van der Burght et al. (2022), Exp1	30	−698.92	265.17	30	−715.04	242.57	0.06 [−0.44, 0.57]
Random Effect Model Total (Z = 1.268, P = 0.205)							0.31 [−0.17, 0.78]

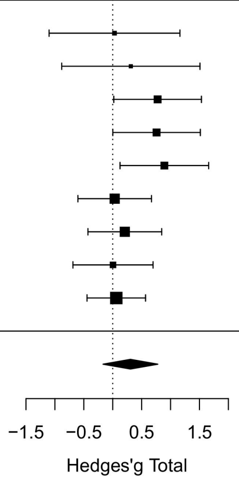

Supplementary Figure S2. Forest plot of RT effect moderator-analysis of syntactic tasks

Author(s) and Year	Experimental Total	Experimental Mean	Experimental SD	Control Total	Control Mean	Control SD	Hedges'g [95% CI]
Schuhmann et al. (2009)	10	475	53.82	8	454	38.05	0.40 [−0.54, 1.34]
Hartwigsen et al. (2010), Exp1	28	1070.67	1371.93	7	755.83	328.64	0.24 [−0.59, 1.07]
Hartwigsen et al. (2010), Exp2	28	1022.5	1024.46	7	686	113.5	0.35 [−0.49, 1.18]
Andoh et al. (2011)	10	962.5	462.63	10	962	433.3	0.00 [−0.88, 0.88]
Schuhmann et al. (2012), Exp1	8	471	51.89	8	455	44	0.30 [−0.69, 1.28]
Schuhmann et al. (2012), Exp2	8	488.2	40.01	8	474	39	0.32 [−0.67, 1.31]
Schuhmann et al. (2012), Exp3	8	477	39.61	8	470	29	0.18 [−0.80, 1.16]
Hartwigsena et al. (2013), Exp1	17	916.5	46.61	17	899.25	46.38	0.35 [−0.32, 1.03]
Hartwigsena et al. (2013), Exp2	17	916.5	46.61	17	912.25	44.85	0.09 [−0.58, 0.76]
Karabanov et al. (2015), Exp1	26	949	8060	26	861	9048	0.01 [−0.53, 0.55]
Karabanov et al. (2015), Exp2	26	−781	8970	26	−829	7384	0.01 [−0.54, 0.55]
Hartwigsen et al. (2016), Exp1	17	−973.16	700.55	17	−1021.29	727.83	0.06 [−0.61, 0.74]
Hartwigsen et al. (2016), Exp2	17	−1002.23	498.42	17	−1025.43	625.5	0.04 [−0.63, 0.71]
Klaus et al. (2018), Exp1	24	1789.5	432	24	1773	432	0.04 [−0.53, 0.60]
Klaus et al. (2018), Exp2	24	−1769.5	492.15	24	−1807	528	0.07 [−0.49, 0.64]
Zhang et al. (2018)	21	584.69	183.18	21	557.45	220.53	0.13 [−0.48, 0.73]
Klaus et al. (2019)	16	−608	191.44	16	−614.5	198.27	0.03 [−0.66, 0.72]
Jost et al. (2020)	22	1219	241	22	1178	239	0.16 [−0.43, 0.76]
Ward et al. (2022)	20	651.67	119.58	20	642	109.74	0.08 [−0.54, 0.70]
Random Effect Model Total (Z = 1.481, P = 0.139)							0.12 [−0.04, 0.28]

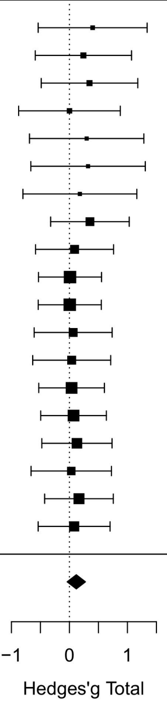

Supplementary Figure S3. Forest plot of RT effect moderator-analysis of phonological tasks

Author(s) and Year	Experimental Total	Experimental Mean	Experimental SD	Control Total	Control Mean	Control SD	Hedges'g [95% CI]
Sakai et al. (2002)	6	−646.88	210.81	6	−651.44	186.39	0.02 [−1.11, 1.15]
Finocchiaro et al. (2008)	6	−51	55.15	5	−87.5	132.38	0.31 [−0.88, 1.51]
Schuhmann et al. (2009)	10	475	53.82	8	454	38.05	0.40 [−0.54, 1.34]
Hartwigsen et al. (2010), Exp1	28	1070.67	1371.93	7	755.83	328.64	0.24 [−0.59, 1.07]
Hartwigsen et al. (2010), Exp2	28	1022.5	1024.46	7	686	113.5	0.35 [−0.49, 1.18]
Schuhmann et al. (2012), Exp1	8	471	51.89	8	455	44	0.30 [−0.69, 1.28]
Hartwigsena et al. (2013), Exp1	17	916.5	46.61	17	899.25	46.38	0.35 [−0.32, 1.03]
Hartwigsena et al. (2013), Exp2	17	916.5	46.61	17	912.25	44.85	0.09 [−0.58, 0.76]
Karabanov et al. (2015), Exp2	26	−781	8970	26	−829	7384	0.01 [−0.54, 0.55]
Hartwigsen et al. (2016), Exp1	17	−983.34	683.58	17	−1021.38	706.44	0.05 [−0.62, 0.72]
Klaus et al. (2018), Exp1	24	1793.5	469.38	24	1790	482.39	0.01 [−0.56, 0.57]
Klaus et al. (2018), Exp2	24	−1765.5	456.63	24	−1790	482.39	0.05 [−0.52, 0.62]
Zhang et al. (2018)	21	584.69	183.18	21	557.45	220.53	0.13 [−0.48, 0.73]
Ishkhanyan et al. (2020), Exp1	19	−559.33	116.35	19	−560.17	116.87	0.01 [−0.63, 0.64]
Ishkhanyan et al. (2020), Exp2	19	559	118.65	19	530.4	128.86	0.22 [−0.42, 0.86]
Jost et al. (2020)	22	1219	241	22	1178	239	0.16 [−0.43, 0.76]
van der Burght et al. (2022), Exp1	30	−698.92	265.17	30	−715.04	242.57	0.06 [−0.44, 0.57]
van der Burght et al. (2022), Exp2	30	894.78	327.97	30	878.17	291.38	0.05 [−0.45, 0.56]
Random Effect Model Total (Z = 1.514, P = 0.13)							0.12 [−0.04, 0.28]

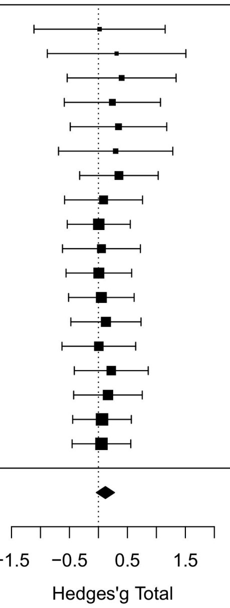

Supplementary Figure S4. Forest plot of RT effect moderator-analysis of frontal

Author(s) and Year	Experimental Total	Experimental Mean	Experimental SD	Control Total	Control Mean	Control SD	Hedges'g [95% CI]
Andoh et al. (2011)	10	−934.5	520.38	10	−957	525.59	0.04 [−0.84, 0.92]
Schuhmann et al. (2012), Exp2	8	488.2	40.01	8	474	39	0.32 [−0.67, 1.31]
Schuhmann et al. (2012), Exp3	8	477	39.61	8	470	29	0.18 [−0.80, 1.16]
Passeri et al. (2015), Exp1	18	578	23	18	567	22.02	0.47 [−0.20, 1.13]
Karabanov et al. (2015), Exp1	26	949	8060	26	861	9048	0.01 [−0.53, 0.55]
Davey et al. (2015), Exp1	15	1293.67	451.24	15	1291.3	439.47	0.01 [−0.71, 0.72]
Woollamsa et al. (2017)	18	−548	72.28	18	−550.38	66.93	0.03 [−0.62, 0.69]
Deschamps et al. (2020)	18	11.36	491.91	18	9.05	412.25	0.00 [−0.65, 0.66]
Piai et al. (2020)	24	−834.67	113.44	24	−840.17	112.87	0.05 [−0.52, 0.61]
Ward et al. (2022)	20	667	119.1	20	641.5	109.54	0.21 [−0.41, 0.84]
Random Effect Model Total (Z = 1.045, P = 0.296)							0.12 [−0.10, 0.33]

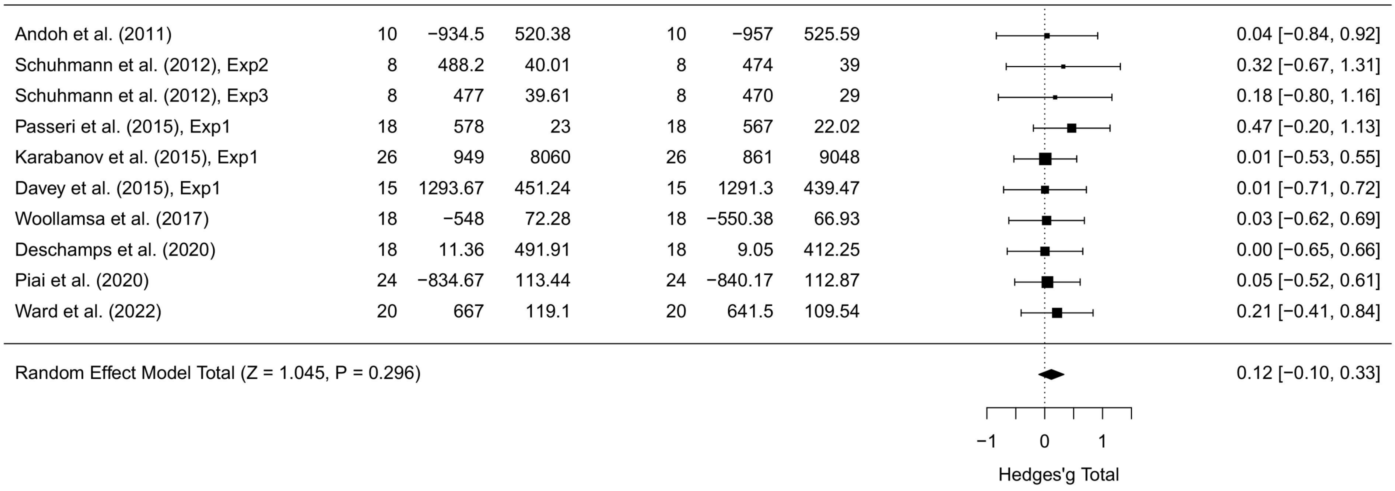

Supplementary Figure S5. Forest plot of RT effect moderator-analysis of methods of temporal

Author(s) and Year	Experimental Total	Experimental Mean	Experimental SD	Control Total	Control Mean	Control SD	Hedges'g [95% CI]
Finocchiaro et al. (2015), Exp1	12	−1120.33	375.36	18	−1381.27	274.8	0.78 [0.02, 1.53]
Finocchiaro et al. (2015), Exp2	12	−1151.13	305.64	18	−1381.27	274.8	0.76 [0.00, 1.51]
Finocchiaro et al. (2015), Exp3	12	−1133.38	242.97	18	−1381.27	274.8	0.89 [0.13, 1.66]
Hartwigsen et al. (2016), Exp1	17	−962.97	717.11	17	−1021.2	748.62	0.08 [−0.60, 0.75]
Sliwinska et al. (2021)	20	1286.5	188.3	20	1279	176.16	0.04 [−0.58, 0.66]
Finocchiaro et al. (2021)	16	−1742.12	1192.34	16	−1748	1142.47	0.00 [−0.69, 0.70]
Ward et al. (2022)	20	−642.5	119.78	20	−647.5	110.94	0.04 [−0.58, 0.66]
Random Effect Model Total (Z = 1.146, P = 0.252)							0.31 [−0.22, 0.84]

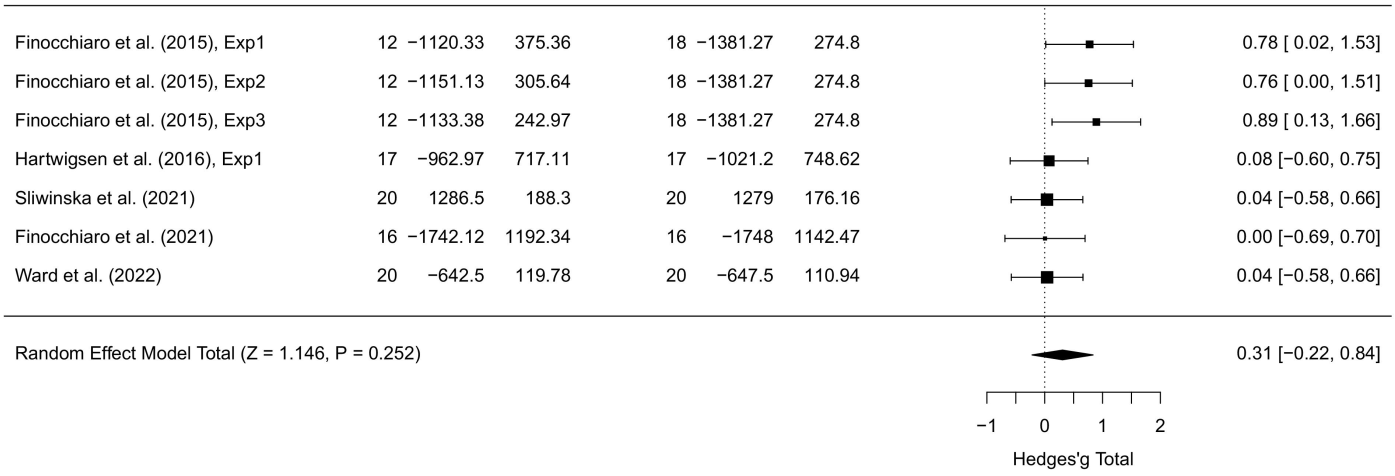

Supplementary Figure S6. Forest plot of RT effect moderator-analysis of parietal

Author(s) and Year	Experimental Total	Experimental Mean	Experimental SD	Control Total	Control Mean	Control SD	Hedges'g [95% CI]
Sakai et al. (2002)	6	−646.88	210.81	6	−651.44	186.39	0.02 [−1.11, 1.15]
Finocchiaro et al. (2008)	6	−51	55.15	5	−87.5	132.38	0.31 [−0.88, 1.51]
Schuhmann et al. (2009)	10	475	53.82	8	454	38.05	0.40 [−0.54, 1.34]
Hartwigsen et al. (2010), Exp1	28	1070.67	1371.93	7	755.83	328.64	0.24 [−0.59, 1.07]
Hartwigsen et al. (2010), Exp2	28	1022.5	1024.46	7	686	113.5	0.35 [−0.49, 1.18]
Schuhmann et al. (2012), Exp1	8	471	51.89	8	455	44	0.30 [−0.69, 1.28]
Hartwigsena et al. (2013), Exp1	17	916.5	46.61	17	899.25	46.38	0.35 [−0.32, 1.03]
Hartwigsena et al. (2013), Exp2	17	916.5	46.61	17	912.25	44.85	0.09 [−0.58, 0.76]
Karabanov et al. (2015), Exp2	26	−781	8970	26	−829	7384	0.01 [−0.54, 0.55]
Hartwigsen et al. (2016), Exp1	17	−983.34	683.58	17	−1021.38	706.44	0.05 [−0.62, 0.72]
Klaus et al. (2018), Exp1	24	1789.5	432	24	1773	432	0.04 [−0.53, 0.60]
Klaus et al. (2018), Exp2	24	−1769.5	492.15	24	−1807	528	0.07 [−0.49, 0.64]
Zhang et al. (2018)	21	584.69	183.18	21	557.45	220.53	0.13 [−0.48, 0.73]
Ishkhanyan et al. (2020), Exp1	19	−559.33	116.35	19	−560.17	116.87	0.01 [−0.63, 0.64]
Ishkhanyan et al. (2020), Exp2	19	559	118.65	19	530.4	128.86	0.22 [−0.42, 0.86]
van der Burght et al. (2022), Exp1	30	−698.92	265.17	30	−715.04	242.57	0.06 [−0.44, 0.57]
van der Burght et al. (2022), Exp2	30	894.78	327.97	30	878.17	291.38	0.05 [−0.45, 0.56]
Random Effect Model Total (Z = 1.47, P = 0.141)							0.12 [−0.04, 0.28]

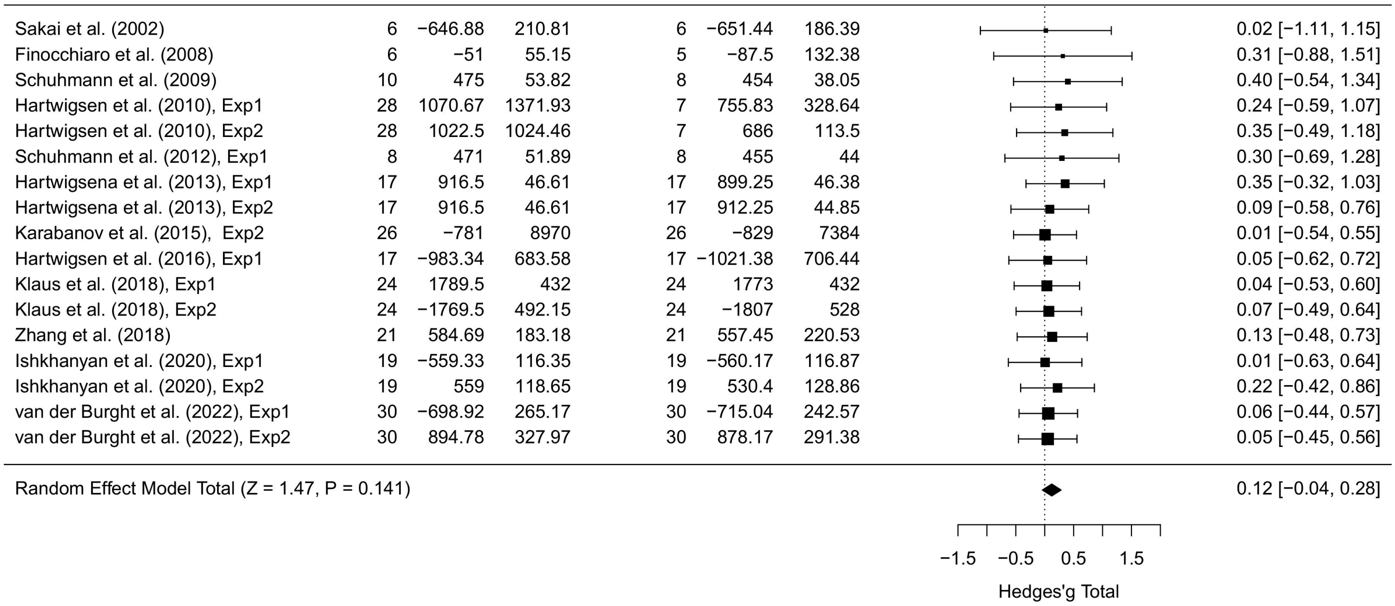

Supplementary Figure S7. Forest plot of RT effect moderator-analysis of IFG

Author(s) and Year	Experimental Total	Experimental Mean	Experimental SD	Control Total	Control Mean	Control SD	Hedges'g [95% CI]
Schuhmann et al. (2012), Exp3	8	477	39.61	8	470	29	0.18 [−0.80, 1.16]
Karabanov et al. (2015), Exp1	26	949	8060	26	861	9048	0.01 [−0.53, 0.55]
Piai et al. (2020)	24	−834.67	113.44	24	−840.17	112.87	0.05 [−0.52, 0.61]
Random Effect Model Total (Z = 0.262, P = 0.793)							0.05 [−0.32, 0.41]

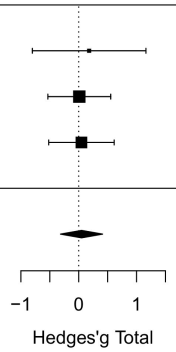

Supplementary Figure S8. Forest plot of RT effect moderator-analysis of STG

Author(s) and Year	Experimental Total	Experimental Mean	Experimental SD	Control Total	Control Mean	Control SD	Hedges'g [95% CI]
Schuhmann et al. (2012), Exp2	8	488.2	40.01	8	474	39	0.32 [−0.67, 1.31]
Papeo et al. (2015), Exp1	14	−1033.7	261.45	14	−1045.67	163.54	0.05 [−0.69, 0.79]
Papeo et al. (2015), Exp2	14	1033.7	261.45	14	1008.9	293.48	0.08 [−0.66, 0.83]
Klaus et al. (2019)	16	−608	191.44	16	−614.5	198.27	0.03 [−0.66, 0.72]
Random Effect Model Total (Z = 0.485, P = 0.628)							0.10 [−0.29, 0.48]

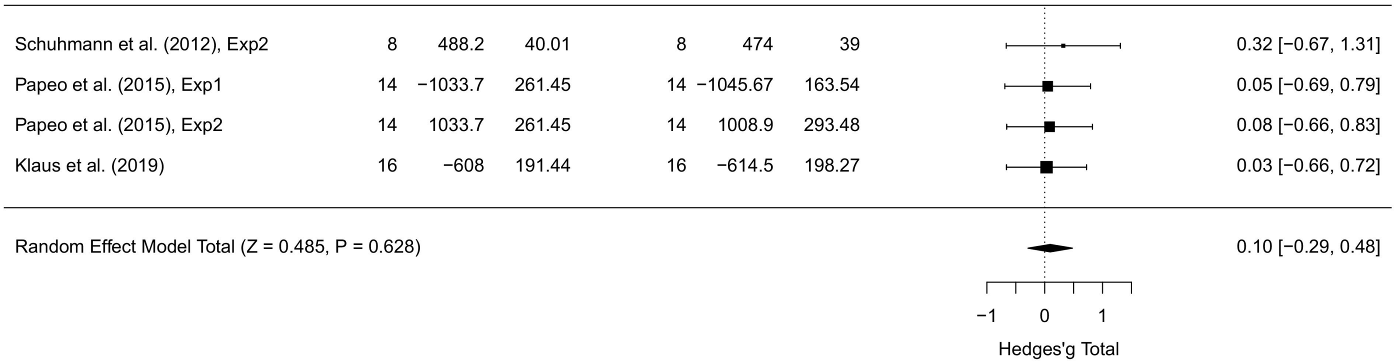

Supplementary Figure S9. Forest plot of RT effect moderator-analysis of MTG

Author(s) and Year	Experimental Total	Experimental Mean	Experimental SD	Control Total	Control Mean	Control SD	Hedges'g [95% CI]
Finocchiaro et al. (2008)	6	−51	55.15	5	−87.5	132.38	0.31 [−0.88, 1.51]
Schuhmann et al. (2009)	10	475	53.82	8	454	38.05	0.40 [−0.54, 1.34]
Hartwigsen et al. (2010), Exp1	28	1070.67	1371.93	7	755.83	328.64	0.24 [−0.59, 1.07]
Hartwigsen et al. (2010), Exp2	28	1022.5	1024.46	7	686	113.5	0.35 [−0.49, 1.18]
Willems et al. (2011)	18	721	184.42	8	662.77	187.73	0.29 [−0.54, 1.13]
Andoh et al. (2011)	10	962.5	462.63	10	962	433.3	0.00 [−0.88, 0.88]
Schuhmann et al. (2012), Exp1	8	471	51.89	8	455	44	0.30 [−0.69, 1.28]
Schuhmann et al. (2012), Exp2	8	488.2	40.01	8	474	39	0.32 [−0.67, 1.31]
Schuhmann et al. (2012), Exp3	8	477	39.61	8	470	29	0.18 [−0.80, 1.16]
Hartwigsena et al. (2013), Exp1	17	916.5	46.61	17	899.25	46.38	0.35 [−0.32, 1.03]
Hartwigsena et al. (2013), Exp2	17	916.5	46.61	17	912.25	44.85	0.09 [−0.58, 0.76]
Papeo et al. (2015), Exp1	14	−1033.7	261.45	14	−1045.67	163.54	0.05 [−0.69, 0.79]
Papeo et al. (2015), Exp2	14	1033.7	261.45	14	1008.9	293.48	0.08 [−0.66, 0.83]
Passeri et al. (2015), Exp1	18	578	23	18	567	22.02	0.47 [−0.20, 1.13]
Passeri et al. (2015), Exp2	18	574	26.62	18	567	22.02	0.27 [−0.38, 0.93]
Davey et al. (2015), Exp1	15	1293.67	451.24	15	1291.3	439.47	0.01 [−0.71, 0.72]
Davey et al. (2015), Exp2	15	−1259.2	439.12	15	−1291.3	439.47	0.07 [−0.65, 0.79]
Hartwigsen et al. (2016), Exp1	17	−973.16	700.55	17	−1021.29	727.83	0.06 [−0.61, 0.74]
Hartwigsen et al. (2016), Exp2	17	−1002.23	498.42	17	−1025.43	625.5	0.04 [−0.63, 0.71]
Woollamsa et al. (2017)	18	−548	72.28	18	−550.38	66.93	0.03 [−0.62, 0.69]
Klaus et al. (2018), Exp1	24	1793.5	469.38	24	1790	482.39	0.01 [−0.56, 0.57]
Klaus et al. (2018), Exp2	24	−1765.5	456.63	24	−1790	482.39	0.05 [−0.52, 0.62]
Zhang et al. (2018)	21	584.69	183.18	21	557.45	220.53	0.13 [−0.48, 0.73]
Klaus et al. (2019)	16	−608	191.44	16	−614.5	198.27	0.03 [−0.66, 0.72]
Ishkhanyan et al. (2020), Exp1	19	−559.33	116.35	19	−560.17	116.87	0.01 [−0.63, 0.64]
Ishkhanyan et al. (2020), Exp2	19	559	118.65	19	530.4	128.86	0.22 [−0.42, 0.86]
Piai et al. (2020)	24	−834.67	113.44	24	−840.17	112.87	0.05 [−0.52, 0.61]
Sliwinska et al. (2021)	20	1286.5	188.3	20	1279	176.16	0.04 [−0.58, 0.66]
Finocchiaro et al. (2021)	16	−1742.12	1192.34	16	−1748	1142.47	0.00 [−0.69, 0.70]
Ward et al. (2022)	20	651.67	119.58	20	642	109.74	0.08 [−0.54, 0.70]
van der Burght et al. (2022), Exp1	30	−703.43	259.2	30	−704.88	258.58	0.01 [−0.50, 0.51]
van der Burght et al. (2022), Exp2	30	−884.81	314.52	30	−889.36	316.63	0.01 [−0.49, 0.52]
Random Effect Model Total (Z = 1.861, P = 0.063)							0.12 [−0.01, 0.24]

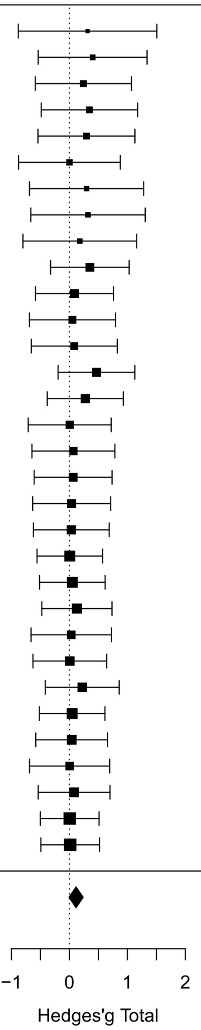

Supplementary Figure S10. Forest plot of RT effect moderator-analysis of localization derived from previous study.

Author(s) and Year	Experimental Total	Experimental Mean	Experimental SD	Control Total	Control Mean	Control SD	Hedges'g [95% CI]
Sakai et al. (2002)	6	−646.88	210.81	6	−651.44	186.39	0.02 [−1.11, 1.15]
Jackson et al. (2015)	15	1189.43	282.59	15	1134.04	302.41	0.18 [−0.54, 0.90]
Finocchiaro et al. (2015), Exp1	12	−1120.33	375.36	18	−1381.27	274.8	0.78 [0.02, 1.53]
Finocchiaro et al. (2015), Exp2	12	−1151.13	305.64	18	−1381.27	274.8	0.76 [0.00, 1.51]
Finocchiaro et al. (2015), Exp3	12	−1133.38	242.97	18	−1381.27	274.8	0.89 [0.13, 1.66]
Karabanov et al. (2015), Exp1	26	949	8060	26	861	9048	0.01 [−0.53, 0.55]
Karabanov et al. (2015), Exp2	26	−781	8970	26	−829	7384	0.01 [−0.54, 0.55]
Deschamps et al. (2020)	18	11.36	491.91	18	9.05	412.25	0.00 [−0.65, 0.66]
Jost et al. (2020)	22	1219	241	22	1178	239	0.16 [−0.43, 0.76]
Random Effect Model Total (Z = 1.46, P = 0.144)							0.24 [−0.08, 0.56]

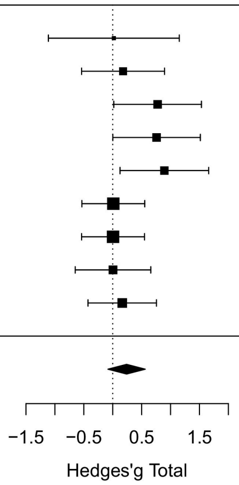

Supplementary Figure S11. Forest plot of RT effect moderator-analysis of localization derived from current study.

Author(s) and Year	Experimental Total	Experimental Mean	Experimental SD	Control Total	Control Mean	Control SD	Hedges'g [95% CI]
Sakai et al. (2002)	6	−646.88	210.81	6	−651.44	186.39	0.02 [−1.11, 1.15]
Finocchiaro et al. (2008)	6	−51	55.15	5	−87.5	132.38	0.31 [−0.88, 1.51]
Schuhmann et al. (2009)	10	475	53.82	8	454	38.05	0.40 [−0.54, 1.34]
Hartwigsen et al. (2010), Exp1	28	1070.67	1371.93	7	755.83	328.64	0.24 [−0.59, 1.07]
Hartwigsen et al. (2010), Exp2	28	1022.5	1024.46	7	686	113.5	0.35 [−0.49, 1.18]
Andoh et al. (2011)	10	962.5	462.63	10	962	433.3	0.00 [−0.88, 0.88]
Schuhmann et al. (2012), Exp1	8	471	51.89	8	455	44	0.30 [−0.69, 1.28]
Schuhmann et al. (2012), Exp2	8	488.2	40.01	8	474	39	0.32 [−0.67, 1.31]
Schuhmann et al. (2012), Exp3	8	477	39.61	8	470	29	0.18 [−0.80, 1.16]
Jackson et al. (2015)	15	1189.43	282.59	15	1134.04	302.41	0.18 [−0.54, 0.90]
Papeo et al. (2015), Exp1	14	−1033.7	261.45	14	−1045.67	163.54	0.05 [−0.69, 0.79]
Papeo et al. (2015), Exp2	14	1033.7	261.45	14	1008.9	293.48	0.08 [−0.66, 0.83]
Finocchiaro et al. (2015), Exp1	12	−1120.33	375.36	18	−1381.27	274.8	0.78 [0.02, 1.53]
Finocchiaro et al. (2015), Exp2	12	−1151.13	305.64	18	−1381.27	274.8	0.76 [0.00, 1.51]
Finocchiaro et al. (2015), Exp3	12	−1133.38	242.97	18	−1381.27	274.8	0.89 [0.13, 1.66]
Passeri et al. (2015), Exp1	18	578	23	18	567	22.02	0.47 [−0.20, 1.13]
Passeri et al. (2015), Exp2	18	574	26.62	18	567	22.02	0.27 [−0.38, 0.93]
Karabanov et al. (2015), Exp1	26	949	8060	26	861	9048	0.01 [−0.53, 0.55]
Karabanov et al. (2015), Exp2	26	−781	8970	26	−829	7384	0.01 [−0.54, 0.55]
Davey et al. (2015), Exp1	15	1293.67	451.24	15	1291.3	439.47	0.01 [−0.71, 0.72]
Davey et al. (2015), Exp2	15	−1259.2	439.12	15	−1291.3	439.47	0.07 [−0.65, 0.79]
Hartwigsen et al. (2016), Exp1	17	−973.16	700.55	17	−1021.29	727.83	0.06 [−0.61, 0.74]
Hartwigsen et al. (2016), Exp2	17	−1002.23	498.42	17	−1025.43	625.5	0.04 [−0.63, 0.71]
Woollamsa et al. (2017)	18	−548	72.28	18	−550.38	66.93	0.03 [−0.62, 0.69]
Klaus et al. (2018), Exp1	24	1789.5	432	24	1773	432	0.04 [−0.53, 0.60]
Klaus et al. (2018), Exp2	24	−1769.5	492.15	24	−1807	528	0.07 [−0.49, 0.64]
Zhang et al. (2018)	21	584.69	183.18	21	557.45	220.53	0.13 [−0.48, 0.73]
Ishkhanyan et al. (2020), Exp1	19	−559.33	116.35	19	−560.17	116.87	0.01 [−0.63, 0.64]
Ishkhanyan et al. (2020), Exp2	19	559	118.65	19	530.4	128.86	0.22 [−0.42, 0.86]
Piai et al. (2020)	24	−834.67	113.44	24	−840.17	112.87	0.05 [−0.52, 0.61]
Ward et al. (2022)	20	651.67	119.58	20	642	109.74	0.08 [−0.54, 0.70]
van der Burght et al. (2022), Exp1	30	−698.92	265.17	30	−715.04	242.57	0.06 [−0.44, 0.57]
van der Burght et al. (2022), Exp2	30	894.78	327.97	30	878.17	291.38	0.05 [−0.45, 0.56]
Random Effect Model Total (Z = 2.284, P = 0.022)							0.18 [0.03, 0.33]

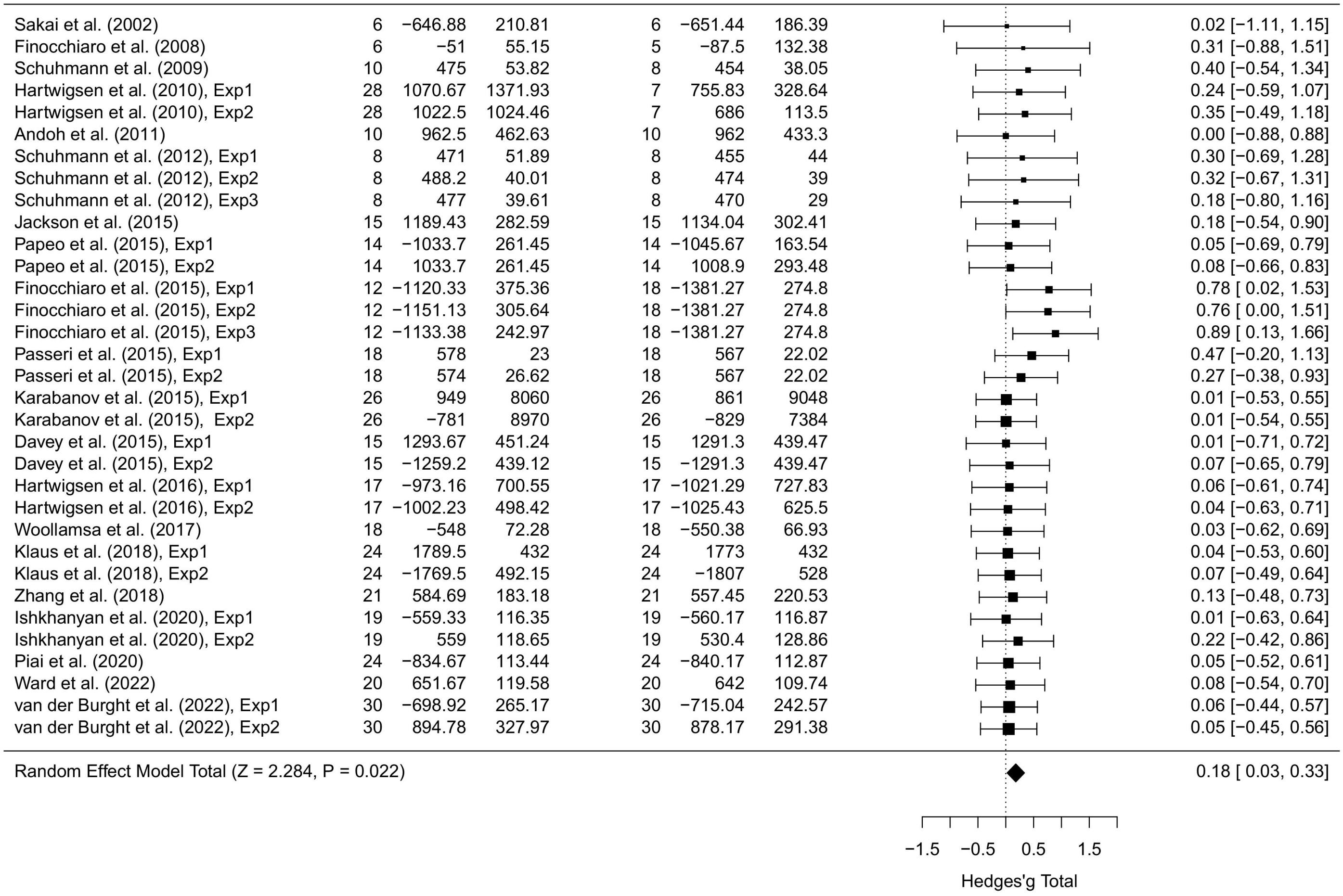

Supplementary Figure S12. Forest plot of RT effect moderator-analysis of rTMS

Author(s) and Year	Experimental Total	Experimental Mean	Experimental SD	Control Total	Control Mean	Control SD	Hedges'g [95% CI]
Willems et al. (2011)	18	721	184.42	8	662.77	187.73	0.29 [−0.54, 1.13]
Hartwigsena et al. (2013), Exp1	17	916.5	46.61	17	899.25	46.38	0.35 [−0.32, 1.03]
Hartwigsena et al. (2013), Exp2	17	916.5	46.61	17	912.25	44.85	0.09 [−0.58, 0.76]
Hartwigsen et al. (2016), Exp1	17	−962.97	717.11	17	−1021.2	748.62	0.08 [−0.60, 0.75]
Klaus et al. (2019)	16	−608	191.44	16	−614.5	198.27	0.03 [−0.66, 0.72]
Jost et al. (2020)	22	1219	241	22	1178	239	0.16 [−0.43, 0.76]
Sliwinska et al. (2021)	20	1286.5	188.3	20	1279	176.16	0.04 [−0.58, 0.66]
Random Effect Model Total (Z = 1.089, P = 0.276)							0.14 [−0.11, 0.39]

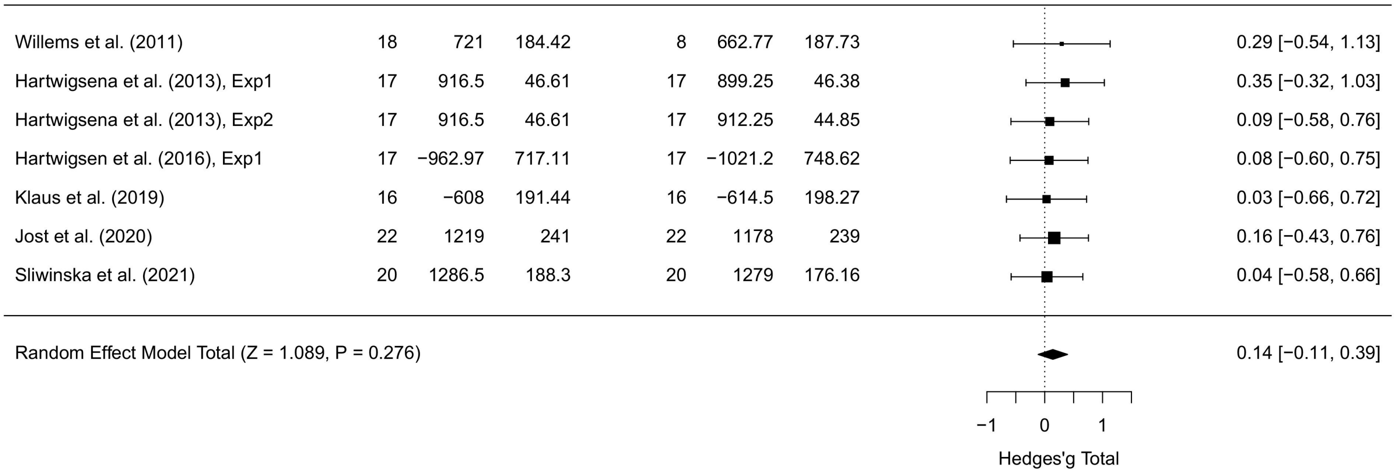

Supplementary Figure S13. Forest plot of RT effect moderator-analysis of TBS

Author(s) and Year	Experimental Total	Experimental Mean	Experimental SD	Control Total	Control Mean	Control SD	Hedges'g [95% CI]
Hartwigsena et al. (2013), Exp1	17	916.5	46.61	17	899.25	46.38	0.35 [−0.32, 1.03]
Hartwigsena et al. (2013), Exp2	17	916.5	46.61	17	912.25	44.85	0.09 [−0.58, 0.76]
Hartwigsen et al. (2016), Exp1	17	−962.97	717.11	17	−1021.2	748.62	0.08 [−0.60, 0.75]
Klaus et al. (2019)	16	−608	191.44	16	−614.5	198.27	0.03 [−0.66, 0.72]
Jost et al. (2020)	22	1219	241	22	1178	239	0.16 [−0.43, 0.76]
Sliwinska et al. (2021)	20	1286.5	188.3	20	1279	176.16	0.04 [−0.58, 0.66]
Random Effect Model Total (Z = 0.924, P = 0.356)							0.13 [−0.14, 0.39]

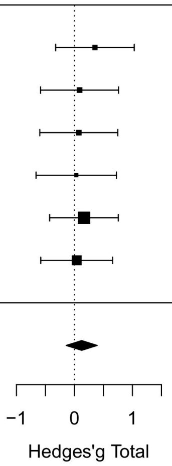

Supplementary Figure S14. Forest plot of RT effect moderator-analysis of cTBS

Author(s) and Year	Experimental Total	Experimental Mean	Experimental SD	Control Total	Control Mean	Control SD	Hedges'g [95% CI]
Hartwigsena et al. (2013), Exp1	17	916.5	46.61	17	899.25	46.38	0.35 [−0.32, 1.03]
Hartwigsena et al. (2013), Exp2	17	916.5	46.61	17	912.25	44.85	0.09 [−0.58, 0.76]
Klaus et al. (2019)	16	608	191.44	16	614.5	198.27	−0.03 [−0.72, 0.66]
Jost et al. (2020)	22	1219	241	22	1178	239	0.16 [−0.43, 0.76]
Sliwinska et al. (2021)	20	1286.5	188.3	20	1279	176.16	0.04 [−0.58, 0.66]
Random Effect Model Total (Z = 0.836, P = 0.403)							0.12 [−0.17, 0.41]

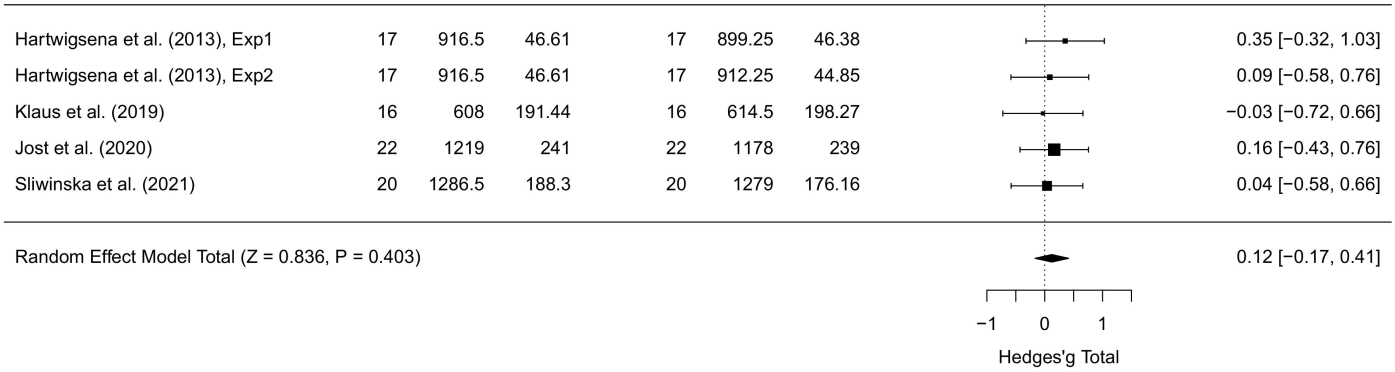

Supplementary Figure S15. Forest plot of supplementary RT effect moderator-analysis of cTBS

Author(s) and Year	Experimental Total	Experimental Mean	Experimental SD	Control Total	Control Mean	Control SD	Hedges'g [95% CI]
Sakai et al. (2002)	6	−646.88	210.81	6	−651.44	186.39	0.02 [−1.11, 1.15]
Schuhmann et al. (2009)	10	475	53.82	8	454	38.05	0.40 [−0.54, 1.34]
Hartwigsen et al. (2010), Exp2	28	1022.5	1024.46	7	686	113.5	0.35 [−0.49, 1.18]
Schuhmann et al. (2012), Exp1	8	471	51.89	8	455	44	0.30 [−0.69, 1.28]
Schuhmann et al. (2012), Exp2	8	488.2	40.01	8	474	39	0.32 [−0.67, 1.31]
Schuhmann et al. (2012), Exp3	8	477	39.61	8	470	29	0.18 [−0.80, 1.16]
Finocchiaro et al. (2015), Exp1	12	−1120.33	375.36	18	−1381.27	274.8	0.78 [0.02, 1.53]
Finocchiaro et al. (2015), Exp2	12	−1151.13	305.64	18	−1381.27	274.8	0.76 [0.00, 1.51]
Finocchiaro et al. (2015), Exp3	12	−1133.38	242.97	18	−1381.27	274.8	0.89 [0.13, 1.66]
Passeri et al. (2015), Exp1	18	578	23	18	567	22.02	0.47 [−0.20, 1.13]
Passeri et al. (2015), Exp2	18	574	26.62	18	567	22.02	0.27 [−0.38, 0.93]
Karabanov et al. (2015), Exp1	26	949	8060	26	861	9048	0.01 [−0.53, 0.55]
Karabanov et al. (2015), Exp2	26	−781	8970	26	−829	7384	0.01 [−0.54, 0.55]
Hartwigsen et al. (2016), Exp1	17	−973.16	700.55	17	−1021.29	727.83	0.06 [−0.61, 0.74]
Hartwigsen et al. (2016), Exp2	17	−1002.23	498.42	17	−1025.43	625.5	0.04 [−0.63, 0.71]
Klaus et al. (2018), Exp1	24	1789.5	432	24	1773	432	0.04 [−0.53, 0.60]
Klaus et al. (2018), Exp2	24	−1769.5	492.15	24	−1807	528	0.07 [−0.49, 0.64]
Zhang et al. (2018)	21	584.69	183.18	21	557.45	220.53	0.13 [−0.48, 0.73]
Ishkhanyan et al. (2020), Exp1	19	−559.33	116.35	19	−560.17	116.87	0.01 [−0.63, 0.64]
Ishkhanyan et al. (2020), Exp2	19	559	118.65	19	530.4	128.86	0.22 [−0.42, 0.86]
Deschamps et al. (2020)	18	11.36	491.91	18	9.05	412.25	0.00 [−0.65, 0.66]
Piai et al. (2020)	24	−834.67	113.44	24	−840.17	112.87	0.05 [−0.52, 0.61]
Finocchiaro et al. (2021)	16	−1742.12	1192.34	16	−1748	1142.47	0.00 [−0.69, 0.70]
Ward et al. (2022)	20	651.67	119.58	20	642	109.74	0.08 [−0.54, 0.70]
van der Burght et al. (2022), Exp1	30	−698.92	265.17	30	−715.04	242.57	0.06 [−0.44, 0.57]
van der Burght et al. (2022), Exp2	30	894.78	327.97	30	878.17	291.38	0.05 [−0.45, 0.56]
Random Effect Model Total (Z = 2.148, P = 0.032)							0.19 [0.02, 0.36]

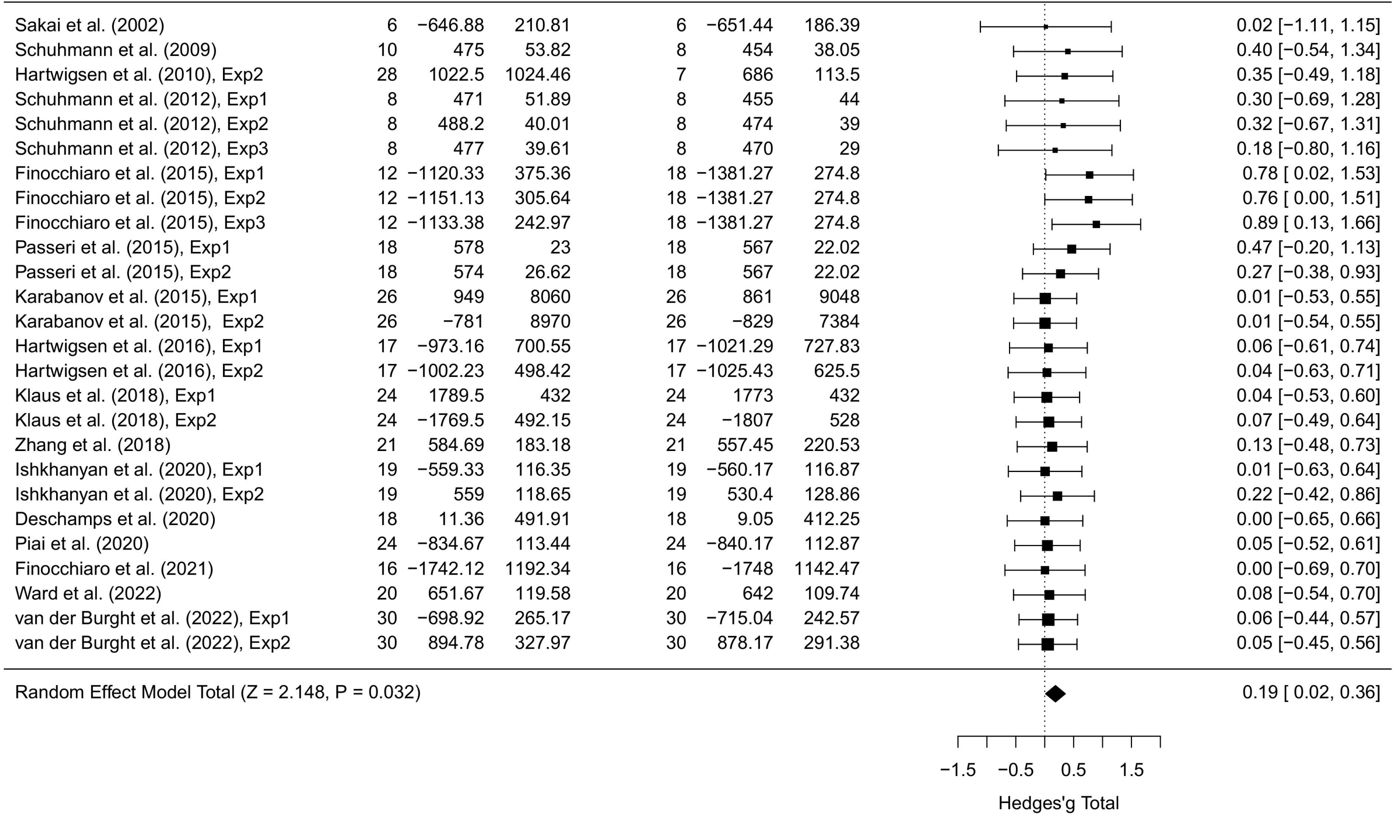

Supplementary Figure S16. Forest plot of RT effect moderator-analysis of online TMS

Author(s) and Year	Experimental Total	Experimental Mean	Experimental SD	Control Total	Control Mean	Control SD	Hedges'g [95% CI]
Finocchiaro et al. (2008)	6	−51	55.15	5	−87.5	132.38	0.31 [−0.88, 1.51]
Willems et al. (2011)	18	721	184.42	8	662.77	187.73	0.29 [−0.54, 1.13]
Andoh et al. (2011)	10	962.5	462.63	10	962	433.3	0.00 [−0.88, 0.88]
Hartwigsena et al. (2013), Exp1	17	916.5	46.61	17	899.25	46.38	0.35 [−0.32, 1.03]
Hartwigsena et al. (2013), Exp2	17	916.5	46.61	17	912.25	44.85	0.09 [−0.58, 0.76]
Jackson et al. (2015)	15	1189.43	282.59	15	1134.04	302.41	0.18 [−0.54, 0.90]
Papeo et al. (2015), Exp1	14	−1033.7	261.45	14	−1045.67	163.54	0.05 [−0.69, 0.79]
Papeo et al. (2015), Exp2	14	1033.7	261.45	14	1008.9	293.48	0.08 [−0.66, 0.83]
Davey et al. (2015), Exp1	15	1293.67	451.24	15	1291.3	439.47	0.01 [−0.71, 0.72]
Davey et al. (2015), Exp2	15	−1259.2	439.12	15	−1291.3	439.47	0.07 [−0.65, 0.79]
Hartwigsen et al. (2016), Exp1	17	−973.16	700.55	17	−1021.29	727.83	0.06 [−0.61, 0.74]
Hartwigsen et al. (2016), Exp2	17	−1002.23	498.42	17	−1025.43	625.5	0.04 [−0.63, 0.71]
Woollamsa et al. (2017)	18	−548	72.28	18	−550.38	66.93	0.03 [−0.62, 0.69]
Klaus et al. (2019)	16	−608	191.44	16	−614.5	198.27	0.03 [−0.66, 0.72]
Jost et al. (2020)	22	1219	241	22	1178	239	0.16 [−0.43, 0.76]
Sliwinska et al. (2021)	20	1286.5	188.3	20	1279	176.16	0.04 [−0.58, 0.66]
Random Effect Model Total (Z = 1.147, P = 0.251)							0.10 [−0.07, 0.28]

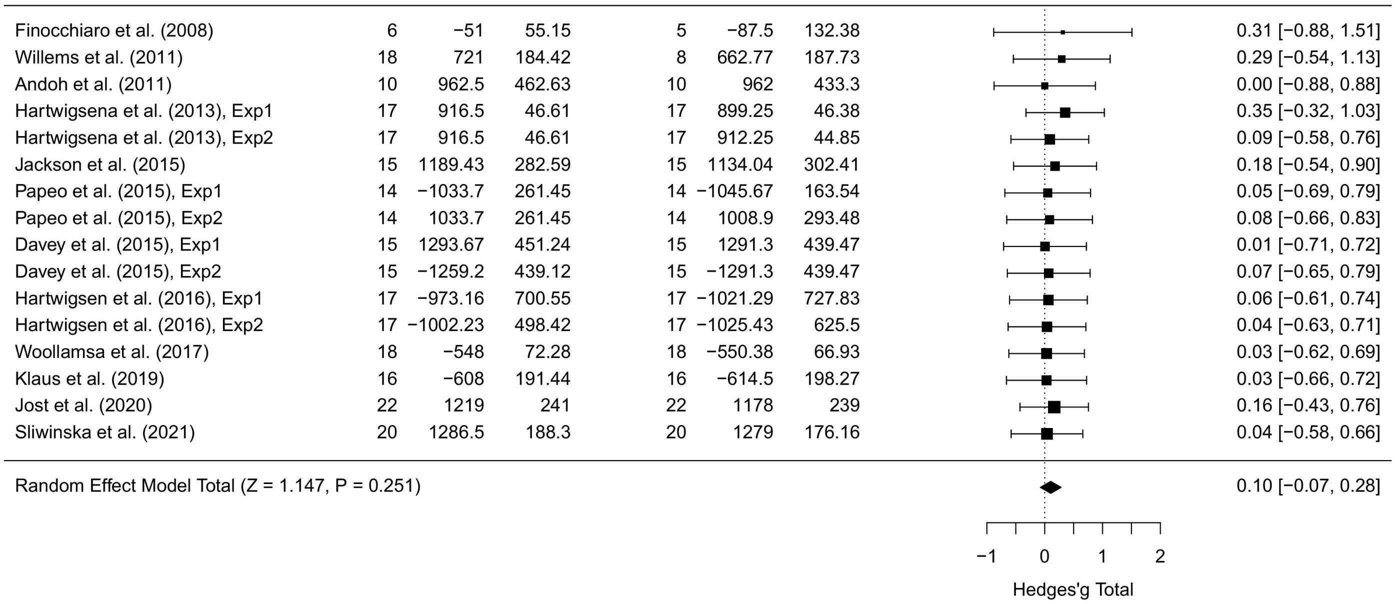

Supplementary Figure S17. Forest plot of RT effect moderator-analysis of offline TMS

Author(s) and Year	Experimental Total	Experimental Mean	Experimental SD	Control Total	Control Mean	Control SD	Hedges'g [95% CI]
Schuhmann et al. (2009)	10	475	53.82	8	454	38.05	0.40 [−0.54, 1.34]
Hartwigsen et al. (2010), Exp1	28	1070.67	1371.93	7	755.83	328.64	0.24 [−0.59, 1.07]
Hartwigsen et al. (2010), Exp2	28	1022.5	1024.46	7	686	113.5	0.35 [−0.49, 1.18]
Willems et al. (2011)	18	721	184.42	8	662.77	187.73	0.29 [−0.54, 1.13]
Andoh et al. (2011)	10	962.5	462.63	10	962	433.3	0.00 [−0.88, 0.88]
Schuhmann et al. (2012), Exp1	8	471	51.89	8	455	44	0.30 [−0.69, 1.28]
Schuhmann et al. (2012), Exp2	8	488.2	40.01	8	474	39	0.32 [−0.67, 1.31]
Schuhmann et al. (2012), Exp3	8	477	39.61	8	470	29	0.18 [−0.80, 1.16]
Hartwigsena et al. (2013), Exp1	17	916.5	46.61	17	899.25	46.38	0.35 [−0.32, 1.03]
Hartwigsena et al. (2013), Exp2	17	916.5	46.61	17	912.25	44.85	0.09 [−0.58, 0.76]
Finocchiaro et al. (2015), Exp1	12	−1120.33	375.36	18	−1381.27	274.8	0.78 [0.02, 1.53]
Finocchiaro et al. (2015), Exp2	12	−1151.13	305.64	18	−1381.27	274.8	0.76 [0.00, 1.51]
Finocchiaro et al. (2015), Exp3	12	−1133.38	242.97	18	−1381.27	274.8	0.89 [0.13, 1.66]
Passeri et al. (2015), Exp1	18	578	23	18	567	22.02	0.47 [−0.20, 1.13]
Passeri et al. (2015), Exp2	18	574	26.62	18	567	22.02	0.27 [−0.38, 0.93]
Karabanov et al. (2015), Exp1	26	949	8060	26	861	9048	0.01 [−0.53, 0.55]
Karabanov et al. (2015), Exp2	26	−781	8970	26	−829	7384	0.01 [−0.54, 0.55]
Hartwigsen et al. (2016), Exp1	17	−973.16	700.55	17	−1021.29	727.83	0.06 [−0.61, 0.74]
Hartwigsen et al. (2016), Exp2	17	−1002.23	498.42	17	−1025.43	625.5	0.04 [−0.63, 0.71]
Klaus et al. (2018), Exp1	24	1789.5	432	24	1773	432	0.04 [−0.53, 0.60]
Klaus et al. (2018), Exp2	24	−1769.5	492.15	24	−1807	528	0.07 [−0.49, 0.64]
Zhang et al. (2018)	21	584.69	183.18	21	557.45	220.53	0.13 [−0.48, 0.73]
Klaus et al. (2019)	16	−608	191.44	16	−614.5	198.27	0.03 [−0.66, 0.72]
Ishkhanyan et al. (2020), Exp1	19	−559.33	116.35	19	−560.17	116.87	0.01 [−0.63, 0.64]
Ishkhanyan et al. (2020), Exp2	19	559	118.65	19	530.4	128.86	0.22 [−0.42, 0.86]
Jost et al. (2020)	22	1219	241	22	1178	239	0.16 [−0.43, 0.76]
Piai et al. (2020)	24	−834.67	113.44	24	−840.17	112.87	0.05 [−0.52, 0.61]
Sliwinska et al. (2021)	20	1286.5	188.3	20	1279	176.16	0.04 [−0.58, 0.66]
Ward et al. (2022)	20	651.67	119.58	20	642	109.74	0.08 [−0.54, 0.70]
van der Burght et al. (2022), Exp1	30	−698.92	265.17	30	−715.04	242.57	0.06 [−0.44, 0.57]
van der Burght et al. (2022), Exp2	30	894.78	327.97	30	878.17	291.38	0.05 [−0.45, 0.56]
Random Effect Model Total (Z = 2.353, P = 0.019)							0.21 [0.03, 0.38]

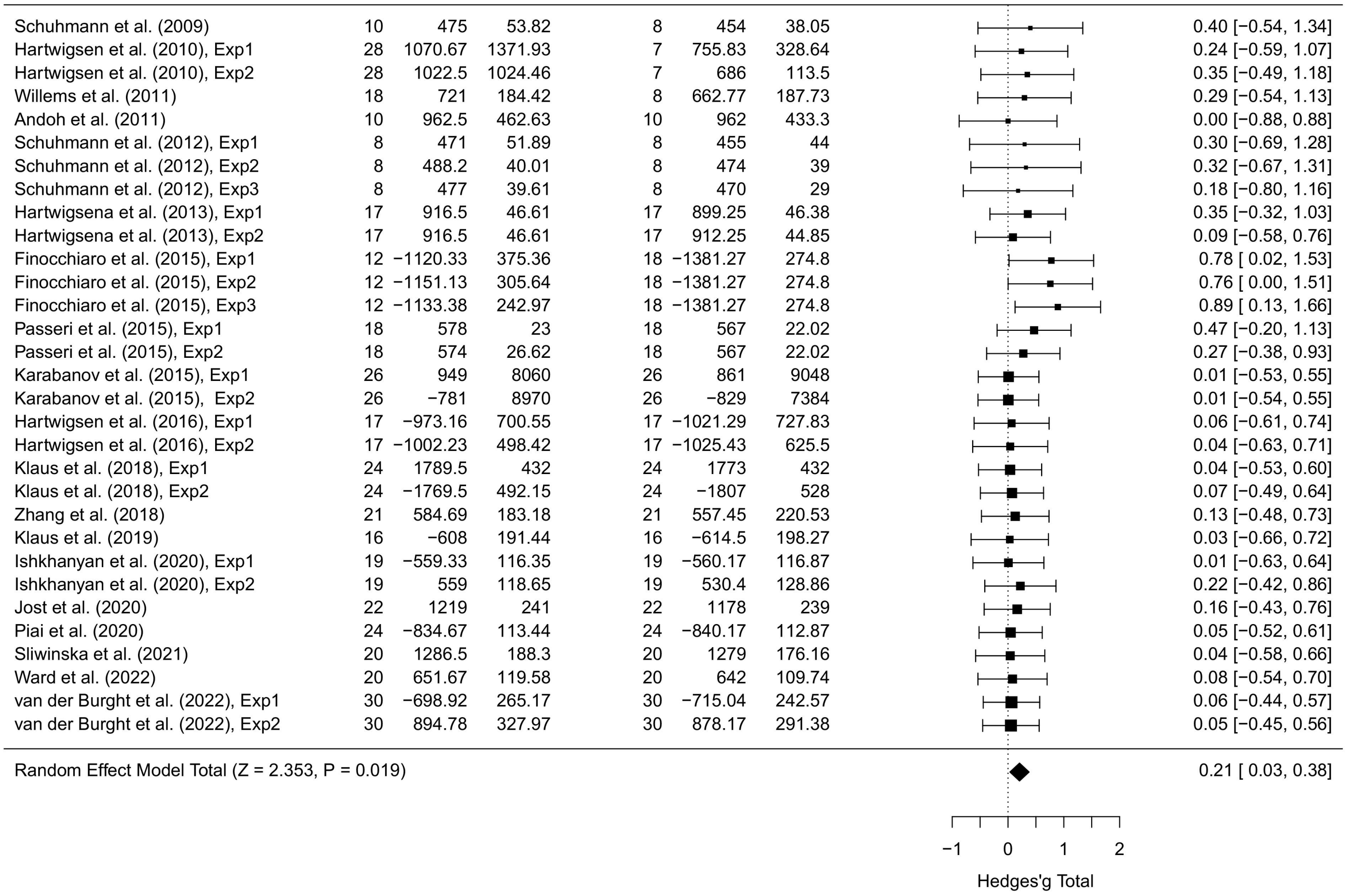

Supplementary Figure S18. Forest plot of RT effect moderator-analysis of high frequency TMS

Author(s) and Year	Experimental Total	Experimental Mean	Experimental SD	Control Total	Control Mean	Control SD	Hedges'g [95% CI]
Finocchiaro et al. (2008)	6	−51	55.15	5	−87.5	132.38	0.31 [−0.88, 1.51]
Jackson et al. (2015)	15	1189.43	282.59	15	1134.04	302.41	0.18 [−0.54, 0.90]
Papeo et al. (2015), Exp1	14	−1033.7	261.45	14	−1045.67	163.54	0.05 [−0.69, 0.79]
Papeo et al. (2015), Exp2	14	1033.7	261.45	14	1008.9	293.48	0.08 [−0.66, 0.83]
Davey et al. (2015), Exp1	15	1293.67	451.24	15	1291.3	439.47	0.01 [−0.71, 0.72]
Davey et al. (2015), Exp2	15	−1259.2	439.12	15	−1291.3	439.47	0.07 [−0.65, 0.79]
Woollamsa et al. (2017)	18	−548	72.28	18	−550.38	66.93	0.03 [−0.62, 0.69]
Random Effect Model Total (Z = 0.574, P = 0.566)							0.08 [−0.20, 0.37]

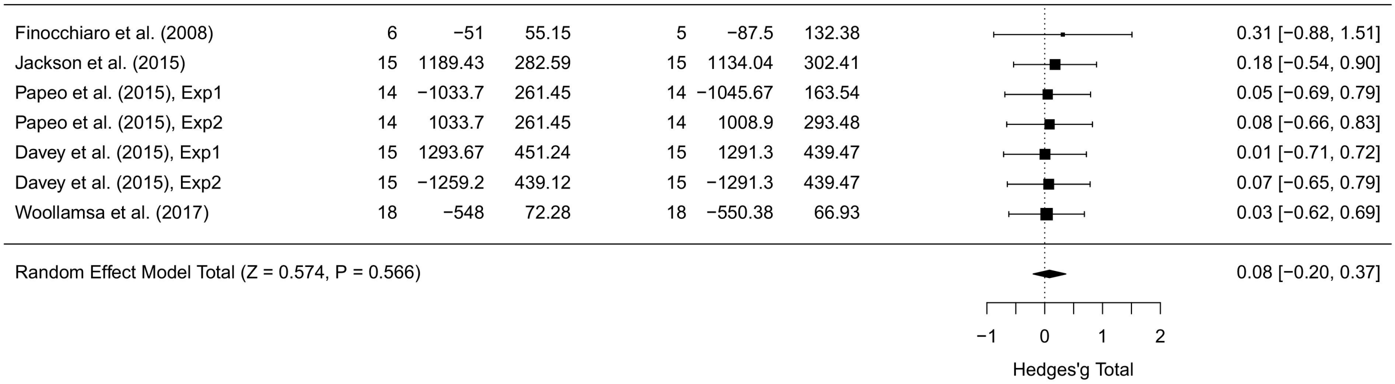

Supplementary Figure S19. Forest plot of RT effect moderator-analysis of low frequency TMS

Author(s) and Year	Experimental Total	Experimental Mean	Experimental SD	Control Total	Control Mean	Control SD	Hedges'g [95% CI]
Schuhmann et al. (2009)	10	475	53.82	8	454	38.05	0.40 [−0.54, 1.34]
Hartwigsen et al. (2010), Exp1	28	1070.67	1371.93	7	755.83	328.64	0.24 [−0.59, 1.07]
Hartwigsen et al. (2010), Exp2	28	1022.5	1024.46	7	686	113.5	0.35 [−0.49, 1.18]
Willems et al. (2011)	18	721	184.42	8	662.77	187.73	0.29 [−0.54, 1.13]
Andoh et al. (2011)	10	962.5	462.63	10	962	433.3	0.00 [−0.88, 0.88]
Schuhmann et al. (2012), Exp1	8	471	51.89	8	455	44	0.30 [−0.69, 1.28]
Schuhmann et al. (2012), Exp2	8	488.2	40.01	8	474	39	0.32 [−0.67, 1.31]
Schuhmann et al. (2012), Exp3	8	477	39.61	8	470	29	0.18 [−0.80, 1.16]
Hartwigsena et al. (2013), Exp1	17	916.5	46.61	17	899.25	46.38	0.35 [−0.32, 1.03]
Hartwigsena et al. (2013), Exp2	17	916.5	46.61	17	912.25	44.85	0.09 [−0.58, 0.76]
Finocchiaro et al. (2015), Exp1	12	1120.33	375.36	18	1381.27	274.8	−0.78 [−1.53, −0.02]
Finocchiaro et al. (2015), Exp2	12	1151.13	305.64	18	1381.27	274.8	−0.76 [−1.51, −0.00]
Finocchiaro et al. (2015), Exp3	12	1133.38	242.97	18	1381.27	274.8	−0.89 [−1.66, −0.13]
Passeri et al. (2015), Exp1	18	578	23	18	567	22.02	0.47 [−0.20, 1.13]
Passeri et al. (2015), Exp2	18	574	26.62	18	567	22.02	0.27 [−0.38, 0.93]
Karabanov et al. (2015), Exp1	26	949	8060	26	861	9048	0.01 [−0.53, 0.55]
Karabanov et al. (2015), Exp2	26	781	8970	26	829	7384	−0.01 [−0.55, 0.54]
Hartwigsen et al. (2016), Exp1	17	973.16	700.55	17	1021.29	727.83	−0.06 [−0.74, 0.61]
Hartwigsen et al. (2016), Exp2	17	1002.23	498.42	17	1025.43	625.5	−0.04 [−0.71, 0.63]
Klaus et al. (2018), Exp1	24	1789.5	432	24	1773	432	0.04 [−0.53, 0.60]
Klaus et al. (2018), Exp2	24	1769.5	492.15	24	1807	528	−0.07 [−0.64, 0.49]
Zhang et al. (2018)	21	584.69	183.18	21	557.45	220.53	0.13 [−0.48, 0.73]
Klaus et al. (2019)	16	608	191.44	16	614.5	198.27	−0.03 [−0.72, 0.66]
Ishkhanyan et al. (2020), Exp1	19	559.33	116.35	19	560.17	116.87	−0.01 [−0.64, 0.63]
Ishkhanyan et al. (2020), Exp2	19	559	118.65	19	530.4	128.86	0.22 [−0.42, 0.86]
Jost et al. (2020)	22	1219	241	22	1178	239	0.16 [−0.43, 0.76]
Piai et al. (2020)	24	834.67	113.44	24	840.17	112.87	−0.05 [−0.61, 0.52]
Sliwinska et al. (2021)	20	1286.5	188.3	20	1279	176.16	0.04 [−0.58, 0.66]
Ward et al. (2022)	20	651.67	119.58	20	642	109.74	0.08 [−0.54, 0.70]
van der Burght et al. (2022), Exp1	30	698.92	265.17	30	715.04	242.57	−0.06 [−0.57, 0.44]
van der Burght et al. (2022), Exp2	30	894.78	327.97	30	878.17	291.38	0.05 [−0.45, 0.56]
Random Effect Model Total (Z = −0.054, P = 0.957)							−0.01 [−0.36, 0.34]

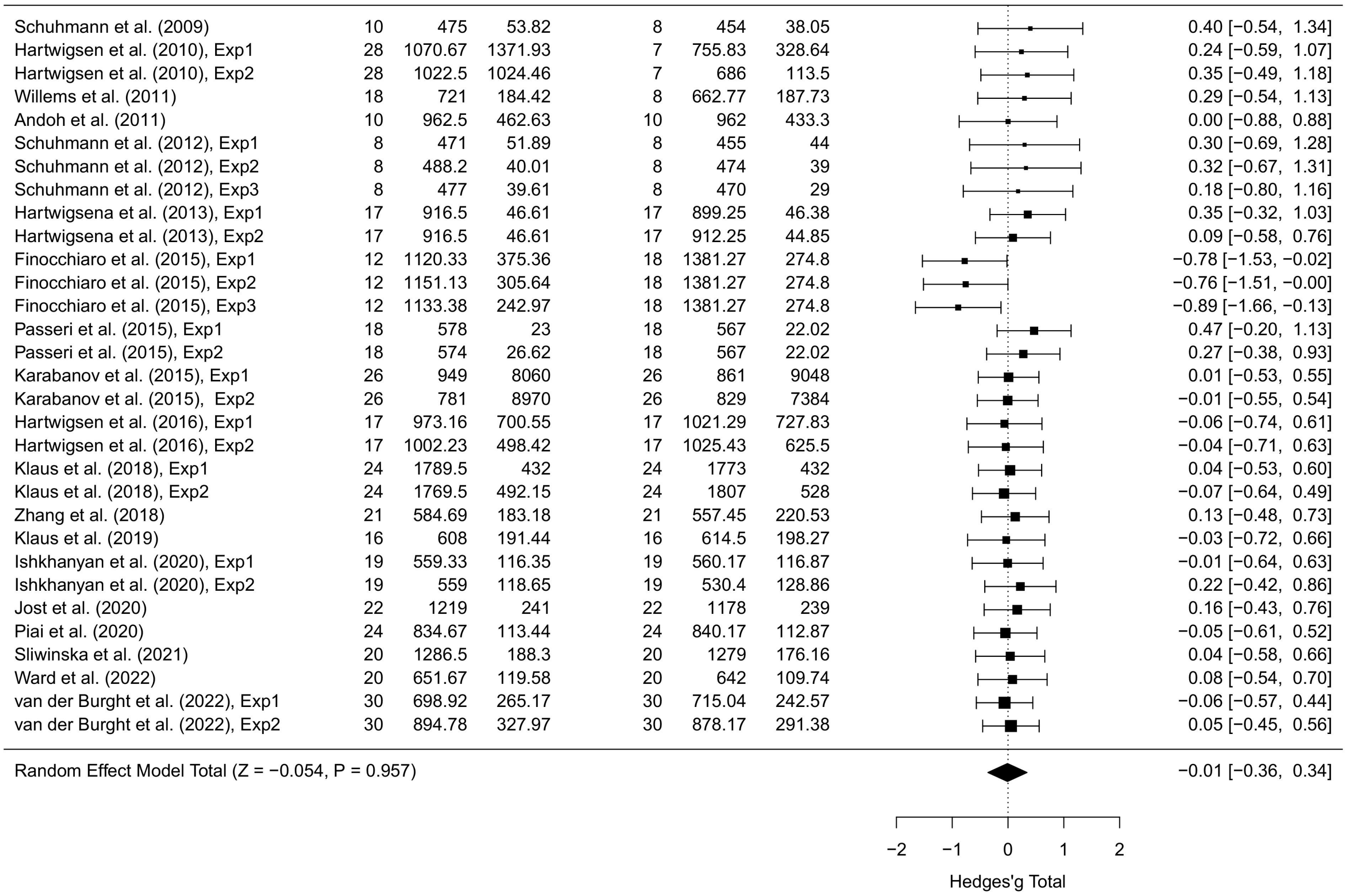

***Supplementary Figure S20.** Forest plot of RT effect moderator-analysis with original values of high frequency TMS

Author(s) and Year	Experimental Total	Experimental Mean	Experimental SD	Control Total	Control Mean	Control SD	Hedges'g [95% CI]
Finocchiaro et al. (2008)	6	51	55.15	5	87.5	132.38	−0.31 [−1.51, 0.88]
Jackson et al. (2015)	15	1189.43	282.59	15	1134.04	302.41	0.18 [−0.54, 0.90]
Papeo et al. (2015), Exp1	14	1033.7	261.45	14	1045.67	163.54	−0.05 [−0.79, 0.69]
Papeo et al. (2015), Exp2	14	1033.7	261.45	14	1008.9	293.48	0.08 [−0.66, 0.83]
Davey et al. (2015), Exp1	15	1293.67	451.24	15	1291.3	439.47	0.01 [−0.71, 0.72]
Davey et al. (2015), Exp2	15	1259.2	439.12	15	1291.3	439.47	−0.07 [−0.79, 0.65]
Woollamsa et al. (2017)	18	548	72.28	18	550.38	66.93	−0.03 [−0.69, 0.62]
Random Effect Model Total (Z = −0.007, P = 0.994)							−0.00 [−0.28, 0.28]

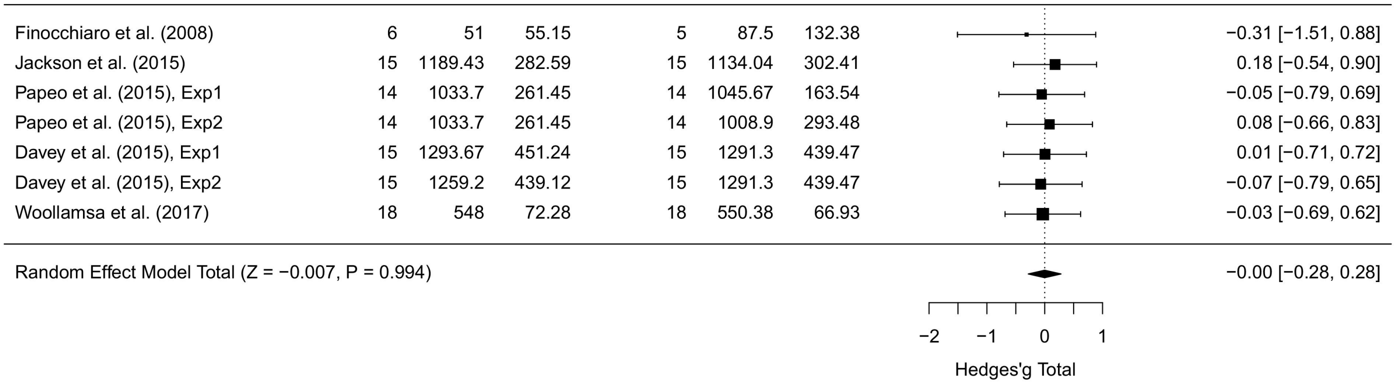

***Supplementary Figure S21.** Forest plot of RT effect moderator-analysis with original values of low frequency TMS

Author(s) and Year	Experimental Total	Experimental Mean	Experimental SD	Control Total	Control Mean	Control SD	Hedges'g [95% CI]
Hartwigsen et al. (2010), Exp1	28	1070.67	1371.93	7	755.83	328.64	0.24 [−0.59, 1.07]
Hartwigsen et al. (2010), Exp2	28	1022.5	1024.46	7	686	113.5	0.35 [−0.49, 1.18]
Hartwigsena et al. (2013), Exp1	17	916.5	46.61	17	899.25	46.38	0.35 [−0.32, 1.03]
Hartwigsena et al. (2013), Exp2	17	916.5	46.61	17	912.25	44.85	0.09 [−0.58, 0.76]
Random Effect Model Total (Z = 1.318, P = 0.188)							0.25 [−0.12, 0.62]

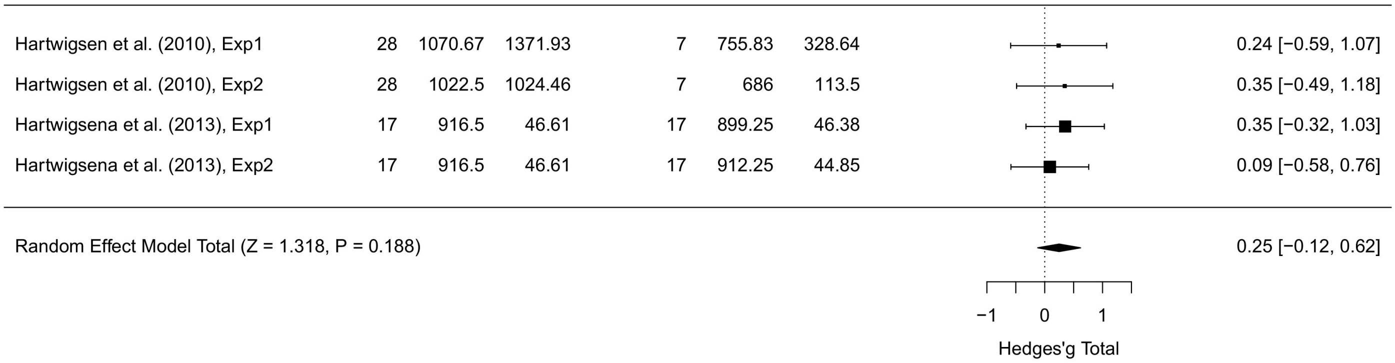

Supplementary Figure S22. Forest plot of RT effect moderator-analysis of AMT (a type of intensity)

Author(s) and Year	Experimental Total	Experimental Mean	Experimental SD	Control Total	Control Mean	Control SD	Hedges'g [95% CI]
Finocchiaro et al. (2008)	6	−51	55.15	5	−87.5	132.38	0.31 [−0.88, 1.51]
Schuhmann et al. (2009)	10	475	53.82	8	454	38.05	0.40 [−0.54, 1.34]
Schuhmann et al. (2012), Exp1	8	471	51.89	8	455	44	0.30 [−0.69, 1.28]
Schuhmann et al. (2012), Exp2	8	488.2	40.01	8	474	39	0.32 [−0.67, 1.31]
Schuhmann et al. (2012), Exp3	8	477	39.61	8	470	29	0.18 [−0.80, 1.16]
Finocchiaro et al. (2015), Exp1	12	−1120.33	375.36	18	−1381.27	274.8	0.78 [0.02, 1.53]
Finocchiaro et al. (2015), Exp2	12	−1151.13	305.64	18	−1381.27	274.8	0.76 [0.00, 1.51]
Finocchiaro et al. (2015), Exp3	12	−1133.38	242.97	18	−1381.27	274.8	0.89 [0.13, 1.66]
Karabanov et al. (2015), Exp1	26	949	8060	26	861	9048	0.01 [−0.53, 0.55]
Karabanov et al. (2015), Exp2	26	−781	8970	26	−829	7384	0.01 [−0.54, 0.55]
Hartwigsen et al. (2016), Exp1	17	−973.16	700.55	17	−1021.29	727.83	0.06 [−0.61, 0.74]
Hartwigsen et al. (2016), Exp2	17	−1002.23	498.42	17	−1025.43	625.5	0.04 [−0.63, 0.71]
Klaus et al. (2019)	16	−608	191.44	16	−614.5	198.27	0.03 [−0.66, 0.72]
Ishkhanyan et al. (2020), Exp1	19	−559.33	116.35	19	−560.17	116.87	0.01 [−0.63, 0.64]
Ishkhanyan et al. (2020), Exp2	19	559	118.65	19	530.4	128.86	0.22 [−0.42, 0.86]
Deschamps et al. (2020)	18	11.36	491.91	18	9.05	412.25	0.00 [−0.65, 0.66]
Jost et al. (2020)	22	1219	241	22	1178	239	0.16 [−0.43, 0.76]
Piai et al. (2020)	24	−834.67	113.44	24	−840.17	112.87	0.05 [−0.52, 0.61]
van der Burght et al. (2022), Exp1	30	−698.92	265.17	30	−715.04	242.57	0.06 [−0.44, 0.57]
van der Burght et al. (2022), Exp2	30	894.78	327.97	30	878.17	291.38	0.05 [−0.45, 0.56]
Random Effect Model Total (Z = 1.609, P = 0.108)							0.20 [−0.04, 0.45]

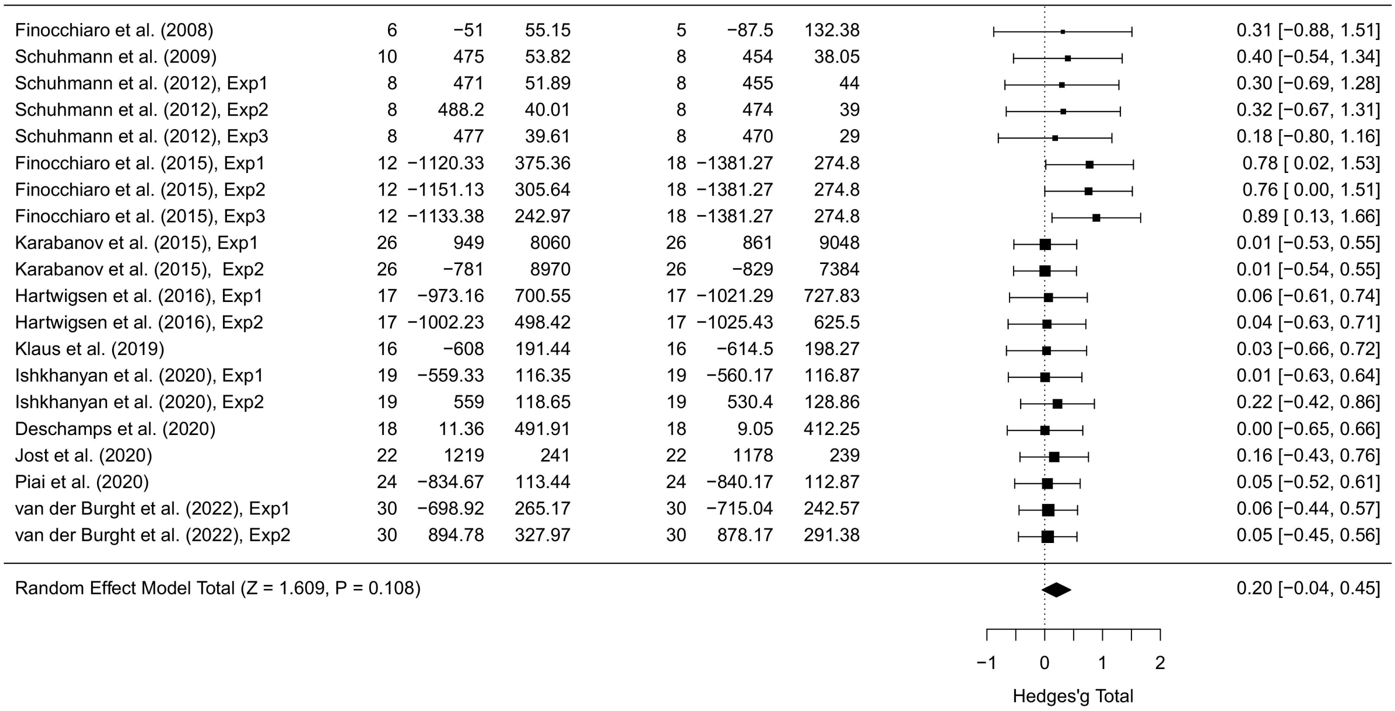

Supplementary Figure S23. Forest plot of RT effect moderator-analysis of RMT (a type of intensity)

Author(s) and Year	Experimental Total	Experimental Mean	Experimental SD	Control Total	Control Mean	Control SD	Hedges'g [95% CI]
Sakai et al. (2002)	6	−646.88	210.81	6	−651.44	186.39	0.02 [−1.11, 1.15]
Finocchiaro et al. (2008)	6	−51	55.15	5	−87.5	132.38	0.31 [−0.88, 1.51]
Schuhmann et al. (2009)	10	475	53.82	8	454	38.05	0.40 [−0.54, 1.34]
Hartwigsen et al. (2010), Exp1	28	1070.67	1371.93	7	755.83	328.64	0.24 [−0.59, 1.07]
Hartwigsen et al. (2010), Exp2	28	1022.5	1024.46	7	686	113.5	0.35 [−0.49, 1.18]
Passeri et al. (2015), Exp1	18	578	23	18	567	22.02	0.47 [−0.20, 1.13]
Passeri et al. (2015), Exp2	18	574	26.62	18	567	22.02	0.27 [−0.38, 0.93]
Hartwigsen et al. (2016), Exp1	17	−973.16	700.55	17	−1021.29	727.83	0.06 [−0.61, 0.74]
Zhang et al. (2018)	21	584.69	183.18	21	557.45	220.53	0.13 [−0.48, 0.73]
Klaus et al. (2019)	16	−608	191.44	16	−614.5	198.27	0.03 [−0.66, 0.72]
Ishkhanyan et al. (2020), Exp1	19	−559.33	116.35	19	−560.17	116.87	0.01 [−0.63, 0.64]
Ishkhanyan et al. (2020), Exp2	19	559	118.65	19	530.4	128.86	0.22 [−0.42, 0.86]
Jost et al. (2020)	22	1219	241	22	1178	239	0.16 [−0.43, 0.76]
Finocchiaro et al. (2021)	16	−1742.12	1192.34	16	−1748	1142.47	0.00 [−0.69, 0.70]
Ward et al. (2022)	20	651.67	119.58	20	642	109.74	0.08 [−0.54, 0.70]
Random Effect Model Total (Z = 1.819, P = 0.069)							0.17 [−0.01, 0.35]

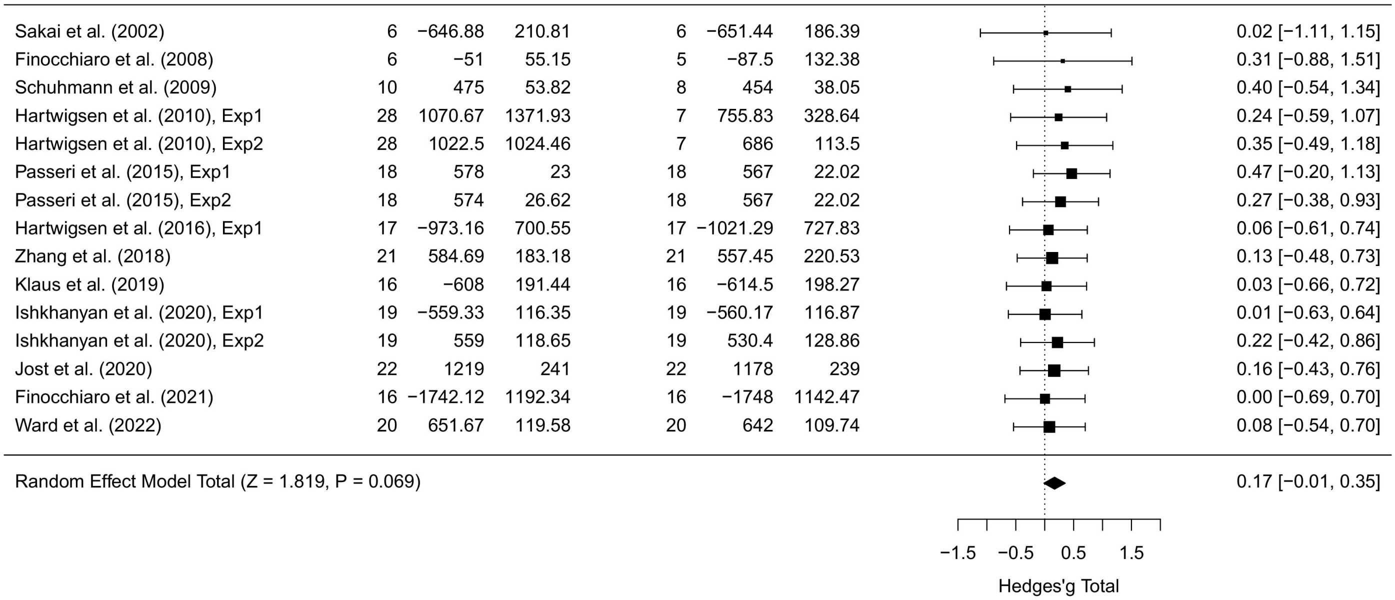

Supplementary Figure S24. Forest plot of RT effect moderator-analysis of sham TMS (a type of control conditions)

Author(s) and Year	Experimental Total	Experimental Mean	Experimental SD	Control Total	Control Mean	Control SD	Hedges'g [95% CI]
Andoh et al. (2011)	10	962.5	462.63	10	962	433.3	0.00 [−0.88, 0.88]
Schuhmann et al. (2012), Exp1	8	471	51.89	8	455	44	0.30 [−0.69, 1.28]
Schuhmann et al. (2012), Exp2	8	488.2	40.01	8	474	39	0.32 [−0.67, 1.31]
Schuhmann et al. (2012), Exp3	8	477	39.61	8	470	29	0.18 [−0.80, 1.16]
Papeo et al. (2015), Exp1	14	−1033.7	261.45	14	−1045.67	163.54	0.05 [−0.69, 0.79]
Finocchiaro et al. (2015), Exp1	12	−1120.33	375.36	18	−1381.27	274.8	0.78 [0.02, 1.53]
Finocchiaro et al. (2015), Exp2	12	−1151.13	305.64	18	−1381.27	274.8	0.76 [0.00, 1.51]
Finocchiaro et al. (2015), Exp3	12	−1133.38	242.97	18	−1381.27	274.8	0.89 [0.13, 1.66]
Woollamsa et al. (2017)	18	−548	72.28	18	−550.38	66.93	0.03 [−0.62, 0.69]
Random Effect Model Total (Z = 1.661, P = 0.097)							0.31 [−0.06, 0.67]

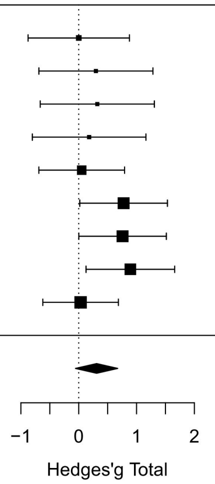

Supplementary Figure S25. Forest plot of RT effect moderator-analysis of no TMS (a type of control conditions)

Author(s) and Year	Experimental Total	Experimental Mean	Experimental SD	Control Total	Control Mean	Control SD	Hedges'g [95% CI]
Passeri et al. (2015), Exp1	18	578	23	18	567	22.02	0.47 [−0.20, 1.13]
Passeri et al. (2015), Exp2	18	574	26.62	18	567	22.02	0.27 [−0.38, 0.93]
Davey et al. (2015), Exp1	15	1293.67	451.24	15	1291.3	439.47	0.01 [−0.71, 0.72]
Davey et al. (2015), Exp2	15	−1259.2	439.12	15	−1291.3	439.47	0.07 [−0.65, 0.79]
Klaus et al. (2018), Exp1	24	1789.5	432	24	1773	432	0.04 [−0.53, 0.60]
Klaus et al. (2018), Exp2	24	−1769.5	492.15	24	−1807	528	0.07 [−0.49, 0.64]
Piai et al. (2020)	24	−834.67	113.44	24	−840.17	112.87	0.05 [−0.52, 0.61]
van der Burght et al. (2022), Exp1	30	−698.92	265.17	30	−715.04	242.57	0.06 [−0.44, 0.57]
van der Burght et al. (2022), Exp2	30	894.78	327.97	30	878.17	291.38	0.05 [−0.45, 0.56]
Random Effect Model Total (Z = 1.073, P = 0.283)							0.11 [−0.09, 0.31]

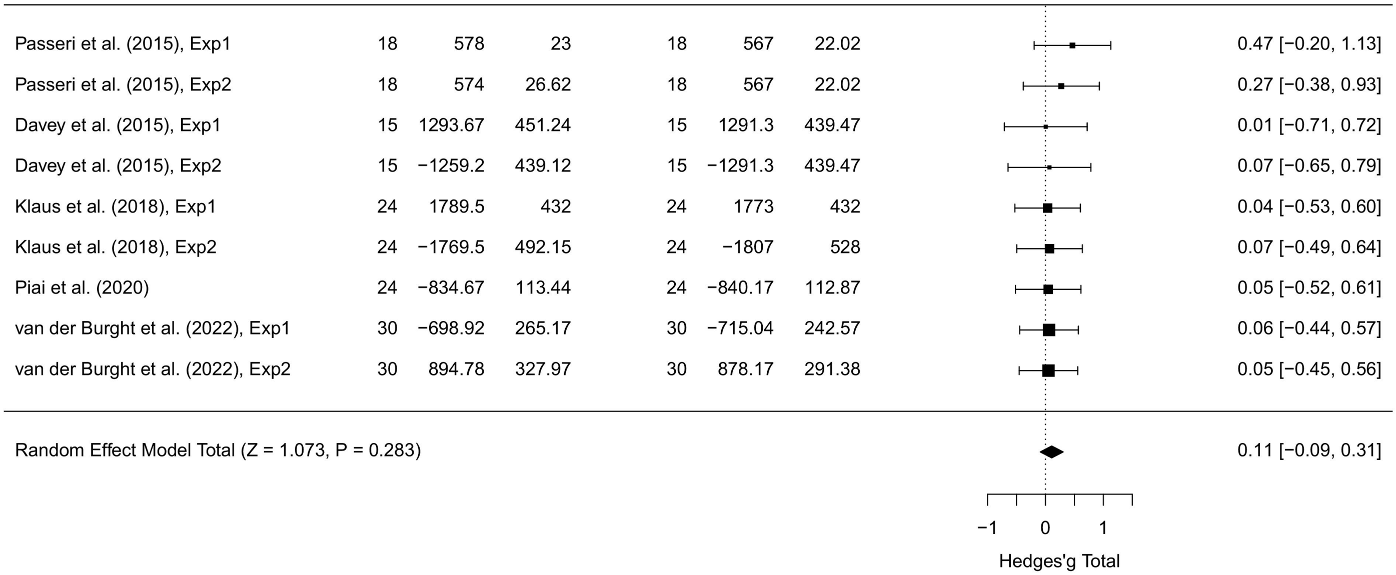

Supplementary Figure S26. Forest plot of RT effect moderator-analysis of vertex (a type of control conditions)

Author(s) and Year	Experimental Total	Experimental Mean	Experimental SD	Control Total	Control Mean	Control SD	Hedges'g [95% CI]
Finocchiaro et al. (2008)	6	−51	55.15	5	−87.5	132.38	0.31 [−0.88, 1.51]
Papeo et al. (2015), Exp2	14	1033.7	261.45	14	1008.9	293.48	0.08 [−0.66, 0.83]
Karabanov et al. (2015), Exp1	26	949	8060	26	861	9048	0.01 [−0.53, 0.55]
Karabanov et al. (2015), Exp2	26	−781	8970	26	−829	7384	0.01 [−0.54, 0.55]
Sliwinska et al. (2021)	20	1286.5	188.3	20	1279	176.16	0.04 [−0.58, 0.66]
Random Effect Model Total (Z = 0.3, P = 0.764)							0.04 [−0.25, 0.33]

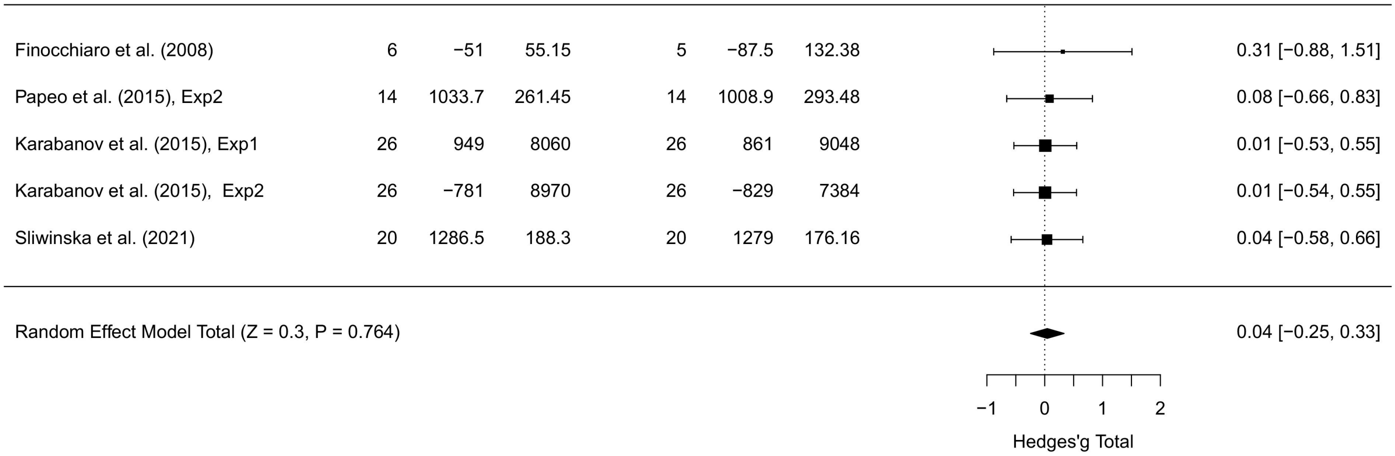

Supplementary Figure S27. Forest plot of RT effect moderator-analysis of other brain region (a type of control conditions)

Author(s) and Year	Experimental Total	Experimental Mean	Experimental SD	Control Total	Control Mean	Control SD	Hedges'g [95% CI]
Willems et al. (2011)	18	721	184.42	8	662.77	187.73	0.29 [−0.54, 1.13]
Hartwigsena et al. (2013), Exp1	17	916.5	46.61	17	899.25	46.38	0.35 [−0.32, 1.03]
Hartwigsena et al. (2013), Exp2	17	916.5	46.61	17	912.25	44.85	0.09 [−0.58, 0.76]
Jackson et al. (2015)	15	1189.43	282.59	15	1134.04	302.41	0.18 [−0.54, 0.90]
Hartwigsen et al. (2016), Exp2	17	−1002.23	498.42	17	−1025.43	625.5	0.04 [−0.63, 0.71]
Deschamps et al. (2020)	18	11.36	491.91	18	9.05	412.25	0.00 [−0.65, 0.66]
Random Effect Model Total (Z = 1.023, P = 0.306)							0.15 [−0.14, 0.43]

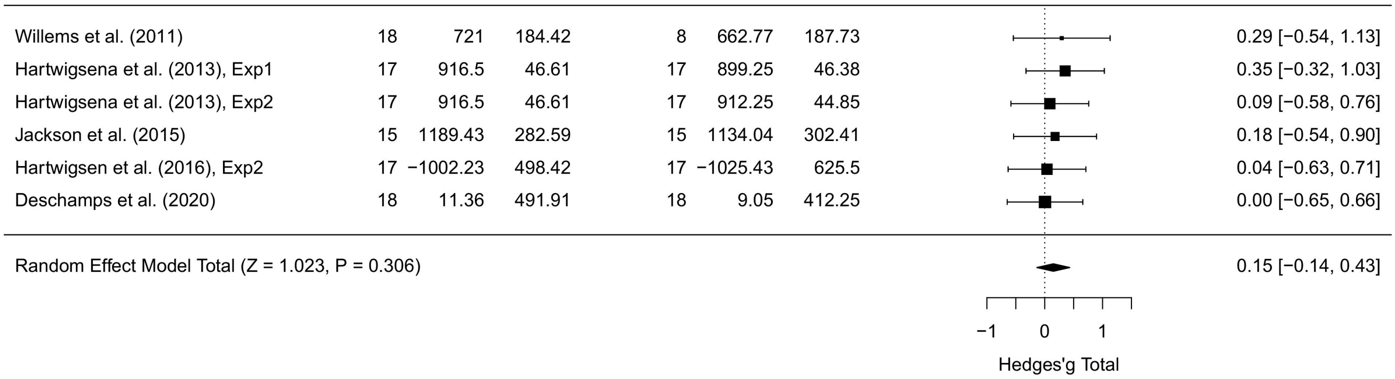

Supplementary Figure S28. Forest plot of RT effect moderator-analysis of others (a type of control conditions)

Author(s) and Year	Experimental Total	Experimental Mean	Experimental SD	Control Total	Control Mean	Control SD	Hedges'g [95% CI]
Sakai et al. (2002)	6	−646.88	210.81	6	−651.44	186.39	0.02 [−1.11, 1.15]
Schuhmann et al. (2009)	10	475	53.82	8	454	38.05	0.40 [−0.54, 1.34]
Andoh et al. (2011), Exp1	10	−934.5	520.38	10	−957	525.59	0.04 [−0.84, 0.92]
Andoh et al. (2011), Exp2	10	990.5	396.55	10	967	315.04	0.06 [−0.82, 0.94]
Schuhmann et al. (2012), Exp1	8	471	51.89	8	455	44	0.30 [−0.69, 1.28]
Schuhmann et al. (2012), Exp2	8	488.2	40.01	8	474	39	0.32 [−0.67, 1.31]
Schuhmann et al. (2012), Exp3	8	477	39.61	8	470	29	0.18 [−0.80, 1.16]
Hartwigsena et al. (2013), Exp1	17	916.5	46.61	17	899.25	46.38	0.35 [−0.32, 1.03]
Hartwigsena et al. (2013), Exp2	17	916.5	46.61	17	912.25	44.85	0.09 [−0.58, 0.76]
Jackson et al. (2015)	15	1189.43	282.59	15	1134.04	302.41	0.18 [−0.54, 0.90]
Papeo et al. (2015), Exp1	14	−1033.7	261.45	14	−1045.67	163.54	0.05 [−0.69, 0.79]
Papeo et al. (2015), Exp2	14	1033.7	261.45	14	1008.9	293.48	0.08 [−0.66, 0.83]
Passeri et al. (2015), Exp1	18	578	23	18	567	22.02	0.47 [−0.20, 1.13]
Passeri et al. (2015), Exp2	18	574	26.62	18	567	22.02	0.27 [−0.38, 0.93]
Davey et al. (2015), Exp1	15	1293.67	451.24	15	1291.3	439.47	0.01 [−0.71, 0.72]
Davey et al. (2015), Exp2	15	−1259.2	439.12	15	−1291.3	439.47	0.07 [−0.65, 0.79]
Hartwigsen et al. (2016), Exp1	17	−973.16	700.55	17	−1021.29	727.83	0.06 [−0.61, 0.74]
Hartwigsen et al. (2016), Exp2	17	−1002.23	498.42	17	−1025.43	625.5	0.04 [−0.63, 0.71]
Klaus et al. (2018), Exp1	24	1789.5	432	24	1773	432	0.04 [−0.53, 0.60]
Klaus et al. (2018), Exp2	24	−1769.5	492.15	24	−1807	528	0.07 [−0.49, 0.64]
Zhang et al. (2018)	21	584.69	183.18	21	557.45	220.53	0.13 [−0.48, 0.73]
Klaus et al. (2019)	16	−608	191.44	16	−614.5	198.27	0.03 [−0.66, 0.72]
Ishkhanyan et al. (2020), Exp1	19	−559.33	116.35	19	−560.17	116.87	0.01 [−0.63, 0.64]
Ishkhanyan et al. (2020), Exp2	19	559	118.65	19	530.4	128.86	0.22 [−0.42, 0.86]
Deschamps et al. (2020)	18	11.36	491.91	18	9.05	412.25	0.00 [−0.65, 0.66]
Jost et al. (2020)	22	1219	241	22	1178	239	0.16 [−0.43, 0.76]
Piai et al. (2020)	24	−834.67	113.44	24	−840.17	112.87	0.05 [−0.52, 0.61]
Sliwinska et al. (2021)	20	1286.5	188.3	20	1279	176.16	0.04 [−0.58, 0.66]
Ward et al. (2022)	20	651.67	119.58	20	642	109.74	0.08 [−0.54, 0.70]
van der Burght et al. (2022), Exp1	30	−698.92	265.17	30	−715.04	242.57	0.06 [−0.44, 0.57]
van der Burght et al. (2022), Exp2	30	894.78	327.97	30	878.17	291.38	0.05 [−0.45, 0.56]
Random Effect Model Total (Z = 1.846, P = 0.065)							0.11 [−0.01, 0.24]

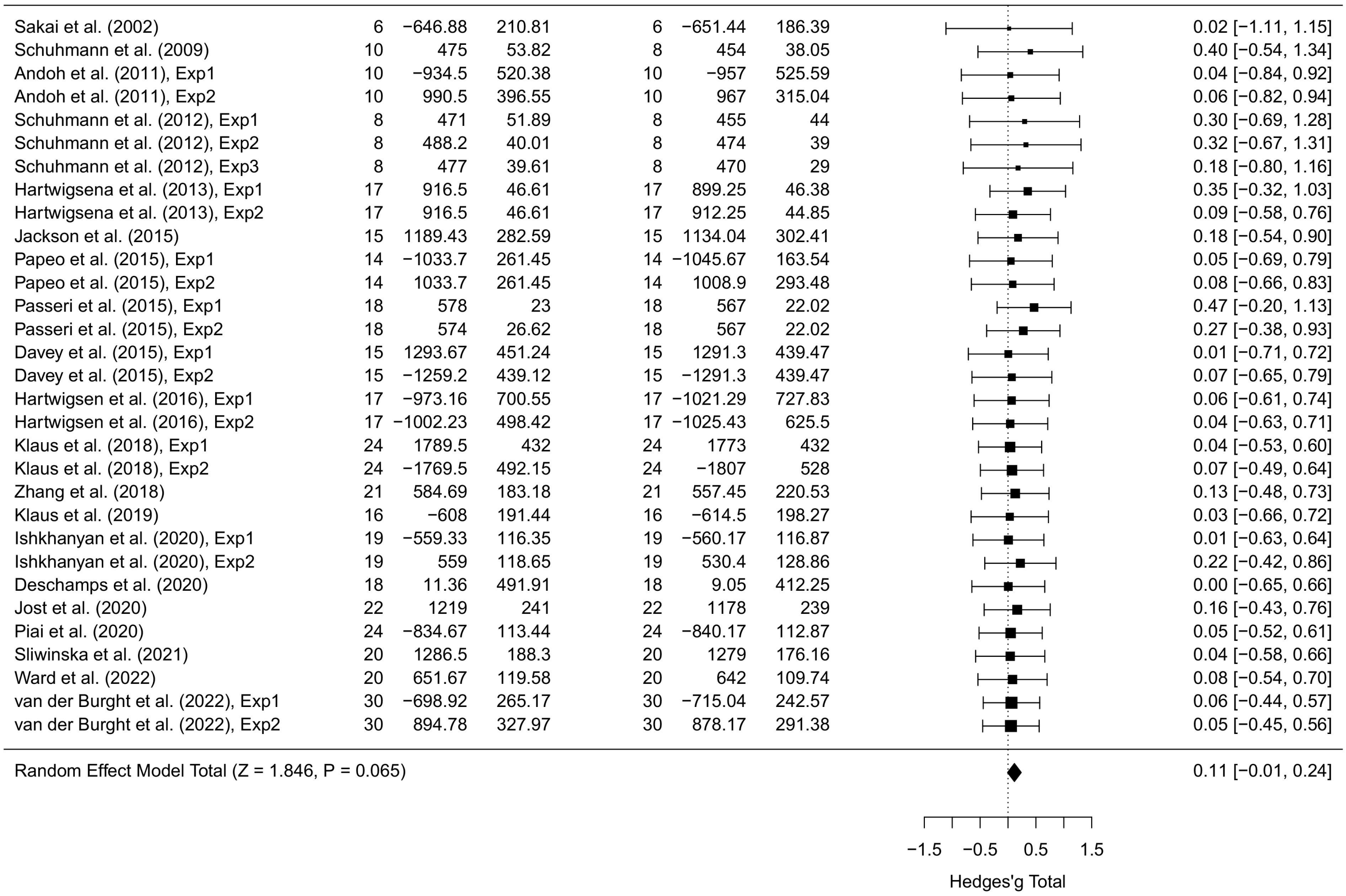

Supplementary Figure S29. Forest plot of RT effect moderator-analysis of within-subject group design

Author(s) and Year	Experimental Total	Experimental Mean	Experimental SD	Control Total	Control Mean	Control SD	Hedges'g [95% CI]
Finocchiaro et al. (2008)	6	−51	55.15	5	−87.5	132.38	0.31 [−0.88, 1.51]
Hartwigsen et al. (2010), Exp1	28	1070.67	1371.93	7	755.83	328.64	0.24 [−0.59, 1.07]
Hartwigsen et al. (2010), Exp2	28	1022.5	1024.46	7	686	113.5	0.35 [−0.49, 1.18]
Willems et al. (2011), Exp1	18	712.13	174.48	8	654.87	177.12	0.31 [−0.53, 1.14]
Willems et al. (2011), Exp2	18	729.87	193.86	8	670.67	197.77	0.28 [−0.55, 1.12]
Finocchiaro et al. (2015), Exp1	12	−1120.33	375.36	18	−1381.27	274.8	0.78 [0.02, 1.53]
Finocchiaro et al. (2015), Exp2	12	−1151.13	305.64	18	−1381.27	274.8	0.76 [0.00, 1.51]
Finocchiaro et al. (2015), Exp3	12	−1133.38	242.97	18	−1381.27	274.8	0.89 [0.13, 1.66]
Karabanov et al. (2015), Exp1	26	949	8060	26	861	9048	0.01 [−0.53, 0.55]
Karabanov et al. (2015), Exp2	26	−781	8970	26	−829	7384	0.01 [−0.54, 0.55]
Woollamsa et al. (2017)	18	−548	72.28	18	−550.38	66.93	0.03 [−0.62, 0.69]
Finocchiaro et al. (2021)	16	−1742.12	1192.34	16	−1748	1142.47	0.00 [−0.69, 0.70]
Random Effect Model Total (Z = 1.877, P = 0.06)							0.27 [−0.01, 0.55]

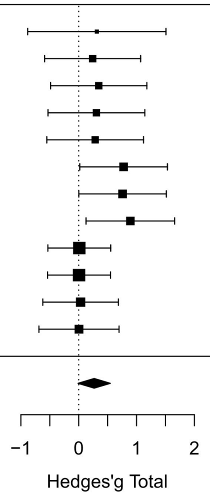

Supplementary Figure S30. Forest plot of RT effect moderator-analysis of between-subject group design

Author(s) and Year	Experimental Total	Experimental Mean	Experimental SD	Control Total	Control Mean	Control SD	Hedges'g [95% CI]
Hartwigsen et al. (2010), Exp1	28	−90.5	1	7	−94	11.1	0.69 [−0.15, 1.54]
Hartwigsen et al. (2010), Exp2	28	96.3	34.3	7	94.3	15.4	0.06 [−0.77, 0.89]
Andoh et al. (2011)	10	80	27.8	10	78.1	36.5	0.05 [−0.82, 0.93]
Franzmeier et al. (2012), Exp1	59	90.2	11.9	59	89.2	13	0.08 [−0.28, 0.44]
Franzmeier et al. (2012), Exp2	59	−89.1	12.3	59	−89.2	13	0.01 [−0.35, 0.37]
Whitney et al. (2012), Exp1	16	−91.1	6.4	16	−91.6	6.6	0.07 [−0.62, 0.77]
Whitney et al. (2012), Exp2	16	−90.8	6.2	16	−91.5	6	0.11 [−0.58, 0.80]
Whitney et al. (2012), Exp3	16	89.9	9.4	16	89.1	8.4	0.09 [−0.61, 0.78]
Hartwigsena et al. (2013), Exp1	17	−98.5	0.6	17	−98.6	0.1	0.22 [−0.45, 0.90]
Hartwigsena et al. (2013), Exp2	17	98.5	0.6	17	98.5	0.8	0.00 [−0.67, 0.67]
Krieger−Redwood et al. (2013), Exp1	15	−88.7	28.1	15	−91.3	29.5	0.09 [−0.63, 0.80]
Krieger−Redwood et al. (2013), Exp2	15	−86.9	36.3	15	−89.7	28.4	0.08 [−0.63, 0.80]
Krieger−Redwood et al. (2013), Exp3	15	−90.1	28.6	15	−91.2	26.1	0.04 [−0.68, 0.75]
Krieger−Redwood et al. (2014), Exp1	18	−93.9	8.9	18	−94.4	6.6	0.06 [−0.59, 0.71]
Krieger−Redwood et al. (2014), Exp2	18	−94.7	5.5	18	−95.5	4.3	0.15 [−0.50, 0.81]
Jackson et al. (2015)	15	−89.4	6.4	15	−91.8	5.8	0.37 [−0.35, 1.09]
Papeo et al. (2015), Exp1	14	−92.2	12.1	14	−93.8	13.1	0.12 [−0.62, 0.86]
Papeo et al. (2015), Exp2	14	−92.2	12.1	14	−94.8	10.8	0.21 [−0.53, 0.96]
Bonnì et al. (2015)	18	87.4	6.9	18	86.5	5.6	0.14 [−0.52, 0.79]
Davey et al. (2015), Exp1	15	94	4.7	15	93.5	22.5	0.03 [−0.69, 0.74]
Davey et al. (2015), Exp2	15	93.5	5.7	15	93.5	22.5	0.00 [−0.72, 0.72]
Hartwigsen et al. (2016), Exp1	17	97.2	14.5	17	94.8	26.8	0.11 [−0.57, 0.78]
Hartwigsen et al. (2016), Exp2	17	−96	14.9	17	−96.2	12.8	0.01 [−0.66, 0.69]
Woollamsa et al. (2017)	18	96.2	3.9	18	95.9	3.9	0.07 [−0.58, 0.73]
Klaus et al. (2018), Exp1	24	89.3	21.6	24	88.4	21.6	0.04 [−0.53, 0.61]
Klaus et al. (2018), Exp2	24	−80.7	26.4	24	−80.8	26.4	0.00 [−0.56, 0.57]
Zhang et al. (2018)	21	−88.4	47	21	−91.2	38.7	0.06 [−0.54, 0.67]
Klaus et al. (2019)	16	−97	1.8	16	−98	1.5	0.57 [−0.13, 1.28]
Ohlerth et al. (2021)	18	−85.5	7.8	18	−92.7	3.5	1.14 [0.43, 1.85]
Finocchiaro et al. (2021)	16	89.4	56.9	16	87.3	58	0.03 [−0.66, 0.73]
Vitale et al. (2022)	32	97	2	32	96	3	0.38 [−0.11, 0.88]
van der Burght et al. (2022), Exp2	30	−83.7	36.9	30	−87.6	33	0.11 [−0.40, 0.61]
Random Effect Model Total (Z = 2.522, P = 0.012)							0.14 [0.03, 0.25]

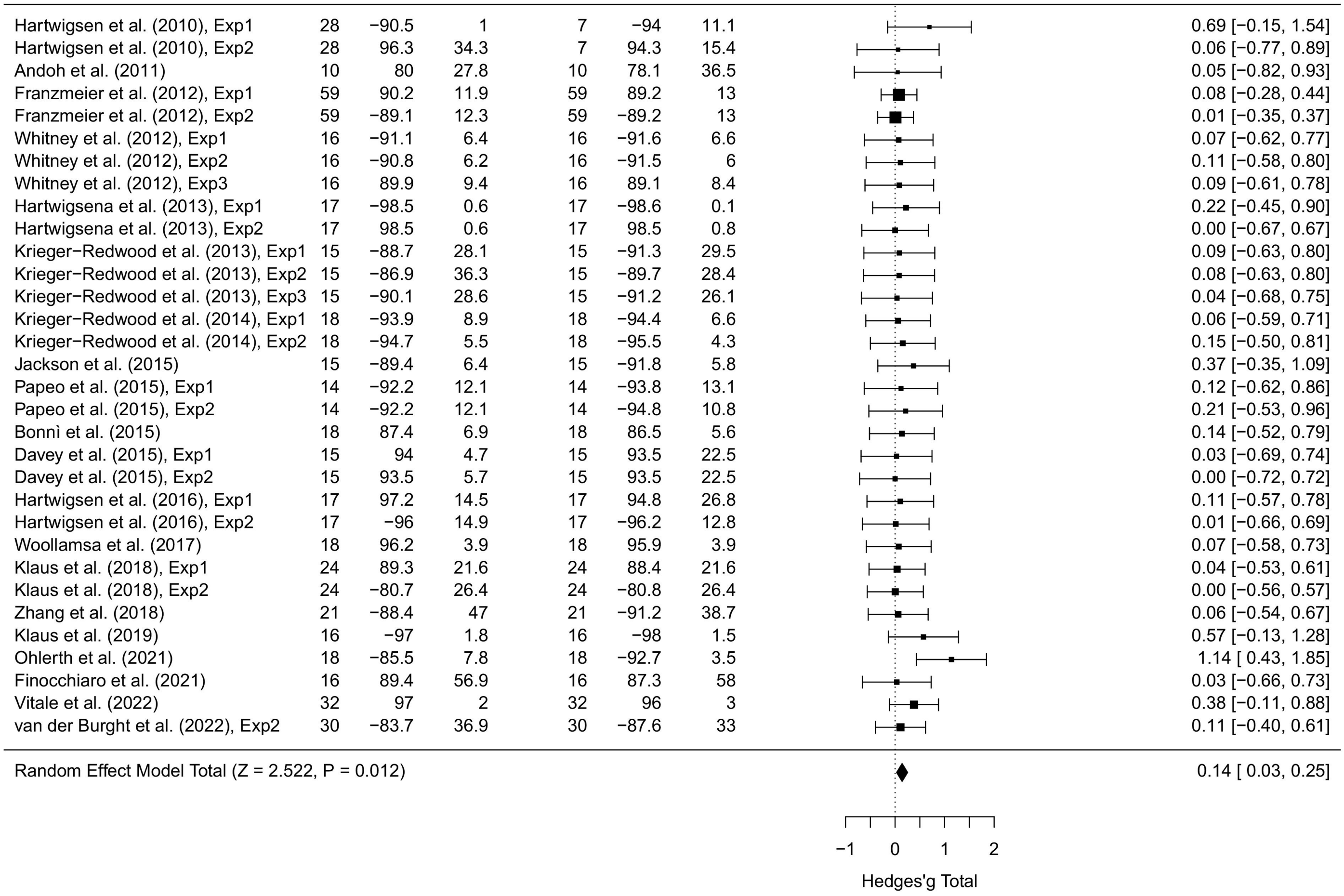

Supplementary Figure S31. Forest plot of ACC effect moderator-analysis of semantic tasks

Author(s) and Year	Experimental Total	Experimental Mean	Experimental SD	Control Total	Control Mean	Control SD	Hedges'g [95% CI]
Uddén et al. (2017)	13	−53	4.5	13	−55.5	3.2	0.60 [−0.19, 1.39]
Finocchiaro et al. (2021)	16	89.4	56.9	16	87.3	58	0.03 [−0.66, 0.73]
van der Burght et al. (2022), Exp1	30	−85.5	35.2	30	−88.3	32.1	0.08 [−0.43, 0.59]
Random Effect Model Total (Z = 0.965, P = 0.334)							0.18 [−0.18, 0.54]

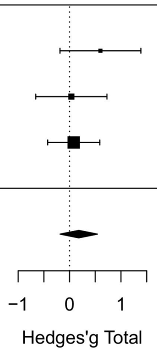

Supplementary Figure S32. Forest plot of ACC effect moderator-analysis of syntax tasks

Author(s) and Year	Experimental Total	Experimental Mean	Experimental SD	Control Total	Control Mean	Control SD	Hedges'g [95% CI]
Hartwigsen et al. (2010), Exp1	28	−90.5	1	7	−94	11.1	0.69 [−0.15, 1.54]
Hartwigsen et al. (2010), Exp2	28	96.3	34.3	7	94.3	15.4	0.06 [−0.77, 0.89]
Andoh et al. (2011)	10	80	27.8	10	78.1	36.5	0.05 [−0.82, 0.93]
Hartwigsena et al. (2013), Exp1	17	−98.5	0.6	17	−98.6	0.1	0.22 [−0.45, 0.90]
Hartwigsena et al. (2013), Exp2	17	98.5	0.6	17	98.5	0.8	0.00 [−0.67, 0.67]
Krieger−Redwood et al. (2013)	15	−88.6	31.2	15	−90.7	28	0.07 [−0.65, 0.78]
Deschamps et al. (2013)	16	98.1	2.9	16	98.1	3.2	0.00 [−0.69, 0.69]
Karabanov et al. (2015), Exp1	26	−94	62.4	26	−96.4	36.4	0.05 [−0.50, 0.59]
Karabanov et al. (2015), Exp2	26	−94.6	59.8	26	−95.9	59.8	0.02 [−0.52, 0.56]
Hartwigsen et al. (2016), Exp1	17	97.2	14.5	17	94.8	26.8	0.11 [−0.57, 0.78]
Hartwigsen et al. (2016), Exp2	17	−96	14.9	17	−96.2	12.8	0.01 [−0.66, 0.69]
Klaus et al. (2018), Exp1	24	89.3	21.6	24	88.4	21.6	0.04 [−0.53, 0.61]
Klaus et al. (2018), Exp2	24	−80.7	26.4	24	−80.8	26.4	0.00 [−0.56, 0.57]
Zhang et al. (2018)	21	−88.4	47	21	−91.2	38.7	0.06 [−0.54, 0.67]
Klaus et al. (2019)	16	−97	1.8	16	−98	1.5	0.57 [−0.13, 1.28]
Ohlerth et al. (2021)	18	−85.5	7.8	18	−92.7	3.5	1.14 [0.43, 1.85]
Smalle et al. (2022)	18	68.8	63	18	57.3	61.2	0.18 [−0.48, 0.83]
Random Effect Model Total (Z = 2.001, P = 0.045)							0.16 [0.00, 0.32]

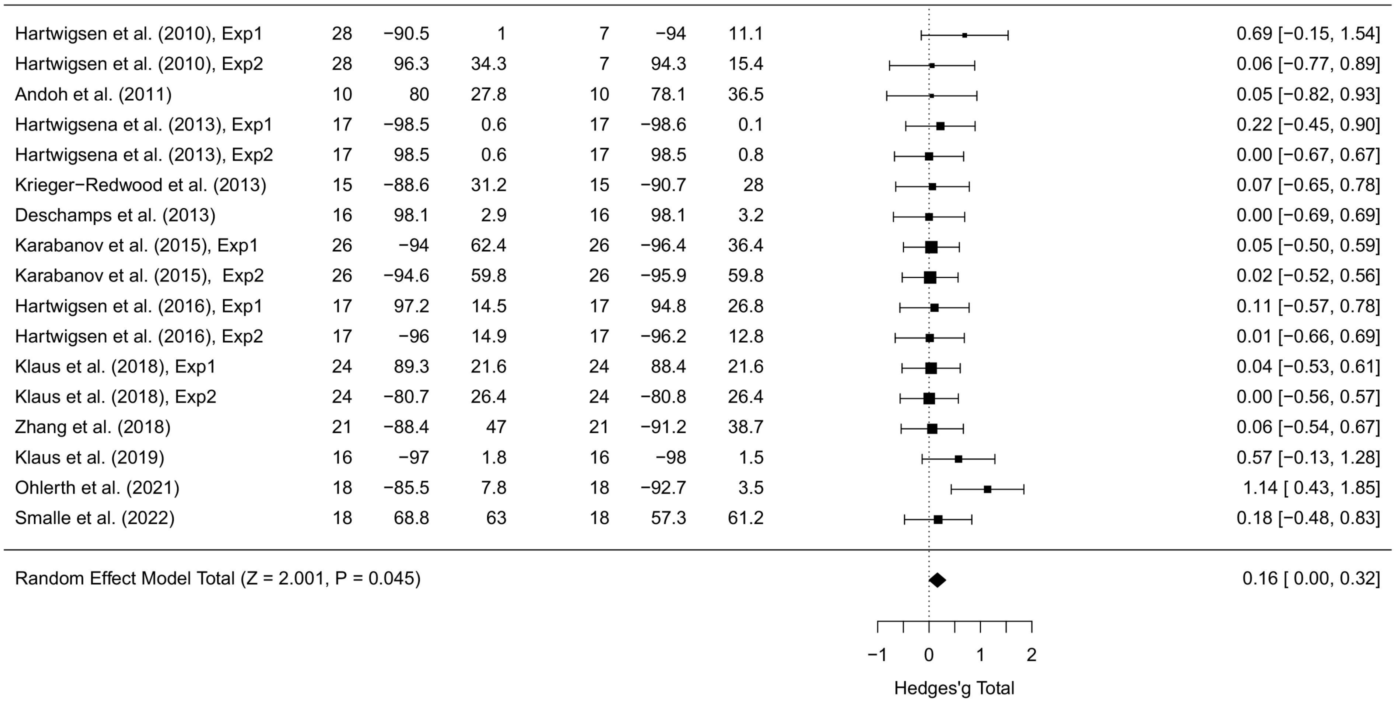

Supplementary Figure S33. Forest plot of ACC effect moderator-analysis of phonological tasks

Author(s) and Year	Experimental Total	Experimental Mean	Experimental SD	Control Total	Control Mean	Control SD	Hedges'g [95% CI]
Hartwigsen et al. (2010), Exp1	28	−90.5	1	7	−94	11.1	0.69 [−0.15, 1.54]
Hartwigsen et al. (2010), Exp2	28	96.3	34.3	7	94.3	15.4	0.06 [−0.77, 0.89]
Whitney et al. (2012), Exp1	16	−91.1	6.4	16	−91.6	6.6	0.07 [−0.62, 0.77]
Hartwigsena et al. (2013), Exp1	17	−98.5	0.6	17	−98.6	0.1	0.22 [−0.45, 0.90]
Hartwigsena et al. (2013), Exp2	17	98.5	0.6	17	98.5	0.8	0.00 [−0.67, 0.67]
Karabanov et al. (2015), Exp2	26	−94.6	59.8	26	−95.9	59.8	0.02 [−0.52, 0.56]
Hartwigsen et al. (2016), Exp1	17	97.1	15.6	17	95	22.8	0.10 [−0.57, 0.78]
Uddén et al. (2017)	13	−53	4.5	13	−55.5	3.2	0.60 [−0.19, 1.39]
Klaus et al. (2018), Exp1	24	85	24.1	24	84.6	24.1	0.02 [−0.55, 0.58]
Klaus et al. (2018), Exp2	24	85	24.1	24	84.6	24.1	0.02 [−0.55, 0.58]
Zhang et al. (2018)	21	−88.4	47	21	−91.2	38.7	0.06 [−0.54, 0.67]
Smalle et al. (2022)	18	68.8	63	18	57.3	61.2	0.18 [−0.48, 0.83]
van der Burght et al. (2022), Exp1	30	−85.5	35.2	30	−88.3	32.1	0.08 [−0.43, 0.59]
van der Burght et al. (2022), Exp2	30	−83.7	36.9	30	−87.6	33	0.11 [−0.40, 0.61]
Random Effect Model Total (Z = 1.44, P = 0.15)							0.12 [−0.04, 0.29]

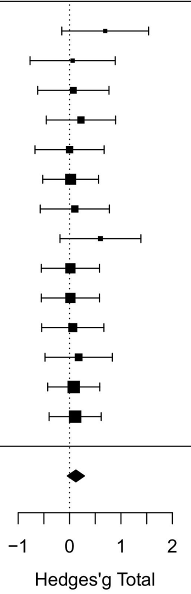

Supplementary Figure S34. Forest plot of ACC effect moderator-analysis of frontal

Author(s) and Year	Experimental Total	Experimental Mean	Experimental SD	Control Total	Control Mean	Control SD	Hedges'g [95% CI]
Andoh et al. (2011)	10	82.5	29.1	10	77.2	32.1	0.16 [−0.72, 1.04]
Franzmeier et al. (2012), Exp1	59	90.2	11.9	59	89.2	13	0.08 [−0.28, 0.44]
Whitney et al. (2012),Exp2	16	−90.8	6.2	16	−91.5	6	0.11 [−0.58, 0.80]
Karabanov et al. (2015), Exp1	26	−94	62.4	26	−96.4	36.4	0.05 [−0.50, 0.59]
Davey et al. (2015), Exp1	15	94	4.7	15	93.5	22.5	0.03 [−0.69, 0.74]
Woollamsa et al. (2017)	18	96.2	3.9	18	95.9	3.9	0.07 [−0.58, 0.73]
Random Effect Model Total (Z = 0.645, P = 0.519)							0.08 [−0.16, 0.31]

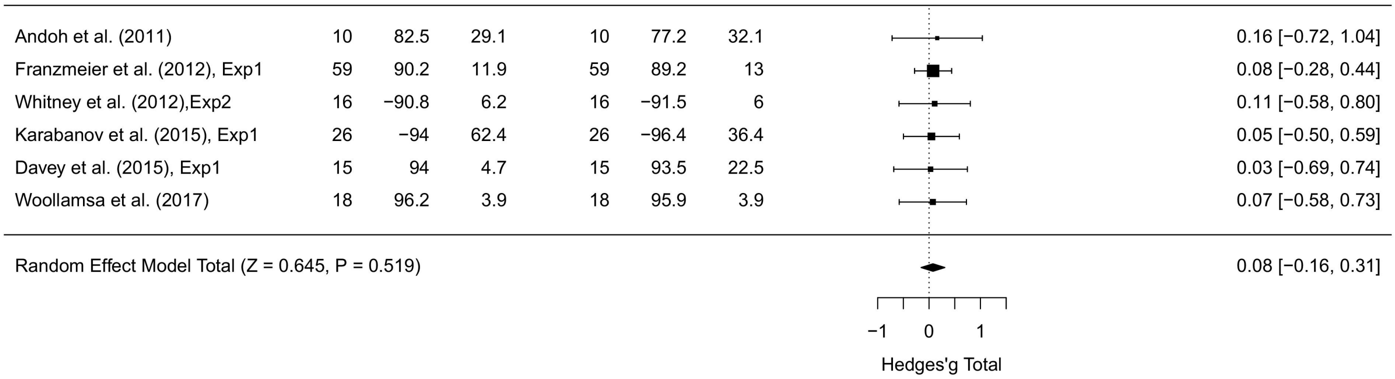

Supplementary Figure S35. Forest plot of ACC effect moderator-analysis of temporal

Author(s) and Year	Experimental Total	Experimental Mean	Experimental SD	Control Total	Control Mean	Control SD	Hedges'g [95% CI]
Whitney et al. (2012),Exp3	16	89.9	9.4	16	89.1	8.4	0.09 [−0.61, 0.78]
Deschamps et al. (2013)	16	−97.5	3.4	16	−98	3.2	0.14 [−0.55, 0.84]
Hartwigsen et al. (2016), Exp1	17	97.3	13.3	17	94.7	30.3	0.11 [−0.57, 0.78]
Finocchiaro et al. (2021)	16	89.4	56.9	16	87.3	58	0.03 [−0.66, 0.73]
Random Effect Model Total (Z = 0.528, P = 0.598)							0.09 [−0.25, 0.44]

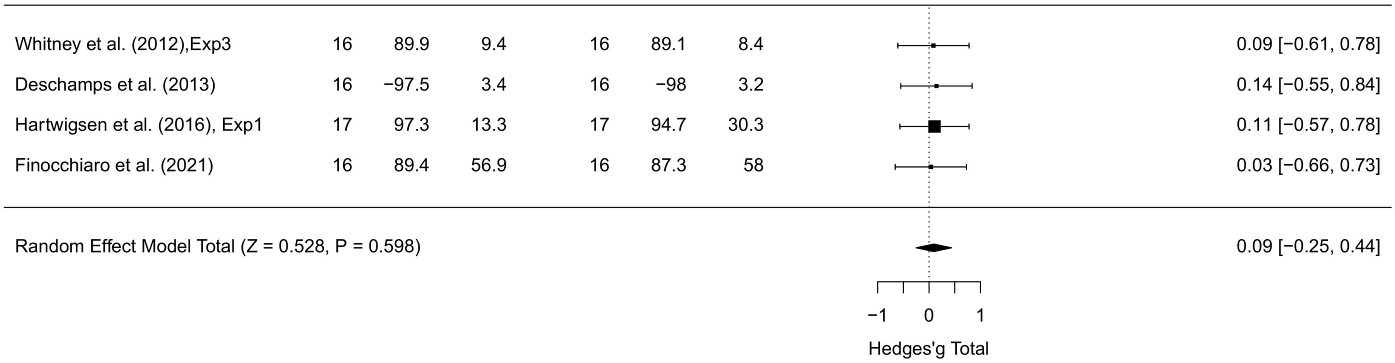

Supplementary Figure S36. Forest plot of ACC effect moderator-analysis of parietal

Author(s) and Year	Experimental Total	Experimental Mean	Experimental SD	Control Total	Control Mean	Control SD	Hedges'g [95% CI]
Hartwigsen et al. (2010), Exp1	28	−90.5	1	7	−94	11.1	0.69 [−0.15, 1.54]
Hartwigsen et al. (2010), Exp2	28	96.3	34.3	7	94.3	15.4	0.06 [−0.77, 0.89]
Whitney et al. (2012), Exp1	16	−91.1	6.4	16	−91.6	6.6	0.07 [−0.62, 0.77]
Hartwigsena et al. (2013), Exp1	17	−98.5	0.6	17	−98.6	0.1	0.22 [−0.45, 0.90]
Hartwigsena et al. (2013), Exp2	17	98.5	0.6	17	98.5	0.8	0.00 [−0.67, 0.67]
Krieger−Redwood et al. (2014), Exp1	18	−93.9	8.9	18	−94.4	6.6	0.06 [−0.59, 0.71]
Karabanov et al. (2015), Exp2	26	−94.6	59.8	26	−95.9	59.8	0.02 [−0.52, 0.56]
Hartwigsen et al. (2016), Exp1	17	97.1	15.6	17	95	22.8	0.10 [−0.57, 0.78]
Uddén et al. (2017)	13	−53	4.5	13	−55.5	3.2	0.60 [−0.19, 1.39]
Klaus et al. (2018), Exp1	24	89.3	21.6	24	88.4	21.6	0.04 [−0.53, 0.61]
Klaus et al. (2018), Exp2	24	−80.7	26.4	24	−80.8	26.4	0.00 [−0.56, 0.57]
Zhang et al. (2018)	21	−88.4	47	21	−91.2	38.7	0.06 [−0.54, 0.67]
Vitale et al. (2022)	32	97	2	32	96	3	0.38 [−0.11, 0.88]
van der Burght et al. (2022), Exp1	30	−85.5	35.2	30	−88.3	32.1	0.08 [−0.43, 0.59]
van der Burght et al. (2022), Exp2	30	−83.7	36.9	30	−87.6	33	0.11 [−0.40, 0.61]
Random Effect Model Total (Z = 1.778, P = 0.075)							0.14 [−0.01, 0.30]

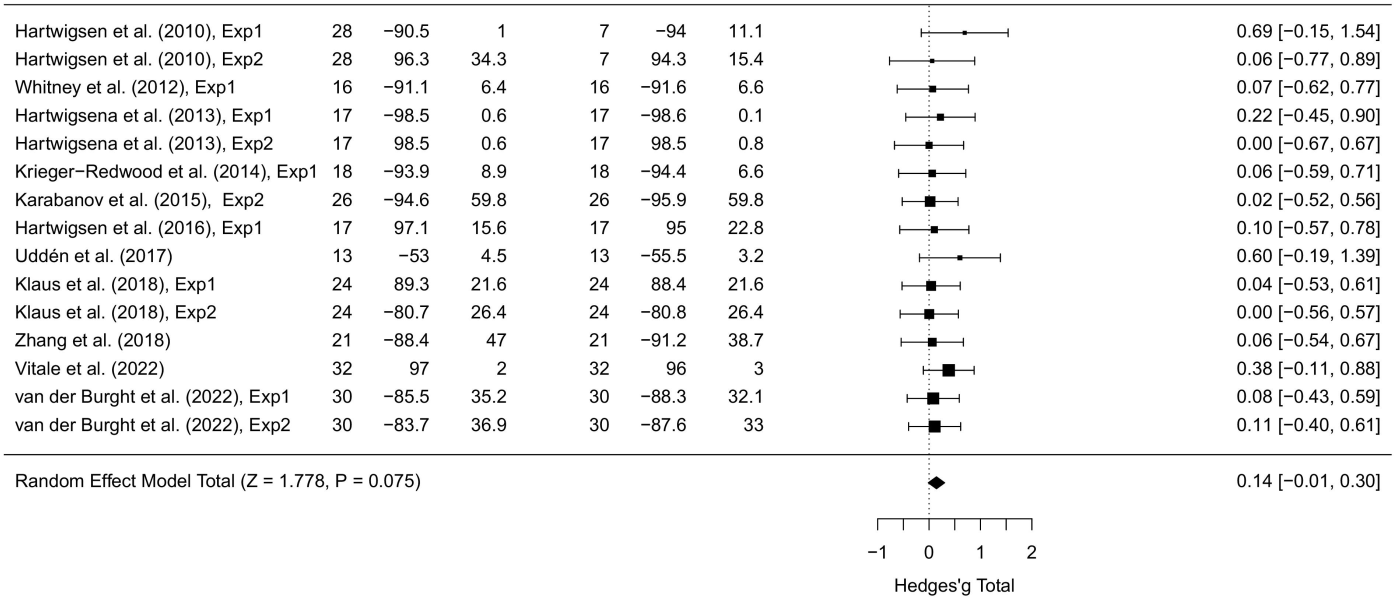

Supplementary Figure S37. Forest plot of ACC effect moderator-analysis of IFG

Author(s) and Year	Experimental Total	Experimental Mean	Experimental SD	Control Total	Control Mean	Control SD	Hedges'g [95% CI]
Krieger−Redwood et al. (2013)	15	86.9	36.33	15	89.7	28.4	−0.08 [−0.80, 0.63]
Karabanov et al. (2015), Exp1	26	94	62.4	26	96.4	36.4	−0.05 [−0.59, 0.50]
Random Effect Model Total (Z = −0.265, P = 0.791)							−0.06 [−0.49, 0.37]

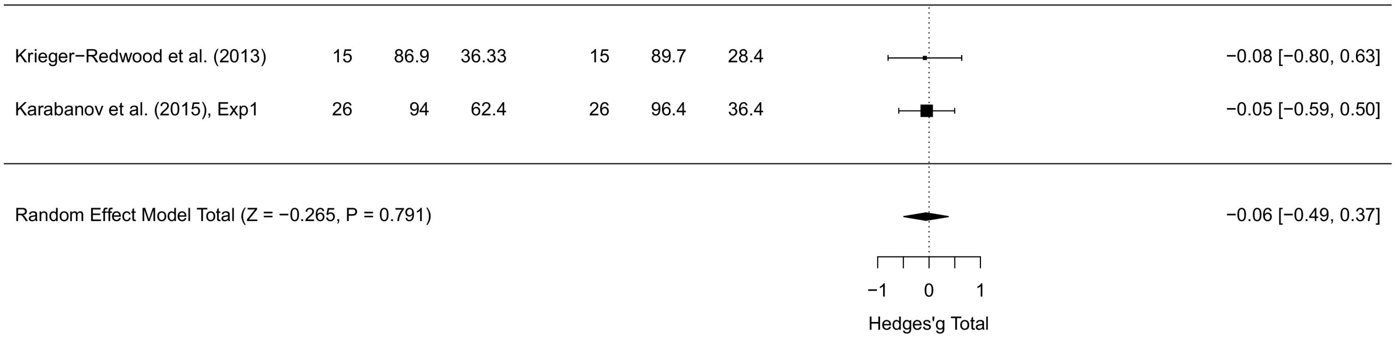

Supplementary Figure S38. Forest plot of ACC effect moderator-analysis of STG

Author(s) and Year	Experimental Total	Experimental Mean	Experimental SD	Control Total	Control Mean	Control SD	Hedges'g [95% CI]
Whitney et al. (2012), Exp2	16	−90.8	6.2	16	−91.5	6	0.11 [−0.58, 0.80]
Papeo et al. (2015), Exp1	14	−92.2	12.1	14	−93.8	13.1	0.12 [−0.62, 0.86]
Papeo et al. (2015), Exp2	14	−92.2	12.1	14	−94.8	10.8	0.21 [−0.53, 0.96]
Klaus et al. (2019)	16	−97	1.8	16	−98	1.5	0.57 [−0.13, 1.28]
Random Effect Model Total (Z = 1.395, P = 0.163)							0.26 [−0.10, 0.62]

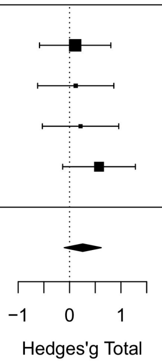

Supplementary Figure S39. Forest plot of ACC effect moderator-analysis of MTG

Author(s) and Year	Experimental Total	Experimental Mean	Experimental SD	Control Total	Control Mean	Control SD	Hedges'g [95% CI]
Hartwigsen et al. (2010), Exp1	28	−90.5	1	7	−94	11.1	0.69 [−0.15, 1.54]
Hartwigsen et al. (2010), Exp2	28	96.3	34.3	7	94.3	15.4	0.06 [−0.77, 0.89]
Andoh et al. (2011)	10	80	27.8	10	78.1	36.5	0.05 [−0.82, 0.93]
Whitney et al. (2012), Exp1	16	−91.1	6.4	16	−91.6	6.6	0.07 [−0.62, 0.77]
Whitney et al. (2012), Exp2	16	−90.8	6.2	16	−91.5	6	0.11 [−0.58, 0.80]
Whitney et al. (2012), Exp3	16	89.9	9.4	16	89.1	8.4	0.09 [−0.61, 0.78]
Hartwigsena et al. (2013), Exp1	17	−98.5	0.6	17	−98.6	0.1	0.22 [−0.45, 0.90]
Hartwigsena et al. (2013), Exp2	17	98.5	0.6	17	98.5	0.8	0.00 [−0.67, 0.67]
Krieger−Redwood et al. (2013)	15	−88.6	31.2	15	−90.7	28	0.07 [−0.65, 0.78]
Deschamps et al. (2013)	16	98.1	2.9	16	98.1	3.2	0.00 [−0.69, 0.69]
Krieger−Redwood et al. (2014), Exp1	18	−93.9	8.9	18	−94.4	6.6	0.06 [−0.59, 0.71]
Krieger−Redwood et al. (2014), Exp2	18	−94.8	5.4	18	−95.7	4.2	0.18 [−0.48, 0.83]
Papeo et al. (2015), Exp1	14	−92.2	12.1	14	−93.8	13.1	0.12 [−0.62, 0.86]
Papeo et al. (2015), Exp2	14	−92.2	12.1	14	−94.8	10.8	0.21 [−0.53, 0.96]
Bonnì et al. (2015)	18	87.4	6.9	18	86.5	5.6	0.14 [−0.52, 0.79]
Davey et al. (2015), Exp1	15	94	4.7	15	93.5	22.5	0.03 [−0.69, 0.74]
Davey et al. (2015), Exp2	15	93.5	5.7	15	93.5	22.5	0.00 [−0.72, 0.72]
Hartwigsen et al. (2016), Exp1	17	97.2	14.5	17	94.8	26.8	0.11 [−0.57, 0.78]
Hartwigsen et al. (2016), Exp2	17	−96	14.9	17	−96.2	12.8	0.01 [−0.66, 0.69]
Uddén et al. (2017)	13	−53	4.5	13	−55.5	3.2	0.60 [−0.19, 1.39]
Woollamsa et al. (2017)	18	96.2	3.9	18	95.9	3.9	0.07 [−0.58, 0.73]
Klaus et al. (2018), Exp1	24	85	24.1	24	84.6	24.1	0.02 [−0.55, 0.58]
Klaus et al. (2018), Exp2	24	85	24.1	24	84.6	24.1	0.02 [−0.55, 0.58]
Zhang et al. (2018)	21	−88.4	47	21	−91.2	38.7	0.06 [−0.54, 0.67]
Klaus et al. (2019)	16	−97	1.8	16	−98	1.5	0.57 [−0.13, 1.28]
Finocchiaro et al. (2021)	16	89.4	56.9	16	87.3	58	0.03 [−0.66, 0.73]
Vitale et al. (2022)	32	97	2	32	96	3	0.38 [−0.11, 0.88]
van der Burght et al. (2022), Exp1	30	−85.5	35.2	30	−88.3	32.1	0.08 [−0.43, 0.59]
van der Burght et al. (2022), Exp2	30	−83.7	36.9	30	−87.6	33	0.11 [−0.40, 0.61]
Random Effect Model Total (Z = 2.17, P = 0.03)							0.14 [0.01, 0.26]

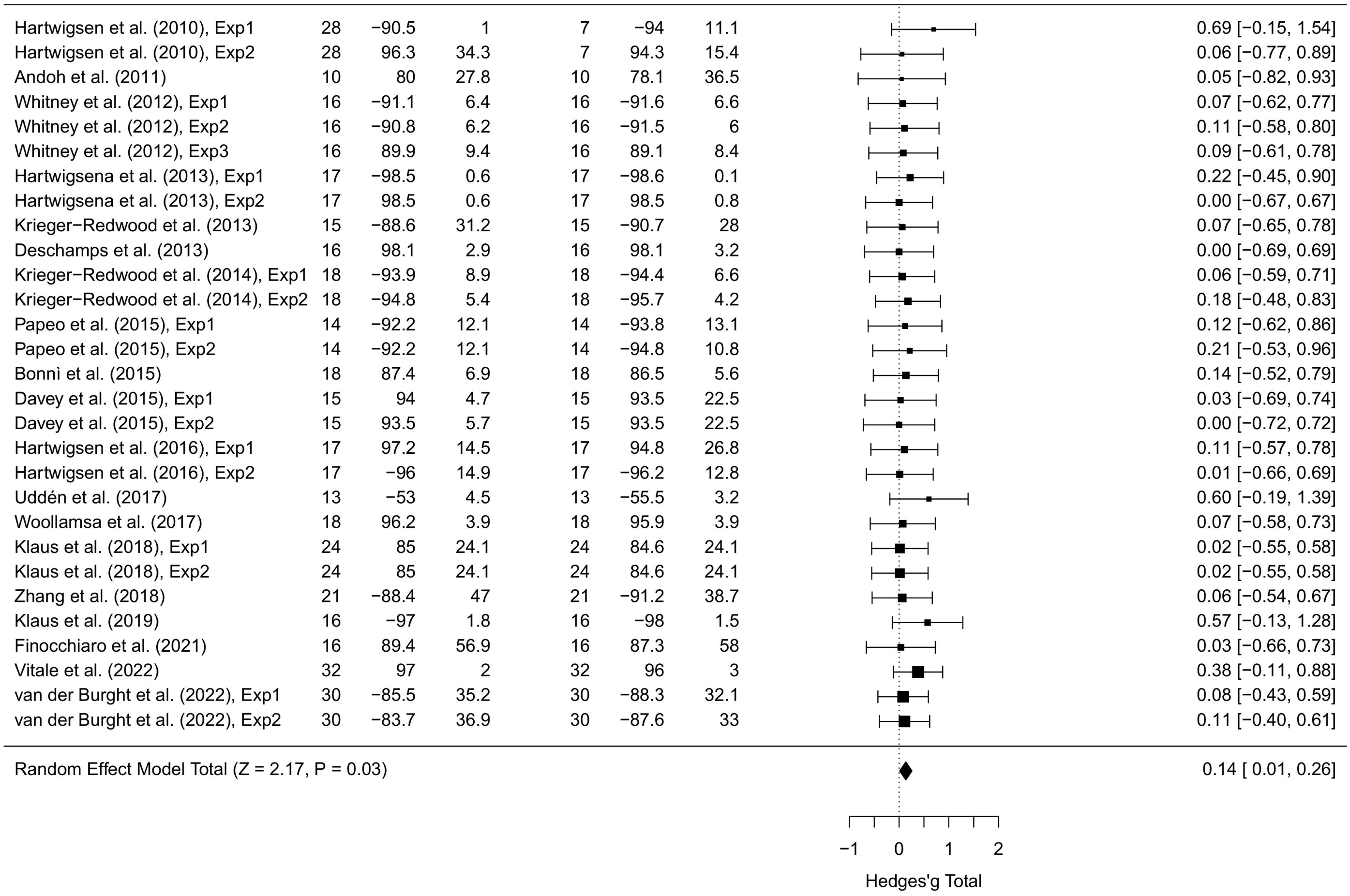

Supplementary Figure S40. Forest plot of ACC effect moderator-analysis of localization derived from previous study

Author(s) and Year	Experimental Total	Experimental Mean	Experimental SD	Control Total	Control Mean	Control SD	Hedges'g [95% CI]
Franzmeier et al. (2012), Exp1	59	90.2	11.9	59	89.2	13	0.08 [−0.28, 0.44]
Franzmeier et al. (2012), Exp2	59	−89.1	12.3	59	−89.2	13	0.01 [−0.35, 0.37]
Jackson et al. (2015)	15	−89.4	6.4	15	−91.8	5.8	0.37 [−0.35, 1.09]
Karabanov et al. (2015), Exp1	26	−94	62.4	26	−96.4	36.4	0.05 [−0.50, 0.59]
Karabanov et al. (2015), Exp2	26	−94.6	59.8	26	−95.9	59.8	0.02 [−0.52, 0.56]
Ohlerth et al. (2021)	18	−85.5	7.8	18	−92.7	3.5	1.14 [0.43, 1.85]
Smalle et al. (2022)	18	68.8	63	18	57.3	61.2	0.18 [−0.48, 0.83]
Random Effect Model Total (Z = 1.508, P = 0.131)							0.27 [−0.08, 0.62]

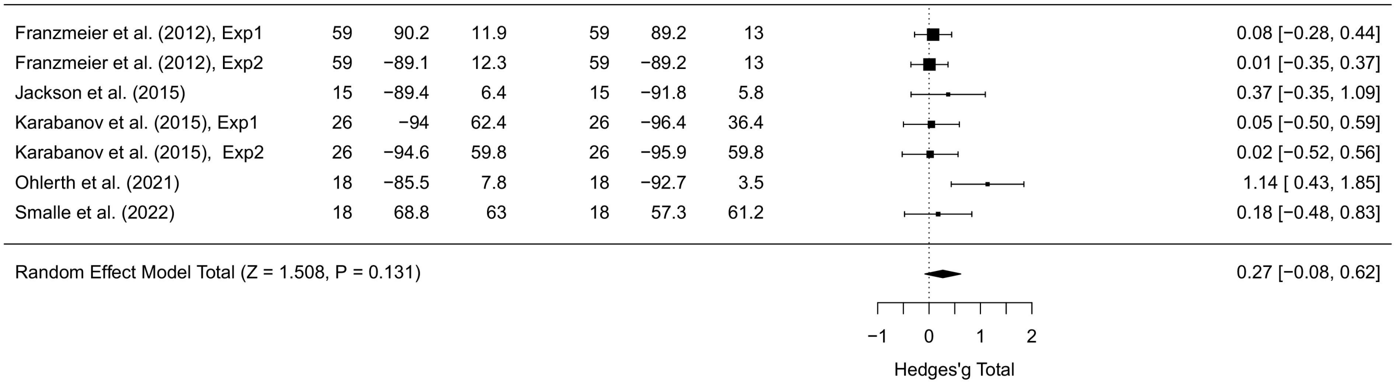

Supplementary Figure S41. Forest plot of ACC effect moderator-analysis of localization derived from current study

Author(s) and Year	Experimental Total	Experimental Mean	Experimental SD	Control Total	Control Mean	Control SD	Hedges'g [95% CI]
Hartwigsen et al. (2010), Exp1	28	−90.5	1	7	−94	11.1	0.69 [−0.15, 1.54]
Hartwigsen et al. (2010), Exp2	28	96.3	34.3	7	94.3	15.4	0.06 [−0.77, 0.89]
Andoh et al. (2011)	10	80	27.8	10	78.1	36.5	0.05 [−0.82, 0.93]
Whitney et al. (2012), Exp1	16	−91.1	6.4	16	−91.6	6.6	0.07 [−0.62, 0.77]
Whitney et al. (2012), Exp2	16	−90.8	6.2	16	−91.5	6	0.11 [−0.58, 0.80]
Whitney et al. (2012), Exp3	16	89.9	9.4	16	89.1	8.4	0.09 [−0.61, 0.78]
Krieger−Redwood et al. (2013)	15	−88.6	31.2	15	−90.7	28	0.07 [−0.65, 0.78]
Deschamps et al. (2013)	16	98.1	2.9	16	98.1	3.2	0.00 [−0.69, 0.69]
Krieger−Redwood et al. (2014), Exp1	18	−93.9	8.9	18	−94.4	6.6	0.06 [−0.59, 0.71]
Krieger−Redwood et al. (2014), Exp2	18	−94.8	5.4	18	−95.7	4.2	0.18 [−0.48, 0.83]
Jackson et al. (2015)	15	−89.4	6.4	15	−91.8	5.8	0.37 [−0.35, 1.09]
Papeo et al. (2015), Exp1	14	−92.2	12.1	14	−93.8	13.1	0.12 [−0.62, 0.86]
Papeo et al. (2015), Exp2	14	−92.2	12.1	14	−94.8	10.8	0.21 [−0.53, 0.96]
Karabanov et al. (2015), Exp1	26	−94	62.4	26	−96.4	36.4	0.05 [−0.50, 0.59]
Karabanov et al. (2015), Exp2	26	−94.6	59.8	26	−95.9	59.8	0.02 [−0.52, 0.56]
Davey et al. (2015), Exp1	15	94	4.7	15	93.5	22.5	0.03 [−0.69, 0.74]
Davey et al. (2015), Exp2	15	93.5	5.7	15	93.5	22.5	0.00 [−0.72, 0.72]
Hartwigsen et al. (2016), Exp1	17	97.2	14.5	17	94.8	26.8	0.11 [−0.57, 0.78]
Hartwigsen et al. (2016), Exp2	17	−96	14.9	17	−96.2	12.8	0.01 [−0.66, 0.69]
Uddén et al. (2017)	13	−53	4.5	13	−55.5	3.2	0.60 [−0.19, 1.39]
Woollamsa et al. (2017)	18	96.2	3.9	18	95.9	3.9	0.07 [−0.58, 0.73]
Klaus et al. (2018), Exp1	24	89.3	21.6	24	88.4	21.6	0.04 [−0.53, 0.61]
Klaus et al. (2018), Exp2	24	−80.7	26.4	24	−80.8	26.4	0.00 [−0.56, 0.57]
Zhang et al. (2018)	21	−88.4	47	21	−91.2	38.7	0.06 [−0.54, 0.67]
Ohlerth et al. (2021)	18	−85.5	7.8	18	−92.7	3.5	1.14 [0.43, 1.85]
Vitale et al. (2022)	32	97	2	32	96	3	0.38 [−0.11, 0.88]
van der Burght et al. (2022), Exp1	30	−85.5	35.2	30	−88.3	32.1	0.08 [−0.43, 0.59]
van der Burght et al. (2022), Exp2	30	−83.7	36.9	30	−87.6	33	0.11 [−0.40, 0.61]
Random Effect Model Total (Z = 2.468, P = 0.014)							0.15 [0.03, 0.28]

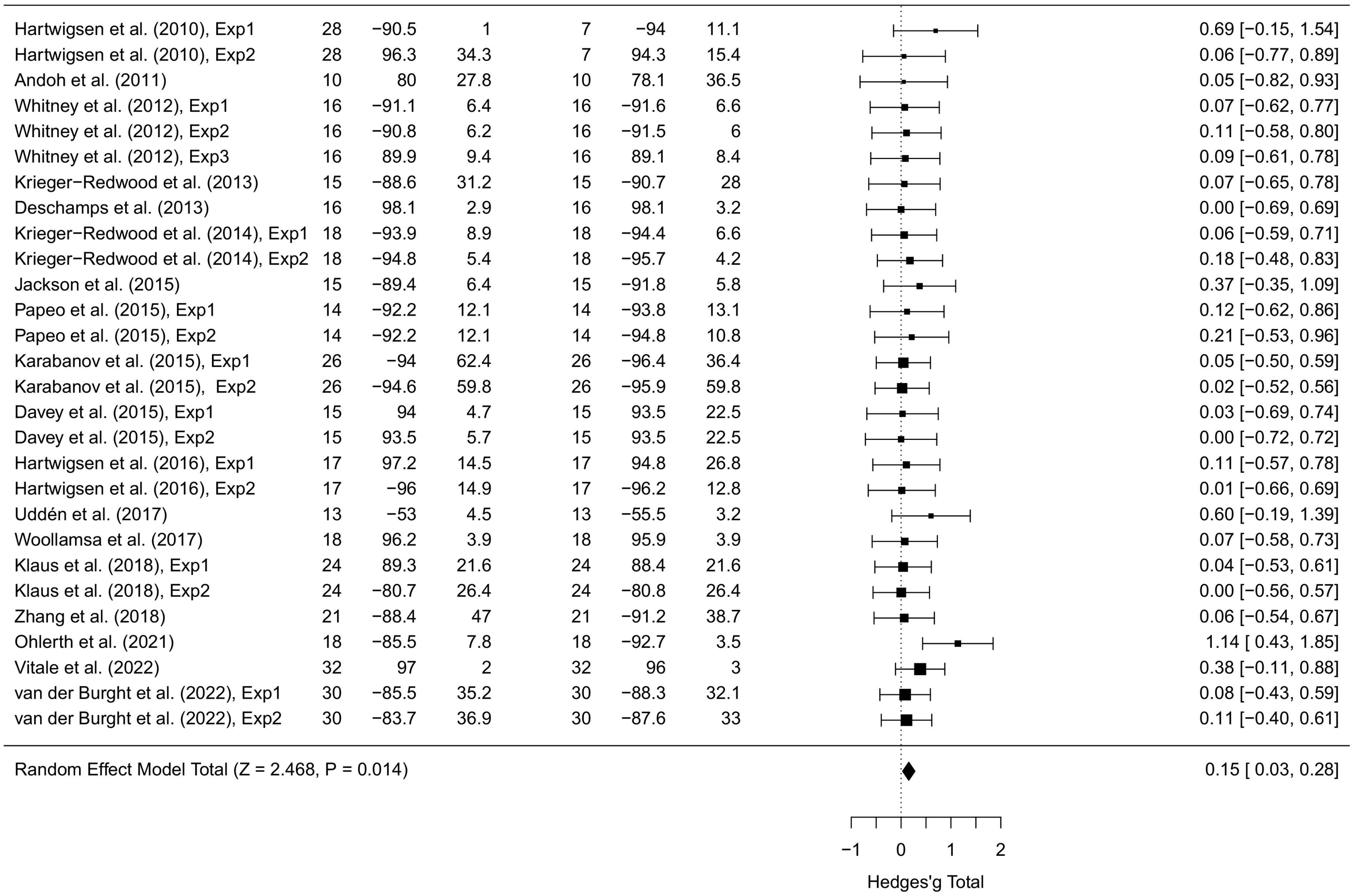

Supplementary Figure S42. Forest plot of ACC effect moderator-analysis of rTMS

	Experimental			Control			
Author(s) and Year	Total	Mean	SD	Total	Mean	SD	Hedges'g [95% CI]
Hartwigsena et al. (2013), Exp1	17	−98.5	0.6	17	−98.6	0.1	0.22 [−0.45, 0.90]
Hartwigsena et al. (2013), Exp2	17	98.5	0.6	17	98.5	0.8	0.00 [−0.67, 0.67]
Bonnì et al. (2015)	18	87.4	6.9	18	86.5	5.6	0.14 [−0.52, 0.79]
Hartwigsen et al. (2016), Exp1	17	97.3	13.3	17	94.7	30.3	0.11 [−0.57, 0.78]
Klaus et al. (2019)	16	−97	1.8	16	−98	1.5	0.57 [−0.13, 1.28]
Smalle et al. (2022)	18	68.8	63	18	57.3	61.2	0.18 [−0.48, 0.83]
Random Effect Model Total (Z = 1.398, P = 0.162)							0.20 [−0.08, 0.47]

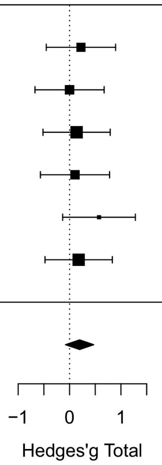

Supplementary Figure S43. Forest plot of ACC effect moderator-analysis of TBS

Author(s) and Year	Experimental Total	Experimental Mean	Experimental SD	Control Total	Control Mean	Control SD	Hedges'g [95% CI]
Hartwigsena et al. (2013), Exp1	17	−98.5	0.6	17	−98.6	0.1	0.22 [−0.45, 0.90]
Hartwigsena et al. (2013), Exp2	17	98.5	0.6	17	98.5	0.8	0.00 [−0.67, 0.67]
Bonnì et al. (2015)	18	87.4	6.9	18	86.5	5.6	0.14 [−0.52, 0.79]
Hartwigsen et al. (2016), Exp1	17	97.3	13.3	17	94.7	30.3	0.11 [−0.57, 0.78]
Klaus et al. (2019)	16	−97	1.8	16	−98	1.5	0.57 [−0.13, 1.28]
Smalle et al. (2022)	18	68.8	63	18	57.3	61.2	0.18 [−0.48, 0.83]
Random Effect Model Total (Z = 1.398, P = 0.162)							0.20 [−0.08, 0.47]

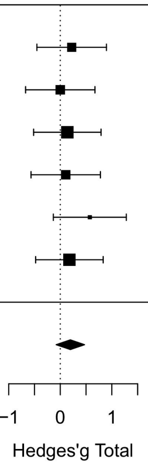

Supplementary Figure S44. Forest plot of ACC effect moderator-analysis of cTBS

Author(s) and Year	Experimental Total	Experimental Mean	Experimental SD	Control Total	Control Mean	Control SD	Hedges'g [95% CI]
Hartwigsena et al. (2013), Exp1	17	98.5	0.6	17	98.6	0.1	−0.22 [−0.90, 0.45]
Hartwigsena et al. (2013), Exp2	17	98.5	0.6	17	98.5	0.8	0.00 [−0.67, 0.67]
Bonnì et al. (2015)	18	87.4	6.9	18	86.5	5.6	0.14 [−0.52, 0.79]
Klaus et al. (2019)	16	97	1.8	16	98	1.5	−0.57 [−1.28, 0.13]
Smalle et al. (2022)	18	68.8	63	18	57.3	61.2	0.18 [−0.48, 0.83]
Random Effect Model Total (Z = −0.529, P = 0.597)							−0.08 [−0.38, 0.22]

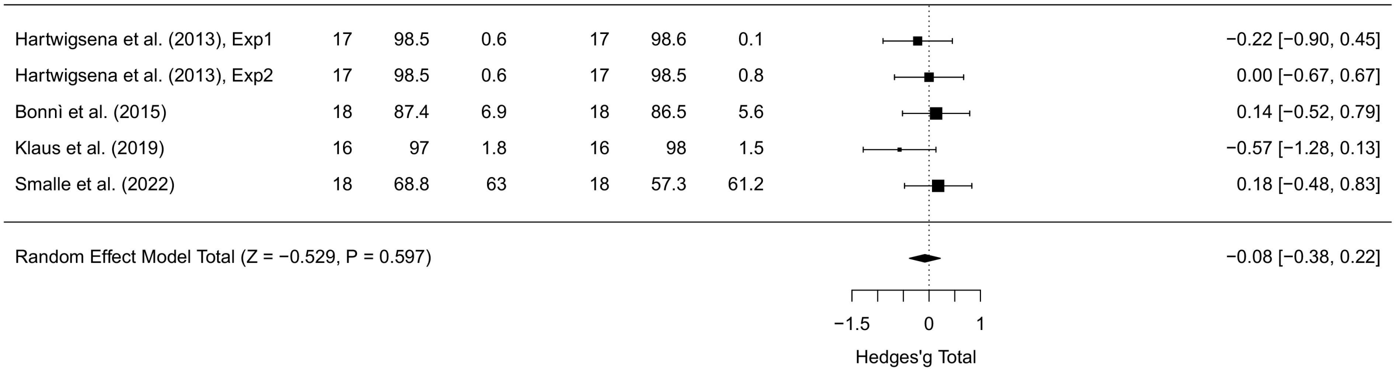

Supplementary Figure S45. Forest plot of supplementary ACC effect moderator-analysis of cTBS

Author(s) and Year	Experimental Total	Experimental Mean	Experimental SD	Control Total	Control Mean	Control SD	Hedges'g [95% CI]
Hartwigsen et al. (2010), Exp2	14	96.3	17.1	8	94.3	17.6	0.11 [−0.76, 0.98]
Franzmeier et al. (2012), Exp1	59	90.2	11.9	59	89.2	13	0.08 [−0.28, 0.44]
Franzmeier et al. (2012), Exp2	59	−89.1	12.3	59	−89.2	13	0.01 [−0.35, 0.37]
Karabanov et al. (2015), Exp1	26	−94	62.4	26	−96.4	36.4	0.05 [−0.50, 0.59]
Karabanov et al. (2015), Exp2	26	−94.6	59.8	26	−95.9	59.8	0.02 [−0.52, 0.56]
Hartwigsen et al. (2016), Exp1	17	97.2	14.5	17	94.8	26.8	0.11 [−0.57, 0.78]
Hartwigsen et al. (2016), Exp2	17	−96	14.9	17	−96.2	12.8	0.01 [−0.66, 0.69]
Klaus et al. (2018), Exp1	24	89.3	21.6	24	88.4	21.6	0.04 [−0.53, 0.61]
Klaus et al. (2018), Exp2	24	−80.7	26.4	24	−80.8	26.4	0.00 [−0.56, 0.57]
Zhang et al. (2018)	21	−88.4	47	21	−91.2	38.7	0.06 [−0.54, 0.67]
Ohlerth et al. (2021)	18	−85.5	7.8	18	−92.7	3.5	1.14 [0.43, 1.85]
Finocchiaro et al. (2021)	16	89.4	56.9	16	87.3	58	0.03 [−0.66, 0.73]
van der Burght et al. (2022), Exp1	30	−85.5	35.2	30	−88.3	32.1	0.08 [−0.43, 0.59]
van der Burght et al. (2022), Exp2	30	−83.7	36.9	30	−87.6	33	0.11 [−0.40, 0.61]
Random Effect Model Total (Z = 1.32, P = 0.187)							0.10 [−0.05, 0.24]

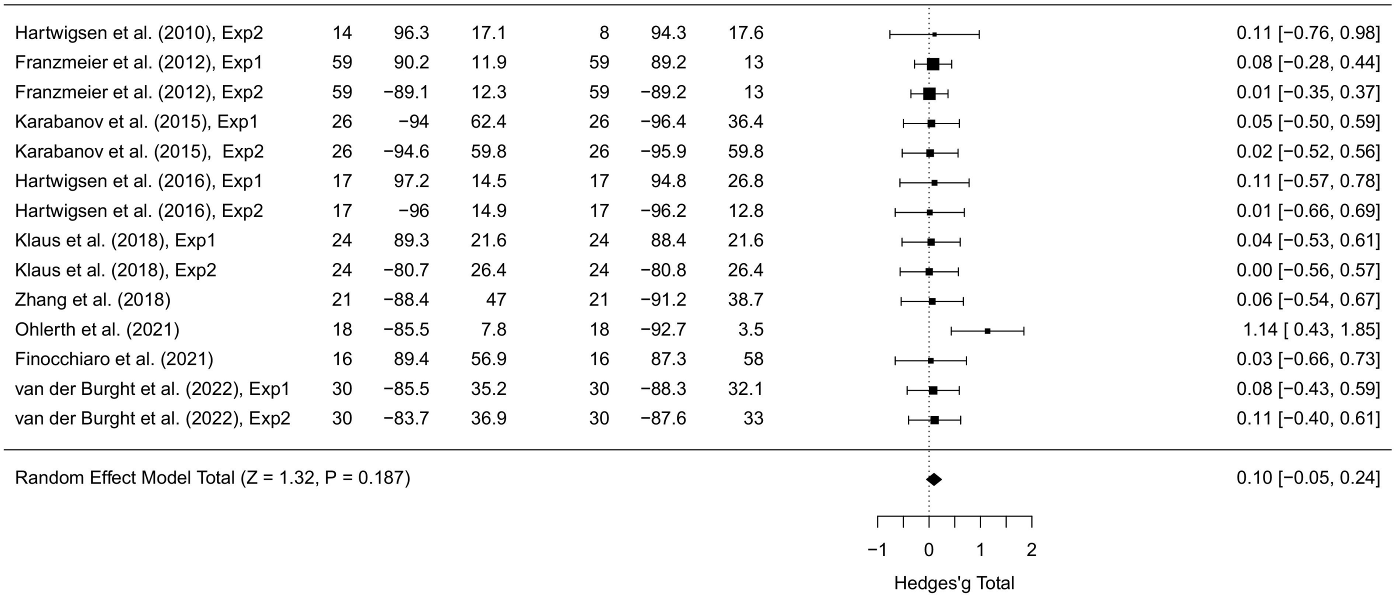

Supplementary Figure S46. Forest plot of ACC effect moderator-analysis of online TMS

Author(s) and Year	Experimental Total	Experimental Mean	Experimental SD	Control Total	Control Mean	Control SD	Hedges'g [95% CI]
Andoh et al. (2011)	10	80	27.8	10	78.1	36.5	0.05 [−0.82, 0.93]
Whitney et al. (2012), Exp1	16	−91.1	6.4	16	−91.6	6.6	0.07 [−0.62, 0.77]
Whitney et al. (2012), Exp2	16	−90.8	6.2	16	−91.5	6	0.11 [−0.58, 0.80]
Whitney et al. (2012), Exp3	16	89.9	9.4	16	89.1	8.4	0.09 [−0.61, 0.78]
Krieger−Redwood et al. (2013)	15	−88.6	31.2	15	−90.7	28	0.07 [−0.65, 0.78]
Hartwigsena et al. (2013), Exp1	17	−98.5	0.6	17	−98.6	0.1	0.22 [−0.45, 0.90]
Hartwigsena et al. (2013), Exp2	17	98.5	0.6	17	98.5	0.8	0.00 [−0.67, 0.67]
Deschamps et al. (2013)	16	98.1	2.9	16	98.1	3.2	0.00 [−0.69, 0.69]
Krieger−Redwood et al. (2014), Exp1	18	−93.9	8.9	18	−94.4	6.6	0.06 [−0.59, 0.71]
Krieger−Redwood et al. (2014), Exp2	18	−94.8	5.4	18	−95.7	4.2	0.18 [−0.48, 0.83]
Jackson et al. (2015)	15	−89.4	6.4	15	−91.8	5.8	0.37 [−0.35, 1.09]
Papeo et al. (2015), Exp1	14	−92.2	12.1	14	−93.8	13.1	0.12 [−0.62, 0.86]
Papeo et al. (2015), Exp2	14	−92.2	12.1	14	−94.8	10.8	0.21 [−0.53, 0.96]
Bonnì et al. (2015)	18	87.4	6.9	18	86.5	5.6	0.14 [−0.52, 0.79]
Davey et al. (2015), Exp1	15	94	4.7	15	93.5	22.5	0.03 [−0.69, 0.74]
Davey et al. (2015), Exp2	15	93.5	5.7	15	93.5	22.5	0.00 [−0.72, 0.72]
Hartwigsen et al. (2016), Exp1	17	97.2	14.5	17	94.8	26.8	0.11 [−0.57, 0.78]
Hartwigsen et al. (2016), Exp2	17	−96	14.9	17	−96.2	12.8	0.01 [−0.66, 0.69]
Uddén et al. (2017)	13	−53	4.5	13	−55.5	3.2	0.60 [−0.19, 1.39]
Woollamsa et al. (2017)	18	96.2	3.9	18	95.9	3.9	0.07 [−0.58, 0.73]
Klaus et al. (2019)	16	−97	1.8	16	−98	1.5	0.57 [−0.13, 1.28]
Vitale et al. (2022)	32	97	2	32	96	3	0.38 [−0.11, 0.88]
Smalle et al. (2022)	18	68.8	63	18	57.3	61.2	0.18 [−0.48, 0.83]
Random Effect Model Total (Z = 2.242, P = 0.025)							0.16 [0.02, 0.31]

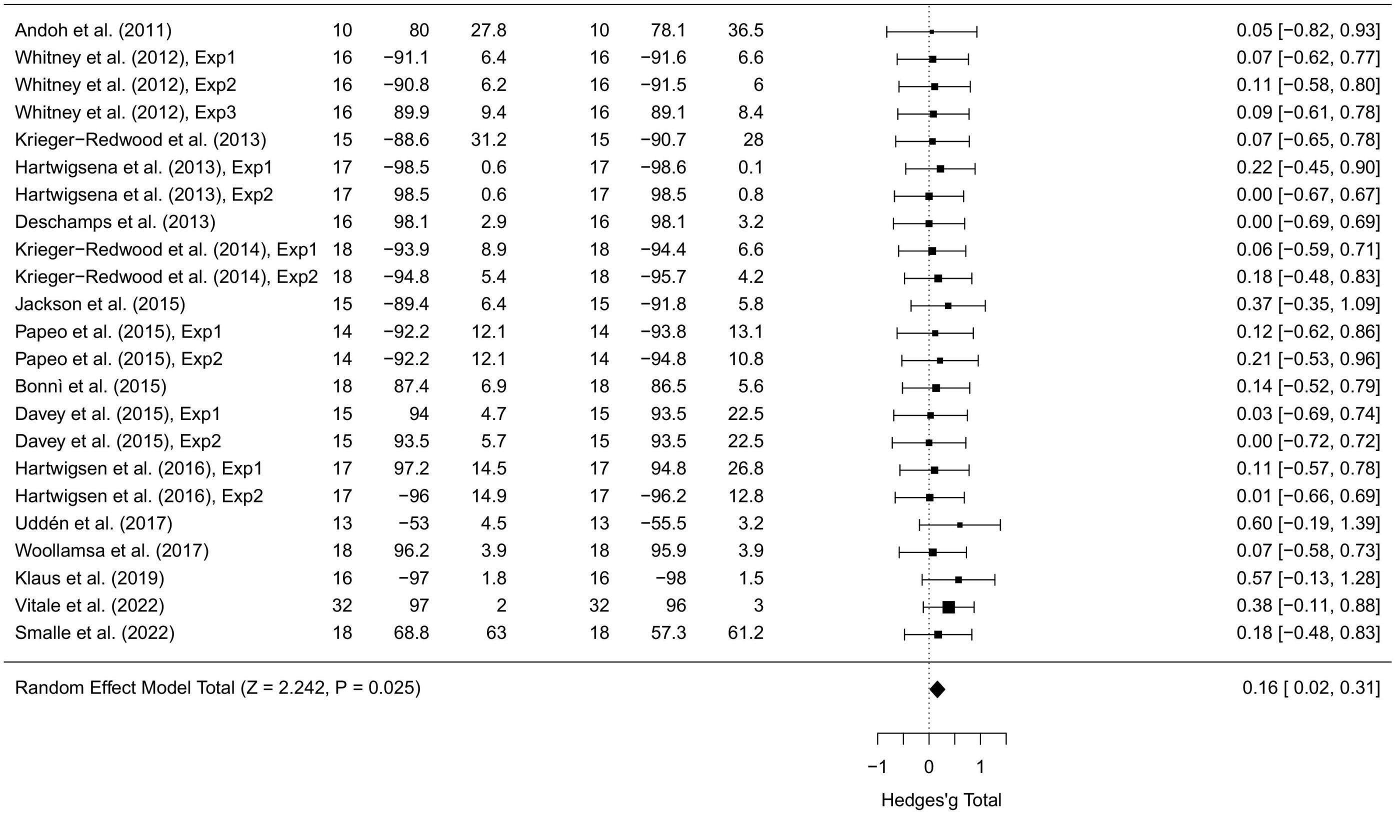

Supplementary Figure S47. Forest plot of ACC effect moderator-analysis of offline TMS

Author(s) and Year	Experimental Total	Experimental Mean	Experimental SD	Control Total	Control Mean	Control SD	Hedges'g [95% CI]
Hartwigsen et al. (2010), Exp1	28	−90.5	1	7	−94	11.1	0.69 [−0.15, 1.54]
Hartwigsen et al. (2010), Exp2	28	−96.3	34.3	7	−94.3	15.4	−0.06 [−0.89, 0.77]
Andoh et al. (2011)	10	−80	27.8	10	−78.1	36.5	−0.05 [−0.93, 0.82]
Hartwigsena et al. (2013), Exp1	17	−98.5	0.6	17	−98.6	0.1	0.22 [−0.45, 0.90]
Hartwigsena et al. (2013), Exp2	17	−98.5	0.6	17	−98.5	0.8	0.00 [−0.67, 0.67]
Bonnì et al. (2015)	18	−87.4	6.9	18	−86.5	5.6	−0.14 [−0.79, 0.52]
Karabanov et al. (2015), Exp1	26	−94	62.4	26	−96.4	36.4	0.05 [−0.50, 0.59]
Karabanov et al. (2015), Exp2	26	−94.6	59.8	26	−95.9	59.8	0.02 [−0.52, 0.56]
Hartwigsen et al. (2016), Exp1	17	−97.2	14.5	17	−94.8	26.8	−0.11 [−0.78, 0.57]
Hartwigsen et al. (2016), Exp2	17	−96	14.9	17	−96.2	12.8	0.01 [−0.66, 0.69]
Klaus et al. (2018), Exp1	24	−89.3	21.6	24	−88.4	21.6	−0.04 [−0.61, 0.53]
Klaus et al. (2018), Exp2	24	−80.7	26.4	24	−80.8	26.4	0.00 [−0.56, 0.57]
Zhang et al. (2018)	21	−88.4	47	21	−91.2	38.7	0.06 [−0.54, 0.67]
Klaus et al. (2019)	16	−97	1.8	16	−98	1.5	0.57 [−0.13, 1.28]
Ohlerth et al. (2021)	18	−85.5	7.8	18	−92.7	3.5	1.14 [0.43, 1.85]
Smalle et al. (2022)	18	−68.8	63	18	−57.3	61.2	−0.18 [−0.83, 0.48]
van der Burght et al. (2022), Exp1	30	−85.5	35.2	30	−88.3	32.1	0.08 [−0.43, 0.59]
van der Burght et al. (2022), Exp2	30	−83.7	36.9	30	−87.6	33	0.11 [−0.40, 0.61]
Random Effect Model Total (Z = 1.402, P = 0.161)							0.11 [−0.04, 0.26]

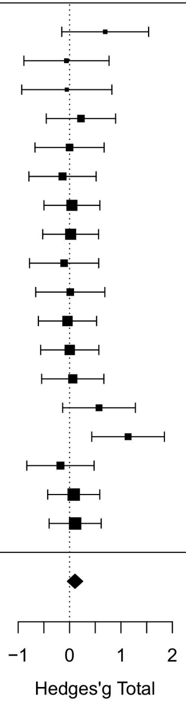

Supplementary Figure S48. Forest plot of ACC effect moderator-analysis of high frequency TMS

Author(s) and Year	Experimental Total	Experimental Mean	Experimental SD	Control Total	Control Mean	Control SD	Hedges'g [95% CI]
Whitney et al. (2012), Exp1	16	−91.1	6.4	16	−91.6	6.6	0.07 [−0.62, 0.77]
Whitney et al. (2012), Exp2	16	−90.8	6.2	16	−91.5	6	0.11 [−0.58, 0.80]
Whitney et al. (2012), Exp3	16	89.9	9.4	16	89.1	8.4	0.09 [−0.61, 0.78]
Krieger−Redwood et al. (2013)	15	−88.6	31.2	15	−90.7	28	0.07 [−0.65, 0.78]
Deschamps et al. (2013)	16	98.1	2.9	16	98.1	3.2	0.00 [−0.69, 0.69]
Krieger−Redwood et al. (2014), Exp1	18	−93.9	8.9	18	−94.4	6.6	0.06 [−0.59, 0.71]
Krieger−Redwood et al. (2014), Exp2	18	−94.8	5.4	18	−95.7	4.2	0.18 [−0.48, 0.83]
Jackson et al. (2015)	15	−89.4	6.4	15	−91.8	5.8	0.37 [−0.35, 1.09]
Papeo et al. (2015), Exp1	14	−92.2	12.1	14	−93.8	13.1	0.12 [−0.62, 0.86]
Papeo et al. (2015), Exp2	14	−92.2	12.1	14	−94.8	10.8	0.21 [−0.53, 0.96]
Davey et al. (2015), Exp1	15	94	4.7	15	93.5	22.5	0.03 [−0.69, 0.74]
Davey et al. (2015), Exp2	15	93.5	5.7	15	93.5	22.5	0.00 [−0.72, 0.72]
Uddén et al. (2017)	13	−53	4.5	13	−55.5	3.2	0.60 [−0.19, 1.39]
Woollamsa et al. (2017)	18	96.2	3.9	18	95.9	3.9	0.07 [−0.58, 0.73]
Vitale et al. (2022)	32	97	2	32	96	3	0.38 [−0.11, 0.88]
Random Effect Model Total (Z = 1.833, P = 0.067)							0.16 [−0.01, 0.34]

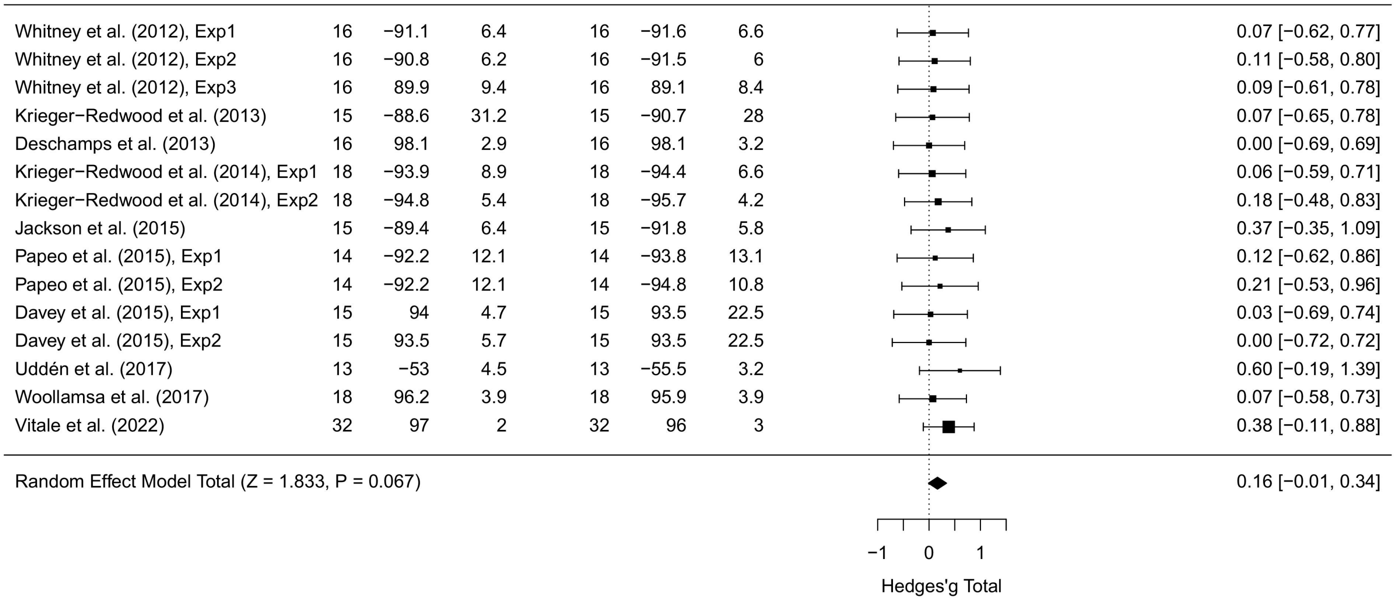

Supplementary Figure S49. Forest plot of ACC effect moderator-analysis of low frequency TMS

Author(s) and Year	Experimental Total	Experimental Mean	Experimental SD	Control Total	Control Mean	Control SD	Hedges'g [95% CI]
Hartwigsen et al. (2010), Exp1	28	90.5	1	7	94	11.1	−0.69 [−1.54, 0.15]
Hartwigsen et al. (2010), Exp2	28	96.3	34.3	7	94.3	15.4	0.06 [−0.77, 0.89]
Andoh et al. (2011)	10	80	27.8	10	78.1	36.5	0.05 [−0.82, 0.93]
Hartwigsena et al. (2013), Exp1	17	98.5	0.6	17	98.6	0.1	−0.22 [−0.90, 0.45]
Hartwigsena et al. (2013), Exp2	17	98.5	0.6	17	98.5	0.8	0.00 [−0.67, 0.67]
Bonnì et al. (2015)	18	87.4	6.9	18	86.5	5.6	0.14 [−0.52, 0.79]
Karabanov et al. (2015), Exp1	26	94	62.4	26	96.4	36.4	−0.05 [−0.59, 0.50]
Karabanov et al. (2015), Exp2	26	94.6	59.8	26	95.9	59.8	−0.02 [−0.56, 0.52]
Hartwigsen et al. (2016), Exp1	17	97.2	14.5	17	94.8	26.8	0.11 [−0.57, 0.78]
Hartwigsen et al. (2016), Exp2	17	96	14.9	17	96.2	12.8	−0.01 [−0.69, 0.66]
Klaus et al. (2018), Exp1	24	89.3	21.6	24	88.4	21.6	0.04 [−0.53, 0.61]
Klaus et al. (2018), Exp2	24	80.7	26.4	24	80.8	26.4	−0.00 [−0.57, 0.56]
Zhang et al. (2018)	21	88.4	47	21	91.2	38.7	−0.06 [−0.67, 0.54]
Klaus et al. (2019)	16	97	1.8	16	98	1.5	−0.57 [−1.28, 0.13]
Ohlerth et al. (2021)	18	85.5	7.8	18	92.7	3.5	−1.14 [−1.85, −0.43]
Smalle et al. (2022)	18	68.8	63	18	57.3	61.2	0.18 [−0.48, 0.83]
van der Burght et al. (2022), Exp1	30	85.5	35.2	30	88.3	32.1	−0.08 [−0.59, 0.43]
van der Burght et al. (2022), Exp2	30	83.7	36.9	30	87.6	33	−0.11 [−0.61, 0.40]
Random Effect Model Total (Z = −1.402, P = 0.161)							−0.11 [−0.26, 0.04]

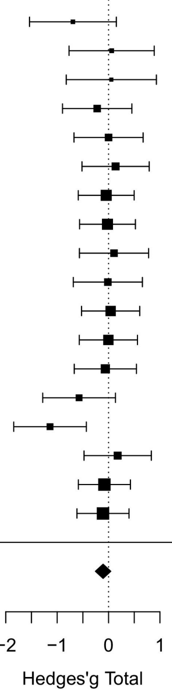

***Supplementary Figure S50.** Forest plot of ACC effect moderator-analysis with original values of high frequency TMS

Author(s) and Year	Experimental Total	Experimental Mean	Experimental SD	Control Total	Control Mean	Control SD	Hedges'g [95% CI]
Whitney et al. (2012), Exp1	16	91.1	6.4	16	91.6	6.6	−0.07 [−0.77, 0.62]
Whitney et al. (2012), Exp2	16	90.8	6.2	16	91.5	6	−0.11 [−0.80, 0.58]
Whitney et al. (2012), Exp3	16	89.9	9.4	16	89.1	8.4	0.09 [−0.61, 0.78]
Krieger−Redwood et al. (2013)	15	88.6	31.2	15	90.7	28	−0.07 [−0.78, 0.65]
Deschamps et al. (2013)	16	98.1	2.9	16	98.1	3.2	0.00 [−0.69, 0.69]
Krieger−Redwood et al. (2014), Exp1	18	93.9	8.9	18	94.4	6.6	−0.06 [−0.71, 0.59]
Krieger−Redwood et al. (2014), Exp2	18	94.8	5.4	18	95.7	4.2	−0.18 [−0.83, 0.48]
Jackson et al. (2015)	15	89.4	6.4	15	91.8	5.8	−0.37 [−1.09, 0.35]
Papeo et al. (2015), Exp1	14	92.2	12.1	14	93.8	13.1	−0.12 [−0.86, 0.62]
Papeo et al. (2015), Exp2	14	92.2	12.1	14	94.8	10.8	−0.21 [−0.96, 0.53]
Davey et al. (2015), Exp1	15	94	4.7	15	93.5	22.5	0.03 [−0.69, 0.74]
Davey et al. (2015), Exp2	15	93.5	5.7	15	93.5	22.5	0.00 [−0.72, 0.72]
Uddén et al. (2017)	13	53	4.5	13	55.5	3.2	−0.60 [−1.39, 0.19]
Woollamsa et al. (2017)	18	96.2	3.9	18	95.9	3.9	0.07 [−0.58, 0.73]
Vitale et al. (2022)	32	97	2	32	96	3	0.38 [−0.11, 0.88]
Random Effect Model Total (Z = 0.299, P = 0.765)							0.05 [−0.28, 0.38]

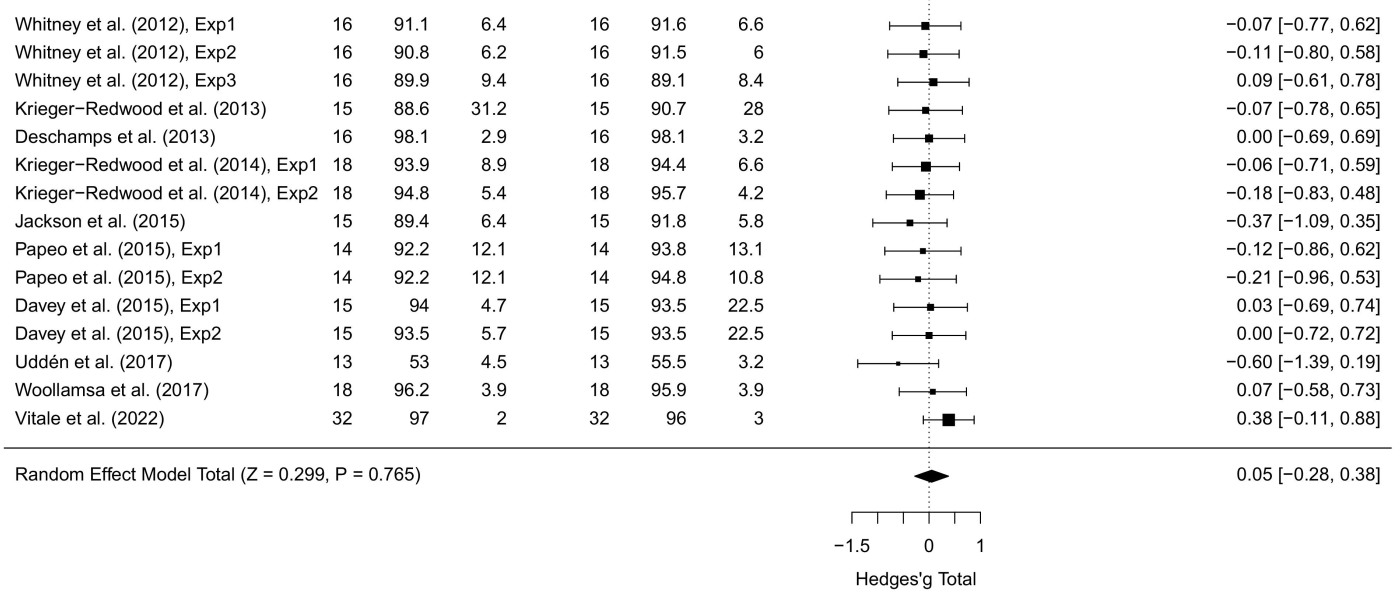

*__Supplementary Figure S51.__ Forest plot of ACC effect moderator-analysis with original values of low frequency TMS

Author(s) and Year	Experimental Total	Experimental Mean	Experimental SD	Control Total	Control Mean	Control SD	Hedges'g [95% CI]
Hartwigsen et al. (2010), Exp1	28	−90.5	1	7	−94	11.1	0.69 [−0.15, 1.54]
Hartwigsen et al. (2010), Exp2	28	96.3	34.3	7	94.3	15.4	0.06 [−0.77, 0.89]
Whitney et al. (2012), Exp1	16	−91.1	6.4	16	−91.6	6.6	0.07 [−0.62, 0.77]
Whitney et al. (2012), Exp2	16	−90.8	6.2	16	−91.5	6	0.11 [−0.58, 0.80]
Whitney et al. (2012), Exp3	16	89.9	9.4	16	89.1	8.4	0.09 [−0.61, 0.78]
Hartwigsena et al. (2013), Exp1	17	−98.5	0.6	17	−98.6	0.1	0.22 [−0.45, 0.90]
Hartwigsena et al. (2013), Exp2	17	98.5	0.6	17	98.5	0.8	0.00 [−0.67, 0.67]
Bonnì et al. (2015)	18	87.4	6.9	18	86.5	5.6	0.14 [−0.52, 0.79]
Smalle et al. (2022)	18	68.8	63	18	57.3	61.2	0.18 [−0.48, 0.83]
Random Effect Model Total (Z = 1.304, P = 0.192)							0.16 [−0.08, 0.39]

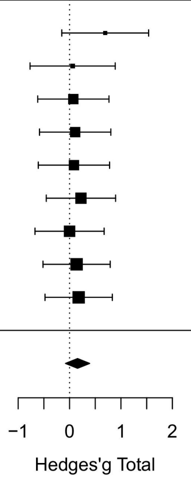

Supplementary Figure S52. Forest plot of ACC effect moderator-analysis of AMT (a type of intensity)

Author(s) and Year	Experimental Total	Experimental Mean	Experimental SD	Control Total	Control Mean	Control SD	Hedges'g [95% CI]
Deschamps et al. (2013)	16	98.1	2.9	16	98.1	3.2	0.00 [−0.69, 0.69]
Karabanov et al. (2015), Exp1	26	−94	62.4	26	−96.4	36.4	0.05 [−0.50, 0.59]
Karabanov et al. (2015), Exp2	26	−94.6	59.8	26	−95.9	59.8	0.02 [−0.52, 0.56]
Hartwigsen et al. (2016), Exp1	17	97.2	14.5	17	94.8	26.8	0.11 [−0.57, 0.78]
Hartwigsen et al. (2016), Exp2	17	−96	14.9	17	−96.2	12.8	0.01 [−0.66, 0.69]
Uddén et al. (2017)	13	−53	4.5	13	−55.5	3.2	0.60 [−0.19, 1.39]
Klaus et al. (2019)	16	−97	1.8	16	−98	1.5	0.57 [−0.13, 1.28]
Ohlerth et al. (2021)	18	−85.5	7.8	18	−92.7	3.5	1.14 [0.43, 1.85]
Vitale et al. (2022)	32	97	2	32	96	3	0.38 [−0.11, 0.88]
van der Burght et al. (2022), Exp1	30	−85.5	35.2	30	−88.3	32.1	0.08 [−0.43, 0.59]
van der Burght et al. (2022), Exp2	30	−83.7	36.9	30	−87.6	33	0.11 [−0.40, 0.61]
Random Effect Model Total (Z = 2.35, P = 0.019)							0.27 [0.04, 0.49]

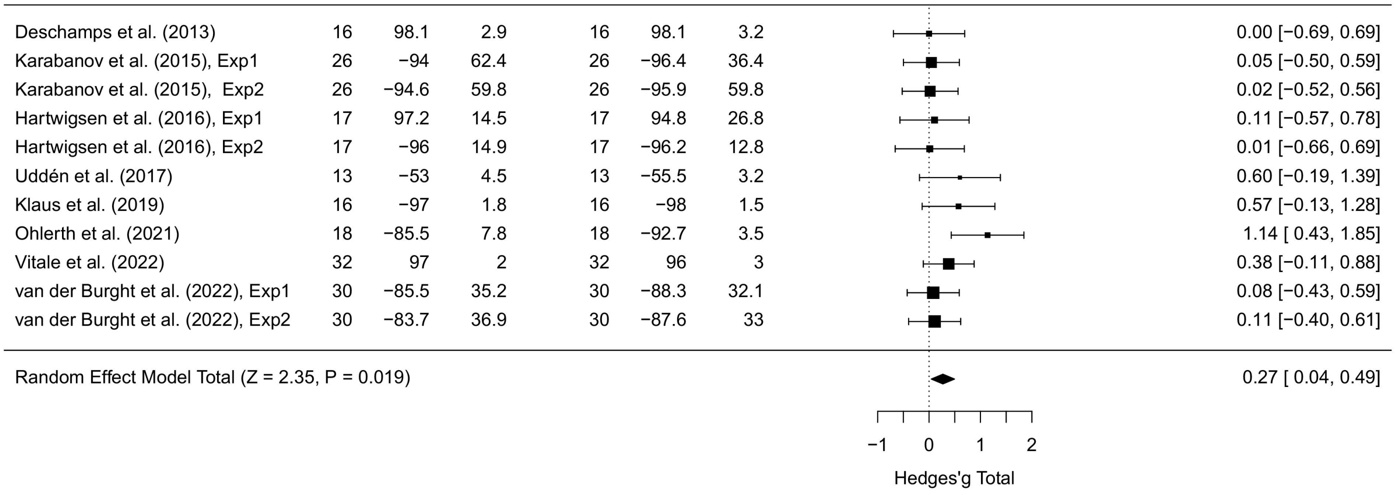

Supplementary Figure S53. Forest plot of ACC effect moderator-analysis of RMT (a type of intensity)

Author(s) and Year	Experimental Total	Experimental Mean	Experimental SD	Control Total	Control Mean	Control SD	Hedges'g [95% CI]
Hartwigsen et al. (2010), Exp1	28	−90.5	1	7	−94	11.1	0.69 [−0.15, 1.54]
Hartwigsen et al. (2010), Exp2	28	96.3	34.3	7	94.3	15.4	0.06 [−0.77, 0.89]
Deschamps et al. (2013)	16	98.1	2.9	16	98.1	3.2	0.00 [−0.69, 0.69]
Hartwigsen et al. (2016), Exp1	17	97.2	14.5	17	94.8	26.8	0.11 [−0.57, 0.78]
Zhang et al. (2018)	21	−88.4	47	21	−91.2	38.7	0.06 [−0.54, 0.67]
Klaus et al. (2019)	16	−97	1.8	16	−98	1.5	0.57 [−0.13, 1.28]
Finocchiaro et al. (2021)	16	89.4	56.9	16	87.3	58	0.03 [−0.66, 0.73]
Vitale et al. (2022)	32	97	2	32	96	3	0.38 [−0.11, 0.88]
Random Effect Model Total (Z = 1.959, P = 0.05)							0.24 [−0.00, 0.47]

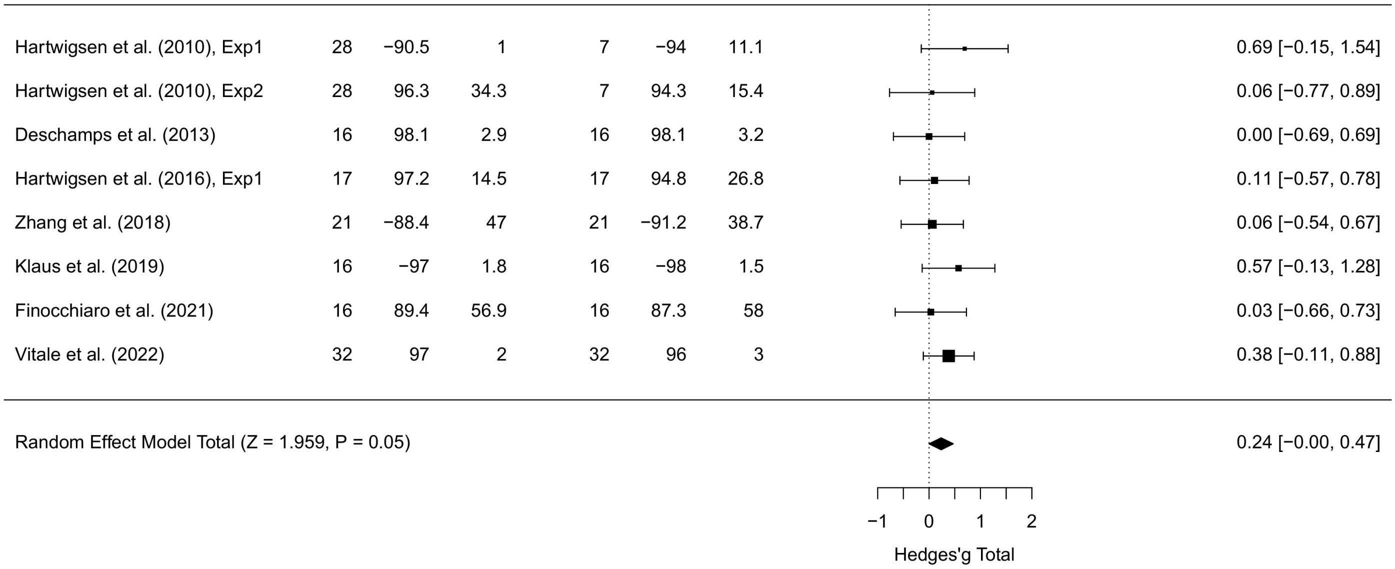

Supplementary Figure S54. Forest plot of ACC effect moderator-analysis of sham TMS (a type of control conditions)

Author(s) and Year	Experimental Total	Experimental Mean	Experimental SD	Control Total	Control Mean	Control SD	Hedges'g [95% CI]
Andoh et al. (2011)	10	80	27.8	10	78.1	36.5	0.05 [−0.82, 0.93]
Whitney et al. (2012), Exp1	16	−91.1	6.4	16	−91.6	6.6	0.07 [−0.62, 0.77]
Whitney et al. (2012), Exp2	16	−90.8	6.2	16	−91.5	6	0.11 [−0.58, 0.80]
Whitney et al. (2012), Exp3	16	89.9	9.4	16	89.1	8.4	0.09 [−0.61, 0.78]
Krieger−Redwood et al. (2013)	15	−88.6	31.2	15	−90.7	28	0.07 [−0.65, 0.78]
Krieger−Redwood et al. (2014), Exp1	18	−93.9	8.9	18	−94.4	6.6	0.06 [−0.59, 0.71]
Krieger−Redwood et al. (2014), Exp2	18	−94.8	5.4	18	−95.7	4.2	0.18 [−0.48, 0.83]
Papeo et al. (2015), Exp1	14	−92.2	12.1	14	−93.8	13.1	0.12 [−0.62, 0.86]
Woollamsa et al. (2017)	18	96.2	3.9	18	95.9	3.9	0.07 [−0.58, 0.73]
Ohlerth et al. (2021)	18	−85.5	7.8	18	−92.7	3.5	1.14 [0.43, 1.85]
Random Effect Model Total (Z = 1.714, P = 0.087)							0.20 [−0.03, 0.42]

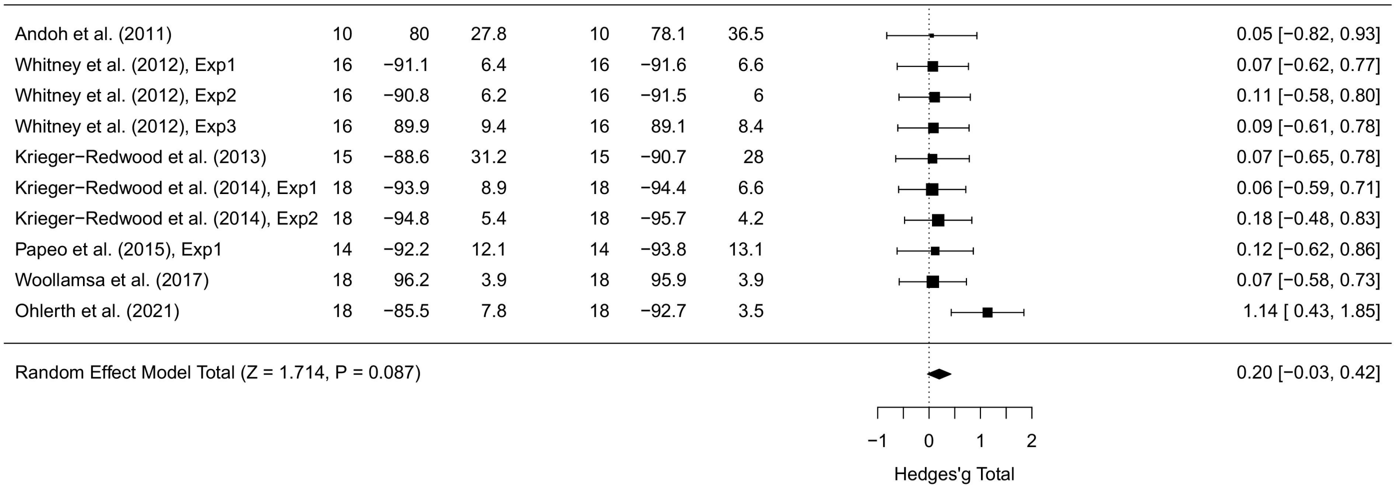

Supplementary Figure S55. Forest plot of ACC effect moderator-analysis of no TMS (a type of control conditions)

Author(s) and Year	Experimental Total	Experimental Mean	Experimental SD	Control Total	Control Mean	Control SD	Hedges'g [95% CI]
Franzmeier et al. (2012), Exp1	59	90.2	11.9	59	89.2	13	0.08 [−0.28, 0.44]
Franzmeier et al. (2012), Exp2	59	−89.1	12.3	59	−89.2	13	0.01 [−0.35, 0.37]
Bonnì et al. (2015)	18	87.4	6.9	18	86.5	5.6	0.14 [−0.52, 0.79]
Davey et al. (2015), Exp1	15	94	4.7	15	93.5	22.5	0.03 [−0.69, 0.74]
Davey et al. (2015), Exp2	15	93.5	5.7	15	93.5	22.5	0.00 [−0.72, 0.72]
Klaus et al. (2018), Exp1	24	89.3	21.6	24	88.4	21.6	0.04 [−0.53, 0.61]
Klaus et al. (2018), Exp2	24	−80.7	26.4	24	−80.8	26.4	0.00 [−0.56, 0.57]
Vitale et al. (2022)	32	97	2	32	96	3	0.38 [−0.11, 0.88]
Smalle et al. (2022)	18	68.8	63	18	57.3	61.2	0.18 [−0.48, 0.83]
van der Burght et al. (2022), Exp1	30	−85.5	35.2	30	−88.3	32.1	0.08 [−0.43, 0.59]
van der Burght et al. (2022), Exp2	30	−83.7	36.9	30	−87.6	33	0.11 [−0.40, 0.61]
Random Effect Model Total (Z = 1.061, P = 0.289)							0.12 [−0.10, 0.33]

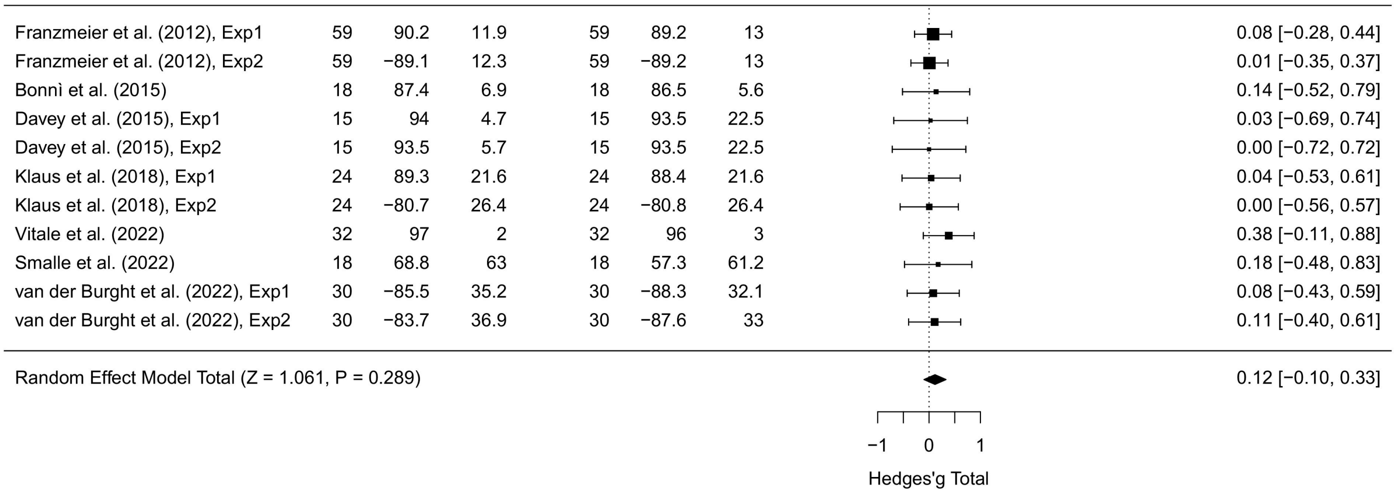

Supplementary Figure S56. Forest plot of ACC effect moderator-analysis of vertex (a type of control conditions)

Author(s) and Year	Experimental Total	Experimental Mean	Experimental SD	Control Total	Control Mean	Control SD	Hedges'g [95% CI]
Krieger−Redwood et al. (2013), Exp1	15	−88.7	28.1	15	−91.3	29.5	0.09 [−0.63, 0.80]
Krieger−Redwood et al. (2013), Exp2	15	−86.9	36.3	15	−89.7	28.4	0.08 [−0.63, 0.80]
Krieger−Redwood et al. (2013), Exp3	15	−90.1	28.6	15	−91.2	26.1	0.04 [−0.68, 0.75]
Papeo et al. (2015), Exp2	14	−92.2	12.1	14	−94.8	10.8	0.21 [−0.53, 0.96]
Karabanov et al. (2015), Exp1	26	−94	62.4	26	−96.4	36.4	0.05 [−0.50, 0.59]
Karabanov et al. (2015), Exp2	26	−94.6	59.8	26	−95.9	59.8	0.02 [−0.52, 0.56]
Random Effect Model Total (Z = 0.522, P = 0.602)							0.07 [−0.19, 0.33]

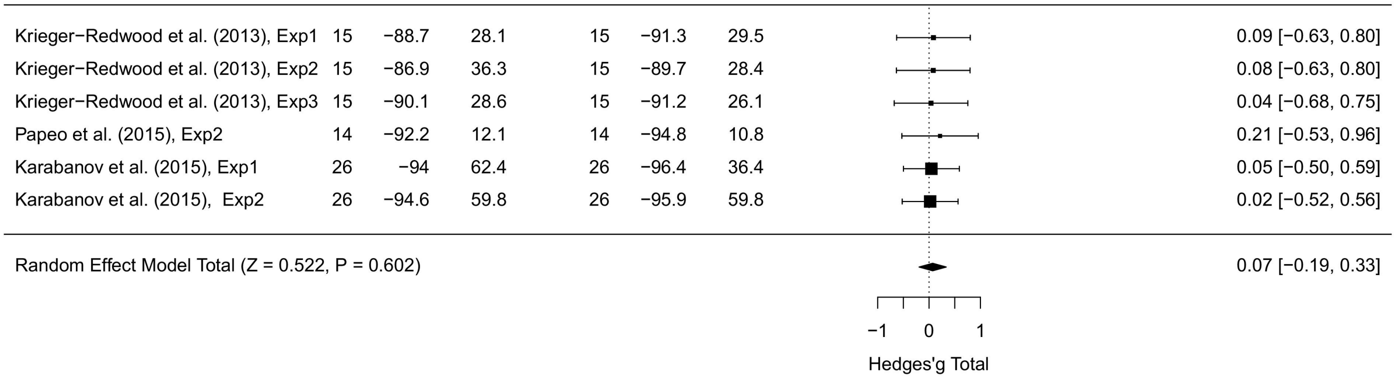

Supplementary Figure S57. Forest plot of ACC effect moderator-analysis of other brain region (a type of control conditions)

Author(s) and Year	Experimental Total	Experimental Mean	Experimental SD	Control Total	Control Mean	Control SD	Hedges'g [95% CI]
Hartwigsena et al. (2013), Exp1	17	−98.5	0.6	17	−98.6	0.1	0.22 [−0.45, 0.90]
Hartwigsena et al. (2013), Exp2	17	98.5	0.6	17	98.5	0.8	0.00 [−0.67, 0.67]
Jackson et al. (2015)	15	−89.4	6.4	15	−91.8	5.8	0.37 [−0.35, 1.09]
Hartwigsen et al. (2016), Exp2	17	−96	14.9	17	−96.2	12.8	0.01 [−0.66, 0.69]
Uddén et al. (2017)	13	−53	4.5	13	−55.5	3.2	0.60 [−0.19, 1.39]
Random Effect Model Total (Z = 1.353, P = 0.176)							0.22 [−0.10, 0.53]

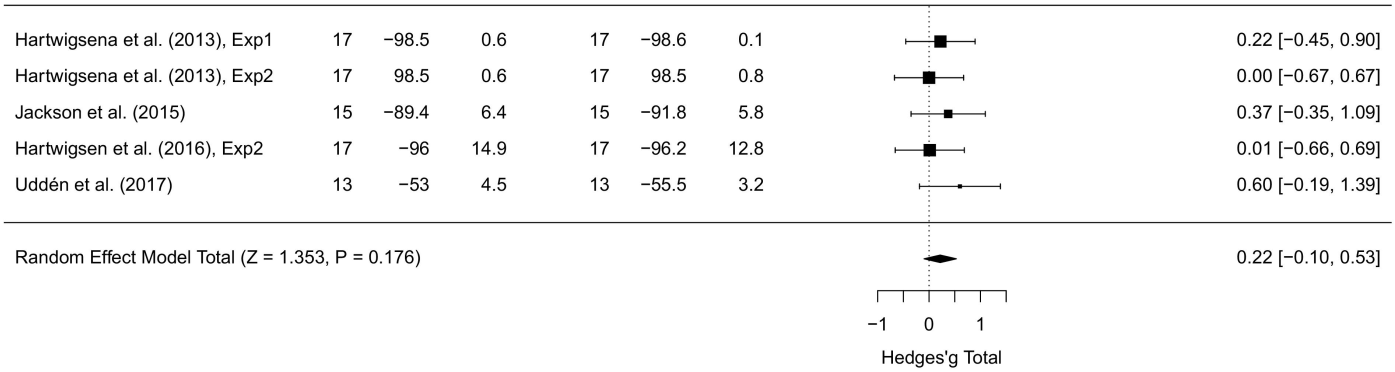

Supplementary Figure S58. Forest plot of ACC effect moderator-analysis of others (a type of control conditions)

Author(s) and Year	Experimental Total	Experimental Mean	Experimental SD	Control Total	Control Mean	Control SD	Hedges'g [95% CI]
Andoh et al. (2011), Exp1	10	82.5	29.1	10	77.2	32.1	0.16 [−0.72, 1.04]
Andoh et al. (2011), Exp2	10	−77.5	26.5	10	−79	40.4	0.04 [−0.84, 0.92]
Franzmeier et al. (2012), Exp1	59	90.2	11.9	59	89.2	13	0.08 [−0.28, 0.44]
Franzmeier et al. (2012), Exp2	59	−89.1	12.3	59	−89.2	13	0.01 [−0.35, 0.37]
Whitney et al. (2012), Exp1	16	−91.1	6.4	16	−91.6	6.6	0.07 [−0.62, 0.77]
Whitney et al. (2012), Exp2	16	−90.8	6.2	16	−91.5	6	0.11 [−0.58, 0.80]
Whitney et al. (2012), Exp3	16	89.9	9.4	16	89.1	8.4	0.09 [−0.61, 0.78]
Krieger−Redwood et al. (2013)	15	−88.6	31.2	15	−90.7	28	0.07 [−0.65, 0.78]
Hartwigsena et al. (2013), Exp1	17	−98.5	0.6	17	−98.6	0.1	0.22 [−0.45, 0.90]
Hartwigsena et al. (2013), Exp2	17	98.5	0.6	17	98.5	0.8	0.00 [−0.67, 0.67]
Deschamps et al. (2013)	16	98.1	2.9	16	98.1	3.2	0.00 [−0.69, 0.69]
Krieger−Redwood et al. (2014), Exp1	18	−93.9	8.9	18	−94.4	6.6	0.06 [−0.59, 0.71]
Krieger−Redwood et al. (2014), Exp2	18	−94.8	5.4	18	−95.7	4.2	0.18 [−0.48, 0.83]
Jackson et al. (2015)	15	−89.4	6.4	15	−91.8	5.8	0.37 [−0.35, 1.09]
Papeo et al. (2015), Exp1	14	−92.2	12.1	14	−93.8	13.1	0.12 [−0.62, 0.86]
Papeo et al. (2015), Exp2	14	−92.2	12.1	14	−94.8	10.8	0.21 [−0.53, 0.96]
Bonnì et al. (2015)	18	87.4	6.9	18	86.5	5.6	0.14 [−0.52, 0.79]
Hartwigsen et al. (2016), Exp1	17	97.2	14.5	17	94.8	26.8	0.11 [−0.57, 0.78]
Hartwigsen et al. (2016), Exp2	17	−96	14.9	17	−96.2	12.8	0.01 [−0.66, 0.69]
Uddén et al. (2017)	13	−53	4.5	13	−55.5	3.2	0.60 [−0.19, 1.39]
Klaus et al. (2018), Exp1	24	89.3	21.6	24	88.4	21.6	0.04 [−0.53, 0.61]
Klaus et al. (2018), Exp2	24	−80.7	26.4	24	−80.8	26.4	0.00 [−0.56, 0.57]
Zhang et al. (2018)	21	−88.4	47	21	−91.2	38.7	0.06 [−0.54, 0.67]
Klaus et al. (2019)	16	−97	1.8	16	−98	1.5	0.57 [−0.13, 1.28]
Ohlerth et al. (2021), Exp1	18	−86.1	7.1	18	−92.7	3.5	1.13 [0.42, 1.83]
Ohlerth et al. (2021), Exp2	18	−85	8.5	18	−92.7	3.5	1.13 [0.43, 1.84]
Random Effect Model Total (Z = 2.532, P = 0.011)							0.19 [0.04, 0.35]

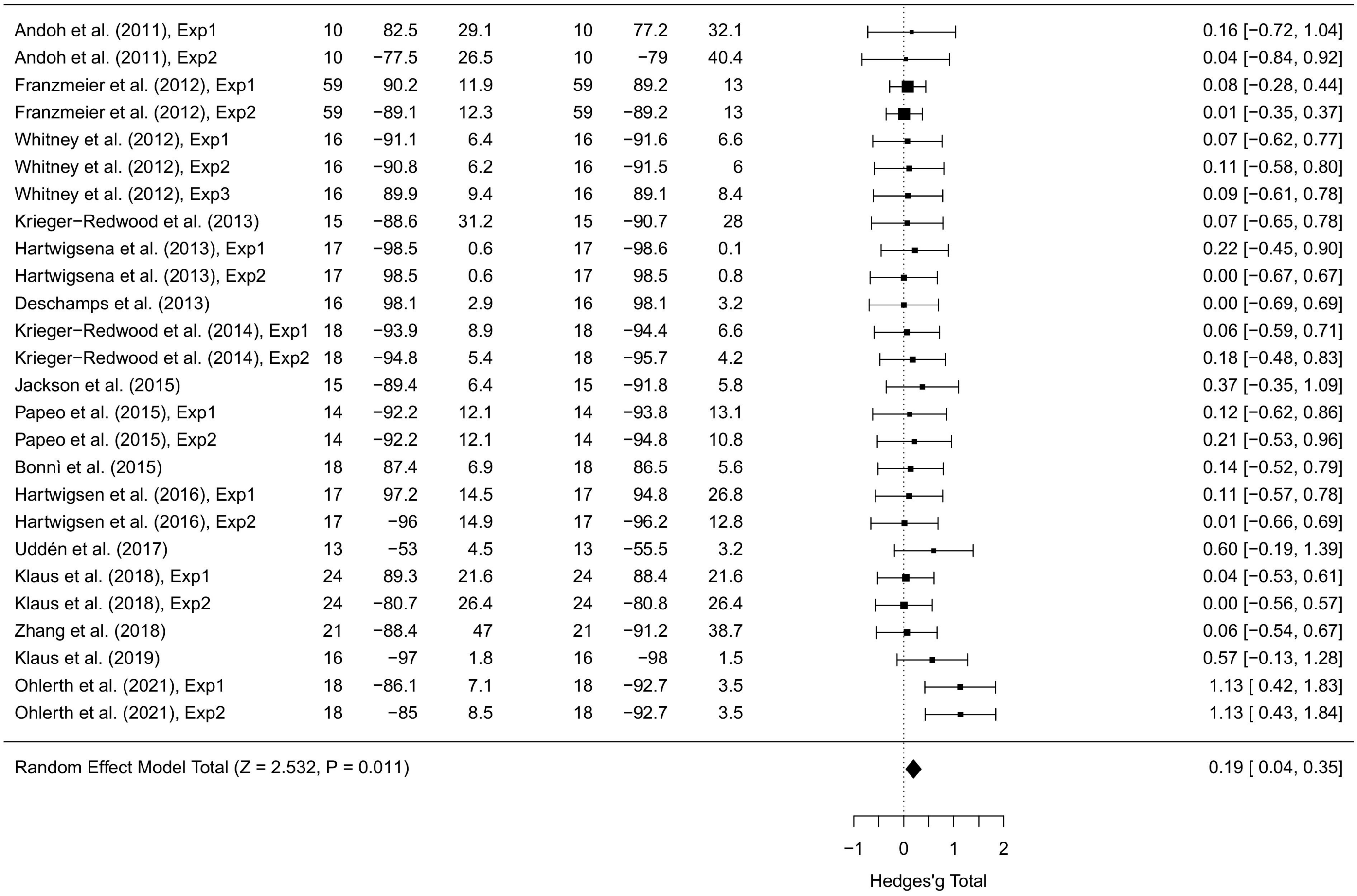

Supplementary Figure S59. Forest plot of ACC effect moderator-analysis of within-subject group design

Author(s) and Year	Experimental Total	Experimental Mean	Experimental SD	Control Total	Control Mean	Control SD	Hedges'g [95% CI]
Hartwigsen et al. (2010), Exp1	28	−90.5	1	7	−94	11.1	0.69 [−0.15, 1.54]
Hartwigsen et al. (2010), Exp2	28	96.3	34.3	7	94.3	15.4	0.06 [−0.77, 0.89]
Karabanov et al. (2015), Exp1	26	−94	62.4	26	−96.4	36.4	0.05 [−0.50, 0.59]
Karabanov et al. (2015), Exp2	26	−94.6	59.8	26	−95.9	59.8	0.02 [−0.52, 0.56]
Woollamsa et al. (2017)	18	96.2	3.9	18	95.9	3.9	0.07 [−0.58, 0.73]
Finocchiaro et al. (2021)	16	89.4	56.9	16	87.3	58	0.03 [−0.66, 0.73]
Vitale et al. (2022)	32	97	2	32	96	3	0.38 [−0.11, 0.88]
Smalle et al. (2022)	18	68.8	63	18	57.3	61.2	0.18 [−0.48, 0.83]
Random Effect Model Total (Z = 1.495, P = 0.135)							0.17 [−0.05, 0.40]

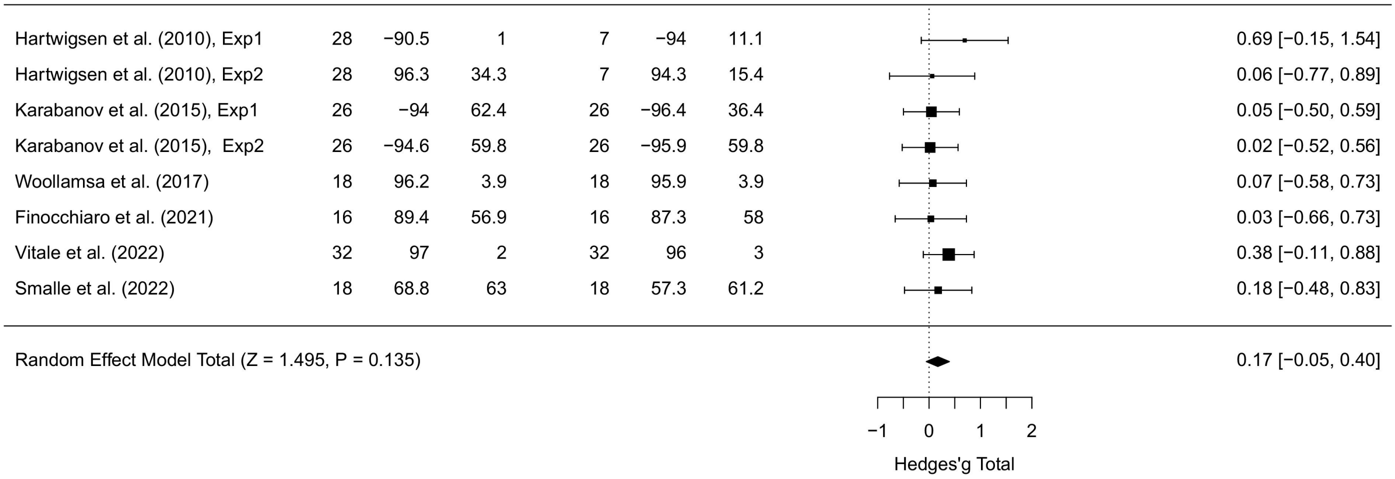

Supplementary Figure S60. Forest plot of ACC effect moderator-analysis of between-subject group design
